# Copper-promoted site-selective carbonylation of sp^3^ and sp^2^ C–H bonds with nitromethane†
†Electronic supplementary information (ESI) available: Experimental procedures and characterization data. See DOI: 10.1039/c6sc01087c


**DOI:** 10.1039/c6sc01087c

**Published:** 2016-04-26

**Authors:** Xuesong Wu, Jinmin Miao, Yanrong Li, Guigen Li, Haibo Ge

**Affiliations:** a Department of Chemistry and Chemical Biology , Indiana University Purdue University Indianapolis , Indianapolis IN 46202 , USA . Email: geh@iupui.edu; b Institute of Chemistry & BioMedical Sciences , Collaborative Innovation Center of Chemistry for Life Sciences , Nanjing University , Nanjing 210093 , P. R. China; c Department of Chemistry and Biochemistry , Texas Tech University , Lubbock , TX 79409-1061 , USA . Email: guigen.li@ttu.edu

## Abstract

Copper-promoted direct carbonylation of unactivated sp^3^ C–H and aromatic sp^2^ C–H bonds of amides was developed using nitromethane as a novel carbonyl source. The sp^3^ C–H functionalization showed high site-selectivity by favoring the C–H bonds of α-methyl groups. The sp^2^ C–H carbonylation featured high regioselectivity and good functional group compatibility. Kinetic isotope effect studies indicated that the sp^3^ C–H bond breaking step is reversible, whereas the sp^2^ C–H bond cleavage is an irreversible but not the rate-determining step. Control experiments showed that a nitromethyl intermediate should be involved in the present reaction.

## Abstract 2

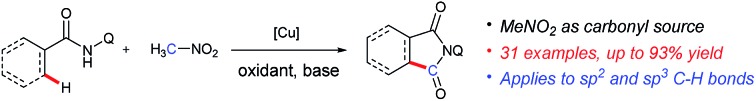
A copper-promoted site-selective carbonylation of sp^2^ and sp^3^ C–H bonds with nitromethane as an unprecedented source of carbonyl carbon is described.

## Introduction

**Scheme 1:**
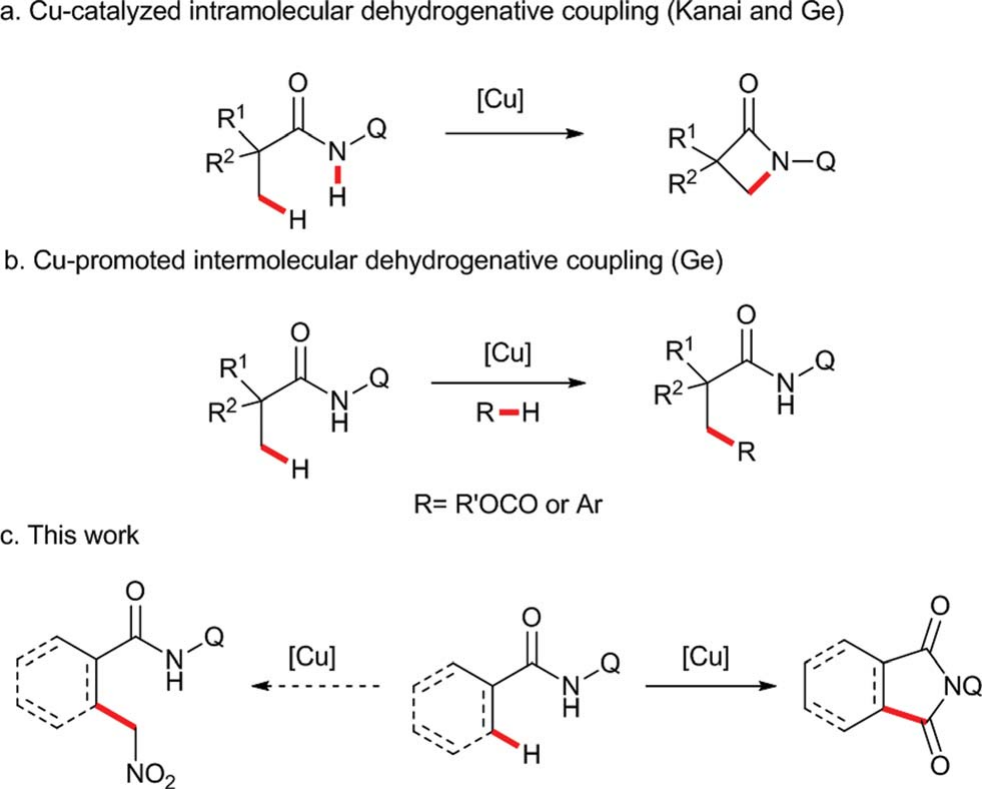
Copper-promoted dehydrogenative coupling of sp^3^ C–H bonds.

Transition metal-catalyzed direct C–H functionalization is one of the most convenient and efficient tools for selective C–C bond formation, and significant advances have been accomplished in this field during the past few years.^1^ Among the methods in this category, directing-group-assisted cross dehydrogenative coupling has attracted considerable attention due to its high regioselectivity and efficiency.^2^ In 2007, Miura and co-workers reported the first example of ligand-assisted regioselective copper-promoted cross dehydrogenative coupling of sp^2^ C–H bonds of 2-phenyl-pyridines and benzoxazoles.^3^ Following this pioneering study, a variety of nucleophiles and substrates were proven to be effective in this process.^4^ In these transformations, employing noble metals, such as palladium, rhodium, ruthenium or iridium, can be avoided, and therefore the reactions are more economical and synthetically useful than their counterparts. Recently, the copper-promoted direct functionalization of unactivated sp^3^ C–H bonds has also been achieved using bidentate directing groups. The intramolecular sp^3^ C–H amidation was developed by Kanai,^5^ You,^6^ and us^7^ independently (Scheme 1a). Subsequently, the copper-promoted cross dehydrogenative acyloxylation^8^ and arylation^9^ of unactivated sp^3^ C–H bonds were realized in our laboratory (Scheme 1b). However, the ligand directed copper-promoted dehydrogenative coupling of two sp^3^ C–H bonds remains a challenge.

Based on the abovementioned studies, we envisaged that the site-selective dehydrogenative coupling of an unactivated sp^3^ C–H bond and another reactive sp^3^ C–H bond species, such as nitromethane,^10^ alkylnitriles,^11^ or carbonyl compounds,^12^ could be performed by copper catalysis with bidentate directing group assistance. Therefore, we carried out the reaction of a series of aliphatic amides bearing the 8-aminoquinoline directing group with nitromethane in the presence of copper catalysts. To our surprise, an unexpected carbonylated compound was obtained instead of the dehydrogenative coupling product (Scheme 1c).^13^ Herein, we report this unprecedented β-carbonylation of amides with nitromethane as the carbonyl source *via* the copper-promoted C–H bond activation and a subsequent Nef type reaction.^14^

## Results and discussion

Our investigation commenced with 2-ethyl-2-methylpentanamide bearing a bidentate 8-aminoquinoline directing group (**1a**) as the model substrate (Table 1). Succinimide **2a** was initially obtained in 8% yield in the presence of Cu(OAc)_2_ and K_2_HPO_4_ at 165 °C under air (entry 2). Encouraged by this result, we examined different solvents and found that ^i^PrOH was a superior candidate (entry 6). Further investigation revealed that addition of an external single electron transfer oxidant can improve the yield, and K_2_S_2_O_8_ was proven to be the best pick (entries 9–11). Screening of bases showed that employing PhCO_2_Na as an additive, which was used in our previous report of intramolecular amidation, further increased the yield to 39% (entry 14). Mixed solvents were next surveyed, and a mixture of ^i^PrOH and dioxane led to a better yield (entry 15). Interestingly, the addition of Al_2_O_3_ and DMPU^15^ finally gave the best results for this dehydrogenative carbonylation reaction (entry 17). The control experiments showed that no desired product was observed in the absence of MeNO_2_ or the copper catalyst (entries 18 and 19).

**Table 1:** Optimization of the sp^3^ C–H carbonylation^*a*^

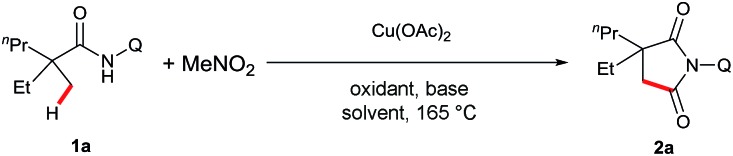				
Entry	Oxidant	Base	Solvent	Yield^*b*^ (%)
1		K_2_HPO_4_	MeNO_2_	0
2		K_2_HPO_4_	1,4-Dioxane	8
3		K_2_HPO_4_	MeCN	Trace
4		K_2_HPO_4_	^*t*^BuOH	11
5		K_2_HPO_4_	^*t*^AmOH	10
6		K_2_HPO_4_	^i^PrOH	14
7	O_2_	K_2_HPO_4_	^i^PrOH	Trace
8	AgOAc	K_2_HPO_4_	^i^PrOH	Trace
9	(*t*BuO)_2_	K_2_HPO_4_	^i^PrOH	18
10	Na_2_S_2_O_8_	K_2_HPO_4_	^i^PrOH	19
11	K_2_S_2_O_8_	K_2_HPO_4_	^i^PrOH	24
12	K_2_S_2_O_8_	Na_2_HPO_4_	^i^PrOH	26
13	K_2_S_2_O_8_	NaOAc	^i^PrOH	31
14	K_2_S_2_O_8_	PhCO_2_Na	^i^PrOH	39
15	K_2_S_2_O_8_	PhCO_2_Na	^i^PrOH/1,4-dioxane (0.45 : 0.55)	54
16^*c*^	K_2_S_2_O_8_	PhCO_2_Na	^i^PrOH/1,4-dioxane (0.45 : 0.55)	65
17^*c*^ ^,^^*d*^	K_2_S_2_O_8_	PhCO_2_Na	^i^PrOH/1,4-dioxane (0.45 : 0.55)	71(68)
18^*c*^ ^,^^*d*^ ^,^^*e*^	K_2_S_2_O_8_	PhCO_2_Na	^i^PrOH/1,4-dioxane (0.45 : 0.55)	0
19^*c*^ ^,^^*d*^ ^,^^*f*^	K_2_S_2_O_8_	PhCO_2_Na	^i^PrOH/1,4-dioxane (0.45 : 0.55)	0

^*a*^Reaction conditions: **1a** (0.3 mmol), Cu(OAc)_2_ (1 eq.), oxidant (2 eq.), base (1 eq.), solvent (2 mL), 165 °C, 24 h.

^*b*^Yields are based on **1a**, determined by ^1^H NMR using dibromomethane as the internal standard. Isolated yield is in parenthesis.

^*c*^Al_2_O_3_ (60 mg).

^*d*^DMPU (2 eq.).

^*e*^No MeNO_2_.

^*f*^No Cu(OAc)_2_.

**Scheme 2:**
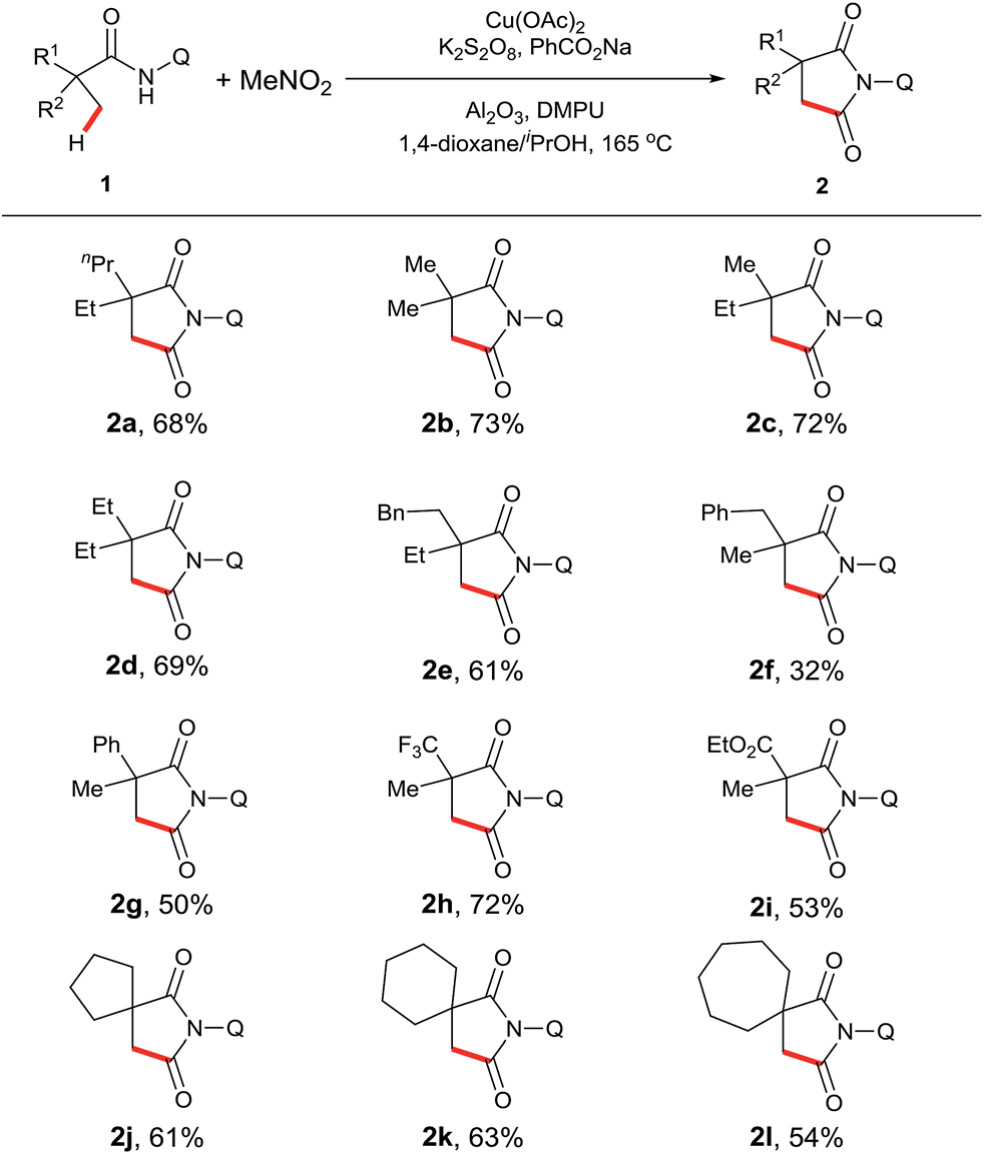
Scope of sp^3^ C–H carbonylation. Reaction conditions: **1** (0.3 mmol), Cu(OAc)_2_ (0.3 mmol), K_2_S_2_O_8_ (0.6 mmol), PhCO_2_Na (0.15 mmol), Al_2_O_3_ (60 mg), DMPU (0.6 mmol), MeNO_2_ (1.0 mL), 1,4-dioxane (0.9 mL), ^i^PrOH (1.1 mL), 165 °C, 24 h.

With the optimal conditions established, we examined the scope of aliphatic amide substrates (Scheme 2). Pivalamide proved to be an excellent substrate in this transformation, affording the carbonylation product in 73% yield (**2b**). Replacing the methyl group on the α-carbon with other alkyl groups, such as ethyl and propyl, gave the corresponding product in good yields (**2c** and **2d**). When the α-carbon was substituted with a benzyl group, the carbonylation occurred exclusively on the carbon center of the methyl group, presumably due to a steric effect (**2f**). α-Phenyl amide could participate in the reaction to readily provide the desired product (**2g**). Furthermore, substrates containing trifluoromethyl (**2h**) or methoxycarbonyl groups (**2i**) on the α-carbon proved to be viable. It is worth noting that the starting material was recovered with *N*-(quinolin-8-yl)isobutyramide as the substrate under the standard conditions, indicating that a quaternary α-carbon is required for this reaction. In addition, the removability of the quinolyl moiety was previously demonstrated in our laboratory.13*e*

To further expand the scope of the substrates and broaden the synthetic utility of this reaction, we next investigated the carbonylation of sp^2^ C–H bonds (Table 2). To our delight, the reaction could be realized with a catalytic amount of Cu(OAc)_2_. The optimal results were acquired with 2 equivalents Ag_2_CO_3_ and 1 equivalent PhCO_2_Na in DMA at 140 °C (entry 8).

**Table 2:** Optimization of the sp^2^ C–H carbonylation^*a*^

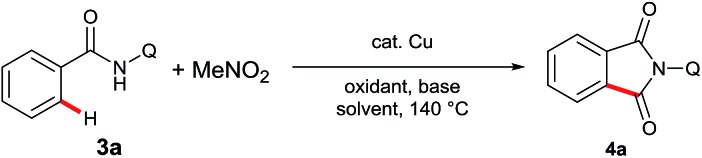					
Entry	Cu source	Oxidant	Base	Solvent	Yield^*b*^ (%)
1	Cu(OAc)_2_	O_2_		1,4-Dioxane	17
2	Cu(OAc)_2_	MnO_2_		1,4-Dioxane	28
3	Cu(OAc)_2_	NMO		1,4-Dioxane	33
4	Cu(OAc)_2_	Ag_2_O		1,4-Dioxane	19
5	Cu(OAc)_2_	Ag_2_CO_3_		1,4-Dioxane	45
6	Cu(OAc)_2_	Ag_2_CO_3_		DMA	74
7	Cu(OAc)_2_	Ag_2_CO_3_	PhCO_2_Na	DMA	69
8	Cu(OAc)_2_	Ag_2_CO_3_	Py	DMA	86
9	Cu(OAc)_2_	Ag_2_CO_3_	Na_2_HPO_4_	DMA	90(86)
10	CuCl	Ag_2_CO_3_	Na_2_HPO_4_	DMA	76
11	—	Ag_2_CO_3_	Na_2_HPO_4_	DMA	0

^*a*^Reaction conditions: **3a** (0.3 mmol), Cu(OAc)_2_ (10 mol%), oxidant (2 eq.), base (1 eq.), solvent (2 mL), 140 °C, 24 h.

^*b*^Yields are based on **3a**, determined by ^1^H NMR using dibromomethane as the internal standard. Isolated yield is in parenthesis.

**Scheme 3:**
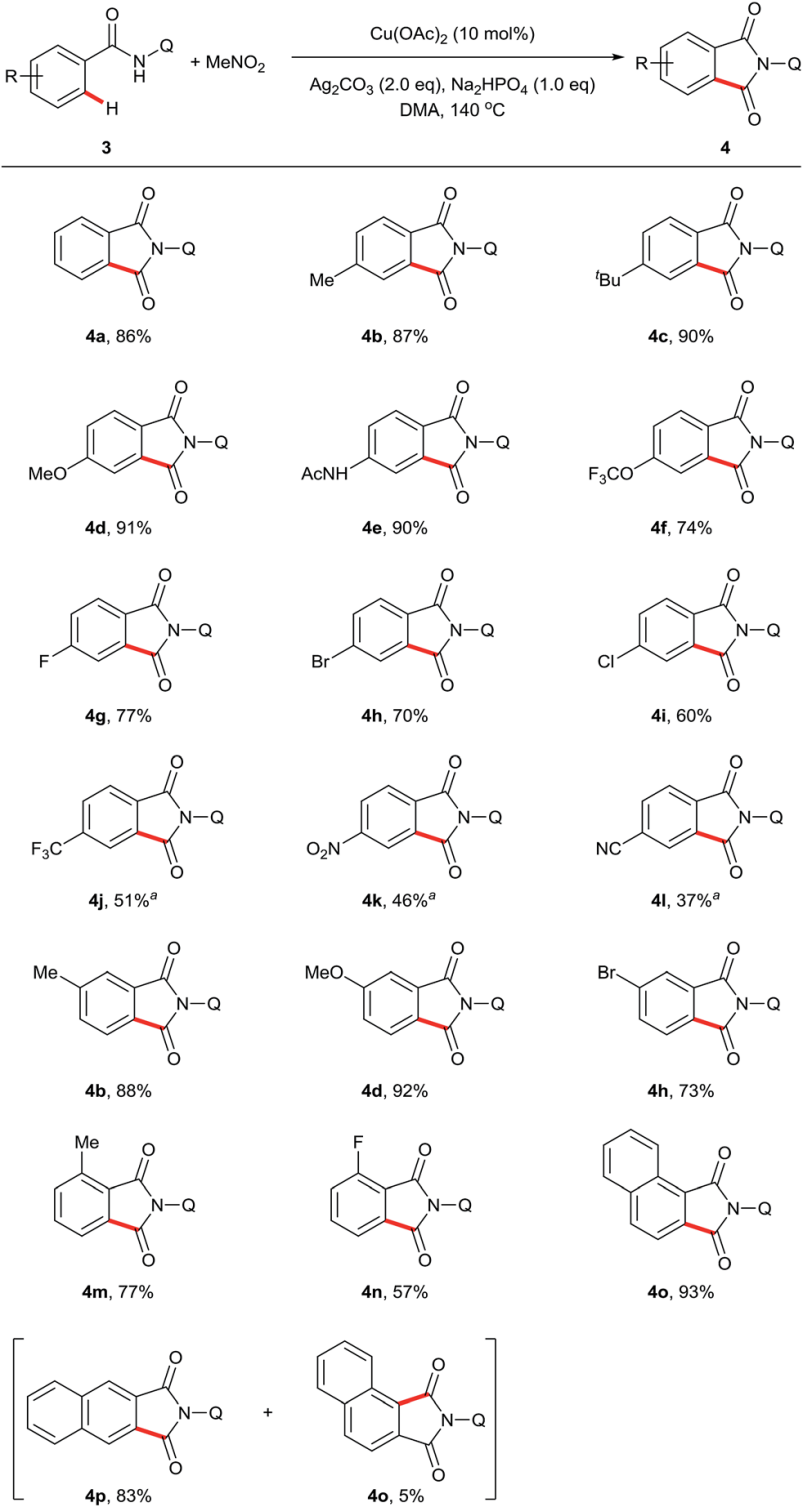
Scope of sp^2^ C–H carbonylation. Reaction conditions: **3** (0.3 mmol), Cu(OAc)_2_ (10 mol%), Ag_2_CO_3_ (2 eq.), Na_2_HPO_4_ (1 eq.), MeNO_2_ (0.5 mL), DMA (2 mL), 140 °C, 24 h. ^*a*^165 °C, 48 h.

Next, we examined the compatibility of the reaction with aromatic amide derivatives, which are summarized in Scheme 3. As expected, a wide range of functional groups including halogens were well tolerated under the optimized conditions. Substrates with electron-donating groups on the phenyl ring gave the desired products in good to excellent yields (**4d**, **4e**, and **4f**). Conversely, substrates containing halogen atoms afforded the phthalimides with slightly reduced yields (**4g**, **4h**, **4i**, and **4n**). Electron-withdrawing group substituted aromatic amides also provided the corresponding carbonylation products in moderate yields (**4j**, **4k**, and **4l**). Furthermore, 1-naphthamide and 2-naphthamide derivatives reacted to produce good yields (**4o** and **4p**).

**Scheme 4:**
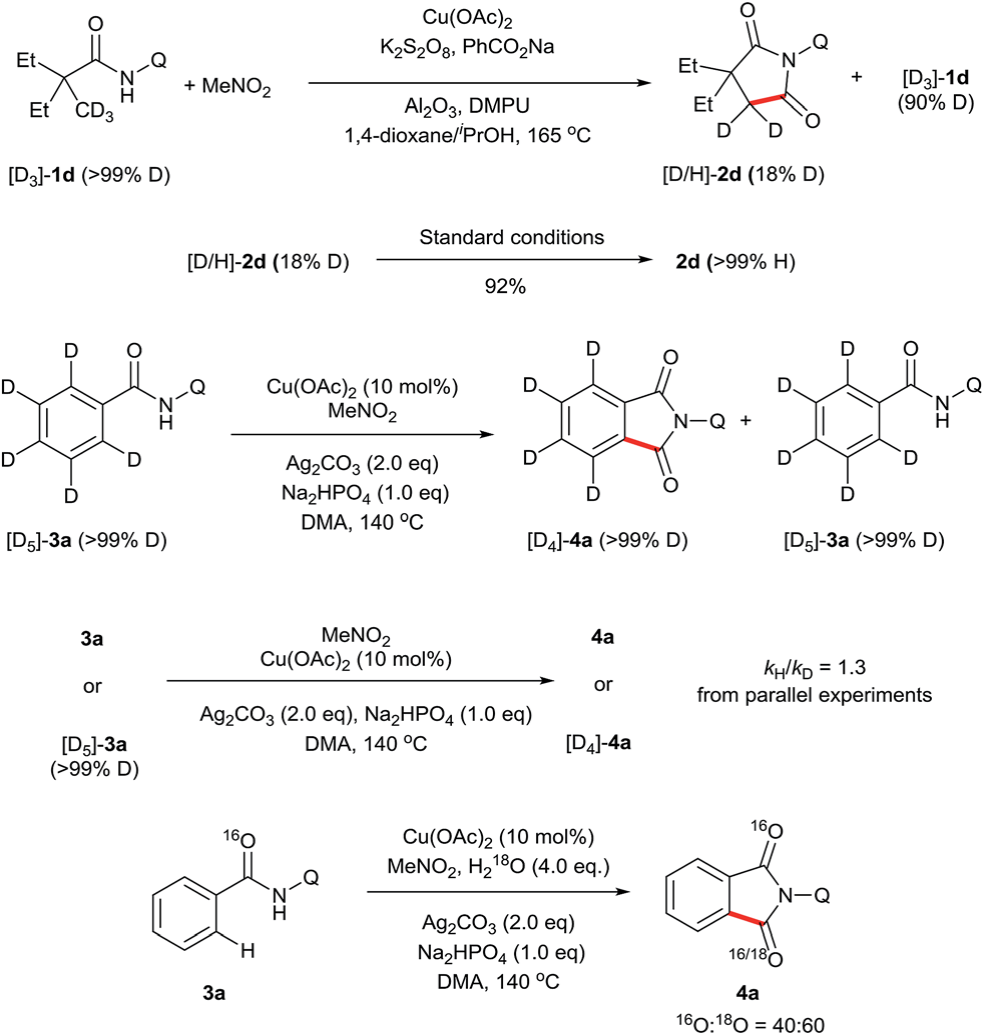
Kinetic isotope effect studies.

To gain some insights into this novel transformation mechanism, a series of deuterium-labelling experiments were performed. As shown in Scheme 4, evident H/D exchange of the substrate was found when the deuterium-labelled 2,2-diethyl-*N*-(quinolin-8-yl)pentanamide (D_3_-**1d**) was subjected to the standard conditions, indicating that the sp^3^ C–H bond cleavage is a reversible step. In addition, regular **2d** was obtained in 92% yield from the subjection of [D/H]-**2d** to the current reaction system, suggesting that the keto–enol tautomerism might account for the fast H/D exchange of the product [D/H]-**2d**. In contrast, no apparent H/D exchange was observed when the deuterium-labelled *N*-(quinolin-8-yl)benzamide (D_5_-**3a**) was subjected to the standard conditions, indicating that the sp^2^ C–H bond cleavage is an irreversible step. Furthermore, a secondary kinetic isotope effect was observed for **3a** based on the early relative rate of parallel reactions, indicating that the sp^2^ C–H cleavage of **3a** should not be the rate-determining step. Finally, the addition of 4 equivalents of H_2_^18^O to the reaction of **3a** resulted in 60% of ^18^O incorporation into **4a**, suggesting that water may be the source of oxygen in the carbonyl group.

**Scheme 5:**
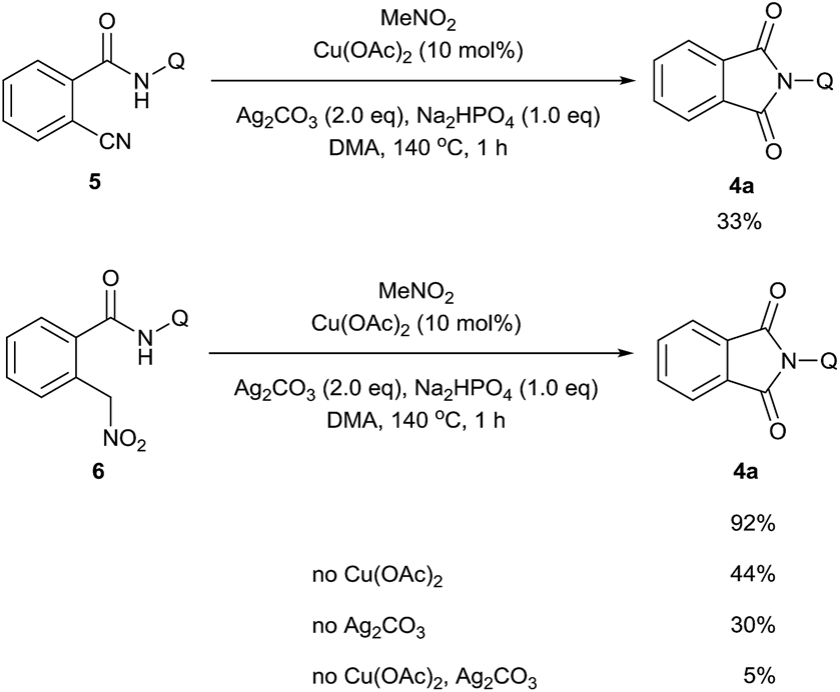
Control experiments.

A series of control experiments were carried out to further probe the transformation pathway (Scheme 5). The cyano compound **5**, a potential intermediate that was previously reported in the copper-catalyzed cyanation of 2-phenylpyridine with nitromethane10*b* was subjected to the reaction system and afforded the carbonylation product in 33% yield. On the other hand, the phthalimide product **4a** was obtained in 92% yield from the originally proposed nitromethyl product **6**, indicating that it is likely the major intermediate in this catalytic process. We then investigated the transformation from **6** to the product **4a** with a number of control experiments. It was found that either Cu(OAc)_2_ or Ag_2_CO_3_ could promote the reaction, whereas only a small amount of product was formed without any metal. We thus infer that the metal salts should act as Lewis acid catalysts in this process.

**Scheme 6:**
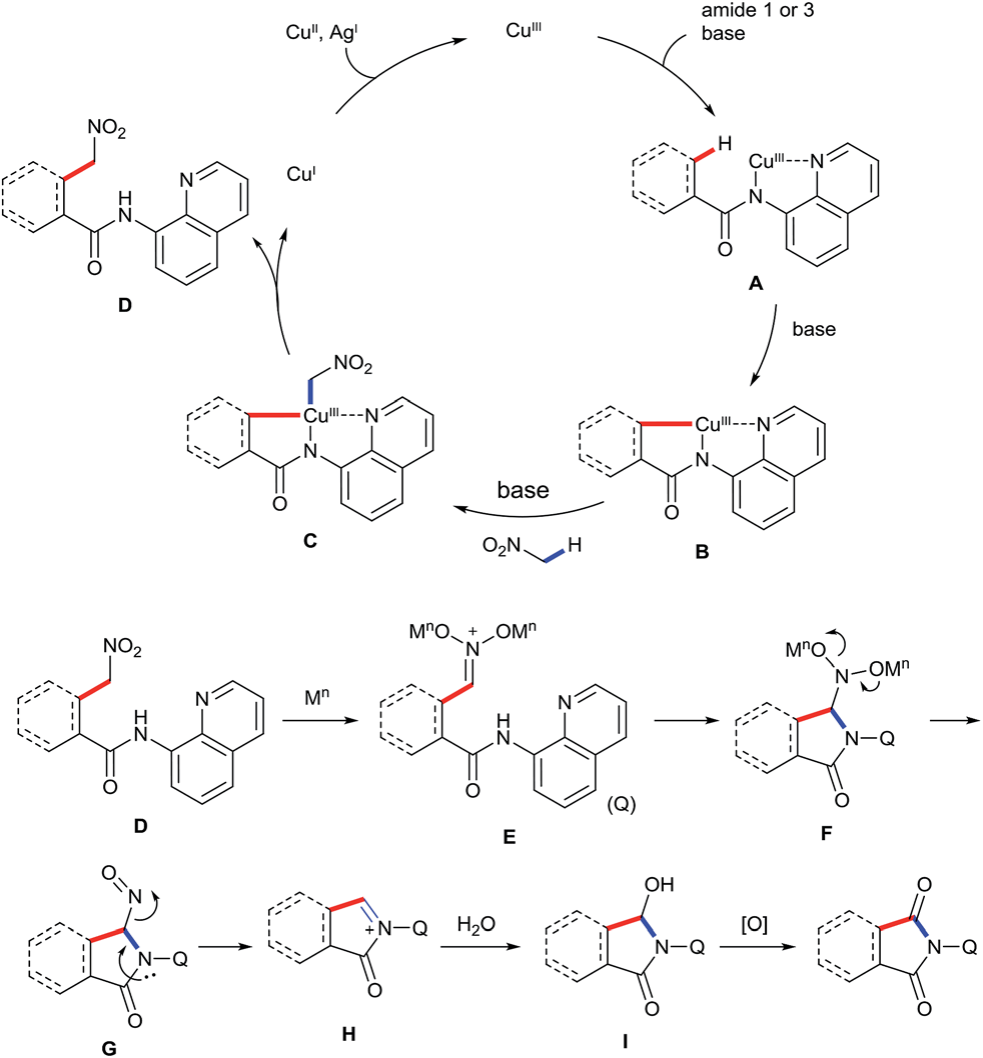
Plausible carbonylation reaction mechanism.

On the basis of the abovementioned results and previous reports,^5^–^9^,^16^ a plausible mechanism for the observed transformation is proposed and is depicted in Scheme 6. The reaction is believed to be initiated by coordinating the Cu^III^ species to the bidentate ligand, followed by ligand exchange under basic conditions to generate intermediated **A**. Cyclometalation of **A** through a sp^2^ or sp^3^ C–H activation process affords intermediate **B**. Subsequently, ligand exchange of **B** with nitromethane in the presence of the base affords intermediate **C**, which undergoes reductive elimination to give the intermediate **D**. Formation of iminium ion **E** in the presence of a Lewis acid, followed by a sequence of the intramolecular addition and the loss of the nitroso group gives rise to the imine intermediate **H**. Finally, the addition of water and the subsequent oxidation provide the desired product.

## Conclusions

In summary, a novel copper-promoted site-selective carbonylation of sp^2^ or unactivated sp^3^ C–H bonds has been established using nitromethane as the carbonyl source with the assistance of an 8-aminoquinolyl auxiliary. Preliminary mechanistic experiments suggested that the substrate undergoes a dehydrogenative coupling with nitromethane, followed by a Nef type reaction to form the carbonylation product. To the best of our knowledge, it is the first example of unactivated C–H bond functionalization integrated with the Nef reaction. Further studies toward understanding the detailed mechanism and potential application of this transformation are in process.

## Supplementary Material

Supplementary information
