# Supplementary material for: Copper-promoted site-selective carbonylation of sp3 and sp2 C–H bonds with nitromethane
Source: Chem Sci. 2016 Apr 26;7(8):5260–4. doi: 10.1039/c6sc01087c (PMC6020547; doi:10.1039/c6sc01087c)
Supplement: Supplementary file 1 [file SC-007-C6SC01087C-s001.pdf]

Electronic Supplementary Material (ESI) for Chemical Science.
This journal is © The Royal Society of Chemistry 2016

Copper-Promoted Site-Selective Carbonylation of sp^3 and sp^2 C–H Bonds with Nitromethane

Xuesong Wu, Jinmin Miao, Yanrong Li, Guigen Li* and Haibo Ge *

Table of Contents

General Information S2
Structures of Starting Materials S3
Procedure for the Preparation of Starting Materials (3e, 5 and 6) S5
General Procedure for Carbonylation of Aliphatic Amides S7
General Procedure for Carbonylation of Aromatic Amides S7
Analytical Data of Products S8
Deuterium Labeling Experiments S17
Parallel KIE Experiments S19
O^{18} Labeling Experiments S20
References S21
^1H, ^{13}C and ^{19}F NMR Spectra S22

General Information

^{1}H, ^{13}C and ^{19}F NMR spectra were recorded on a Bruker 500 MHz NMR Fourier transform spectrometer (500 MHz and 125 MHz, respectively) using tetramethylsilane as an internal reference, and chemical shifts (δ) and coupling constants (J) were expressed in ppm and Hz, respectively. Infrared spectra were obtained using a Thermo Nicolet IR 330 spectrometer. Mass (MS) analysis were abtained using Agilent 1100 series LC/MSD system with Electrospray Ionization (ESI). All the solvents and commercially available reagents were purchased from commercial sources and used directly. Starting materials **1a-l** and [D_3]-**1d** were prepared according to literature procedures.[1] **3a-d**, **3f-t** and [D_5]-**3a** were prepared based on reported reaction protocol.[2]

Structures of Starting Materials

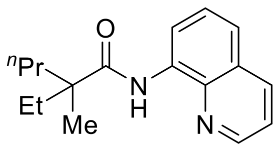

1a

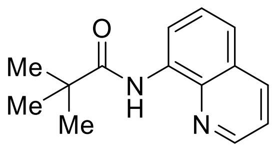

1b

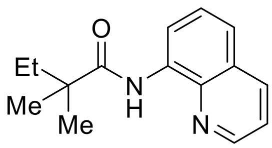

1c

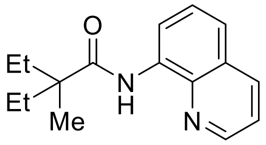

1d

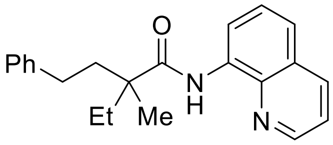

1e

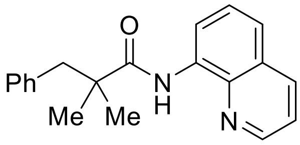

1f

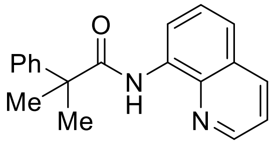

1g

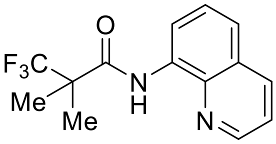

1h

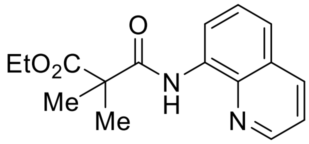

1i

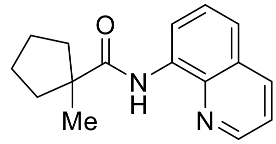

1j

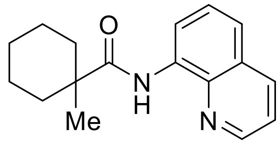

1k

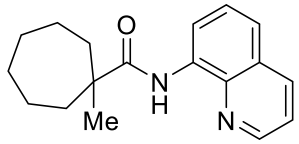

1l

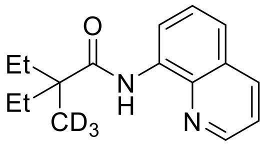

[D_3]-**1d**

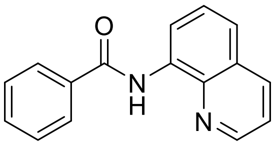

3a

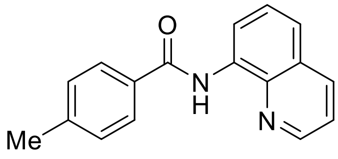

3b

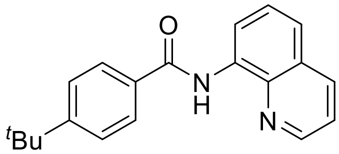

3c

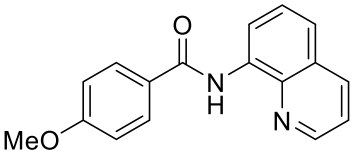

3d

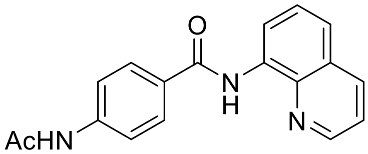

3e

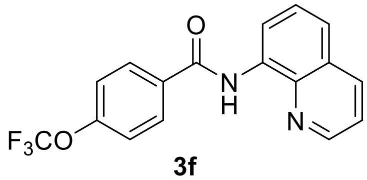

3f

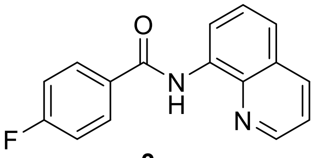

3g

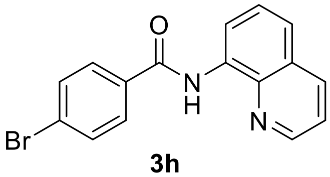

3h

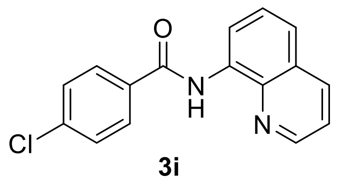
3i

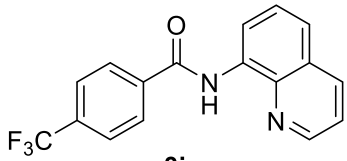
3j

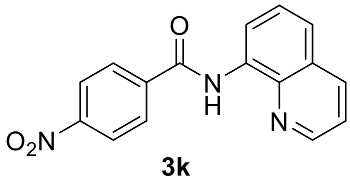
3k

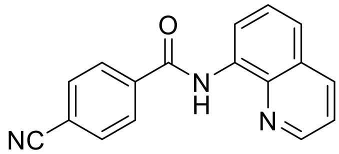
3l

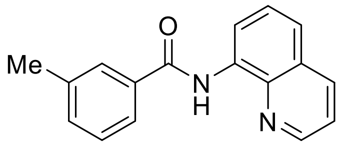
3m

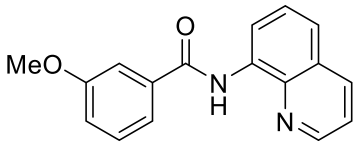
3n

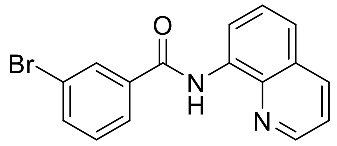
3o

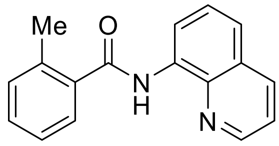
3p

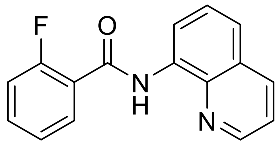
3q

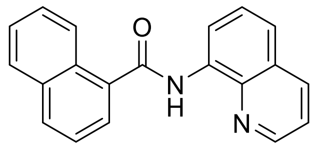
3r

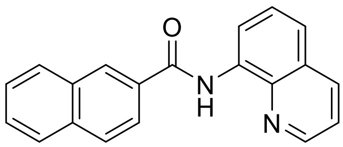
3s

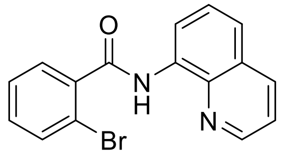
3t

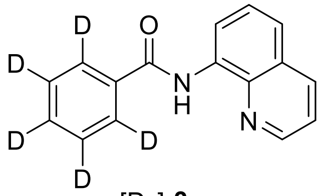
[D_5]-**3a**

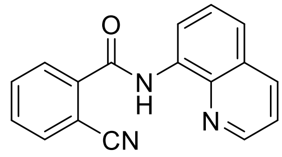
5

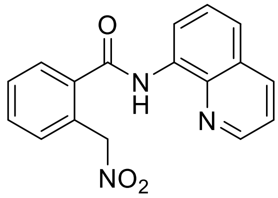
6

Procedure for the Preparation of Starting Materials (3e, 5 and 6)

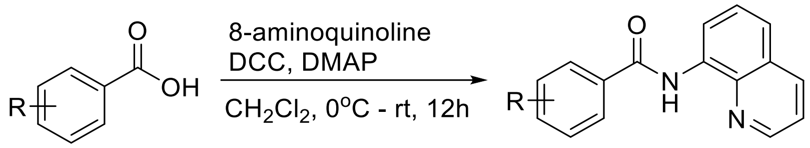

To a 100 mL flask was added benzoic acid derivative (5.0 mmol), 8-aminoquinoline (0.72 g, 5.0 mmol), DCC (1.20 g, 6.0 mmol), DMAP (0.20 g, 1.5 mmol) and dry CH_2Cl_2 (10 mL). The mixture was stirred at room temperature for 12h. Then the reaction mixture was diluted by CH_2Cl_2 (40 mL) and filtered. The filtrate was concentrated under reduced pressure. The residue was purified by flash column chromatography on silica gel, eluting with EtOAc/Hexane (1:20 ~ 1:10, v/v), to afford corresponding amide.

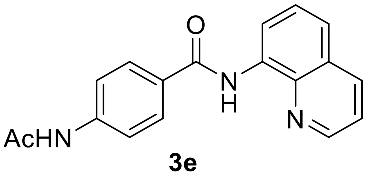

3e

Compound **3e**, white solid. ^{1}H NMR (500 MHz, $CDCl_3$) δ 10.73 (s, 1H), 8.91 (dd, J = 7.5, 1.4 Hz, 1H), 8.85 (dd, J = 4.2, 1.7 Hz, 1H), 8.18 (dd, J = 8.3, 1.6 Hz, 1H), 8.06 (d, J = 8.7 Hz, 2H), 7.71 (d, J = 8.5 Hz, 2H), 7.62 – 7.51 (m, 3H), 7.48 (dd, J = 8.2, 4.2 Hz, 1H), 2.23 (s, 3H); ^{13}C NMR (125 MHz, $CDCl_3$) δ 168.6, 165.0, 148.5, 141.4, 138.9, 136.5, 134.7, 130.7, 128.6, 128.2, 127.6, 121.9, 121.8, 119.5, 116.6, 24.9; IR (neat) v 3459, 3334, 3262, 3177, 3098, 1712, 1635, 1540, 1489, 1324, 1279, 843, 824, 790, 760, 675; Ms (ESI): m/z = 306.1 $[M+H]^+$.

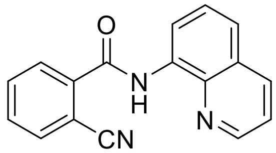

5

Compound **5**, white solid. ^{1}H NMR (500 MHz, $CDCl_3$) δ 10.62 (s, 1H), 8.95 (dd, J = 6.3, 2.7 Hz, 1H), 8.82 (dd, J = 4.2, 1.7 Hz, 1H), 8.20 (dd, J = 8.3, 1.6 Hz, 1H), 8.02 (dd, J = 7.8, 0.7 Hz, 1H), 7.87 (dd, J = 7.7, 1.0 Hz, 1H), 7.79 – 7.75 (m, 1H), 7.68 – 7.64 (m, 1H), 7.63 – 7.57 (m, 2H), 7.48 (dd, J = 8.3, 4.2 Hz, 1H); ^{13}C NMR (125 MHz, $CDCl_3$) δ 163.3, 148.6, 139.2, 138.8, 136.6, 135.0, 134.1, 132.9, 131.5, 128.2, 128.1, 127.5, 122.6, 122.0, 117.6, 117.4, 111.9; IR (neat) v 3336, 3052, 2927,

2852, 2227, 1734, 1662, 1531, 1480, 1328, 1122, 905, 826, 791, 760, 705; Ms (ESI): m/z = 274.1 $[M+H]^+$.

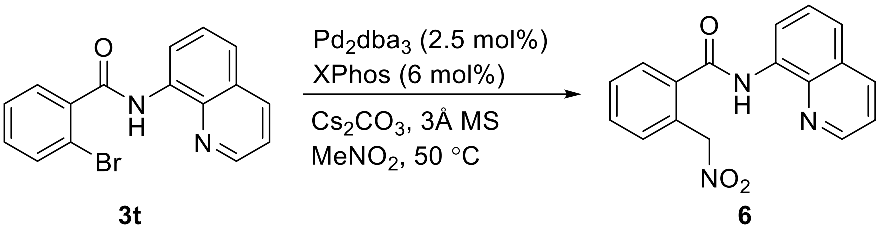

A 100 mL Schlenk tube was charged with Cs_2CO_3 (716 mg, 2.2 mmol), 3 Å molecular sieves (400 mg), Pd_2dba_3 (45.6 mg, 0.050 mmol), and XPhos (57.2 mg, 0.12 mmol). Nitromethane (20.0 mL) was added, followed by addition of compound **3t** (654 mg, 2.0 mmol). The tube was evacuated and filled with N_2, and stirred rigorously at 50 °C for 24h. The reaction mixture was allowed to cool to rt, diluted with CH_2Cl_2 (40 mL), and washed with saturated aq. NH_4Cl (2 x 20 mL). The aqueous layer was extracted with CH_2Cl_2 (2 x 30 mL). The combined organic layers were washed with brine, dried over Na_2SO_4, filtered, and concentrated in vacuo. The residue was purified by flash column chromatography on silica gel, eluting with EtOAc/Hexane (1:10, v/v), to afford compound **6** (434 mg, 69 % yield).[3]

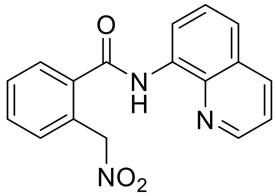

6

Compound **6**, yellow oil. ^{1}H NMR (500 MHz, $CDCl_3$) δ 10.53 (s, 1H), 8.87 (dd, J = 6.1, 2.9 Hz, 1H), 8.82 (dd, J = 4.2, 1.7 Hz, 1H), 8.20 (dd, J = 8.3, 1.7 Hz, 1H), 7.95 (dd, J = 7.4, 1.5 Hz, 1H), 7.67 – 7.57 (m, 4H), 7.53 – 7.46 (m, 2H), 5.94 (s, 2H); ^{13}C NMR (125 MHz, $CDCl_3$) δ 166.4, 148.6, 138.9, 136.8, 136.6, 134.5, 133.0, 131.5, 130.6, 129.4, 128.2, 127.9, 127.5, 122.4, 122.0, 117.0, 76.9; IR (neat) ν 3340, 3060, 2921, 1669, 1557, 1526, 1482, 1373, 1327, 1267, 901, 826, 792, 699; Ms (ESI): m/z = 308.1 $[M+H]^+$.

General Procedure for Carbonylation of Aliphatic Amides

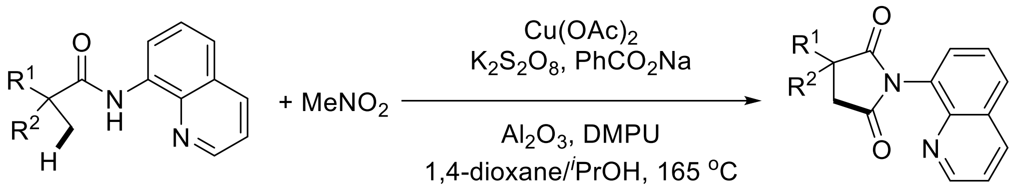

1 **2**

A 50 mL Schlenk tube was charged with amide (**1**, 0.30 mmol), $Cu(OAc)_2$ (54.5 mg, 0.30 mmol), $K_2S_2O_8$ (162 mg, 0.60 mmol), $PhCO_2Na$ (21.6 mg, 0.15 mmol), neutral alumina (60 mg), DMPU (72 μL, 77 mg, 0.60 mmol), 1,4-dioxane (0.9 mL), isopropanol (1.1 mL) and nitromethane (1.0 mL). The tube was capped and stirred rigorously at 165 °C for 24h. Then the reaction mixture was cooled to room temperature, diluted with EtOAc (20 mL) and filtered. The filtrate was washed with water (10 mL), brine (10 mL), dried over anhydrous sodium sulfate, and concentrated under reduced pressure. The residue was purified by flash column chromatography on silica gel, eluting with EtOAc/Hexane (1:4 ~ 1:1, v/v), to afford corresponding product **2**.

General Procedure for Carbonylation of Aromatic Amides

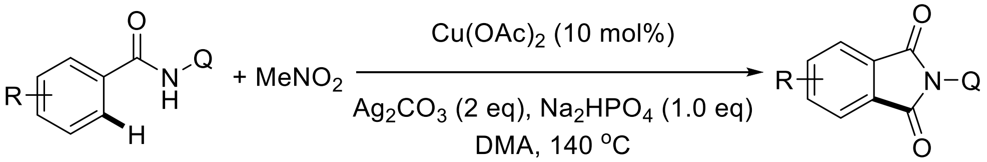

3 **4**

A 50 mL Schlenk tube was charged with amide (**3**, 0.30 mmol), $Cu(OAc)_2$ (5.4 mg, 0.030 mmol), Ag_2CO_3 (165 mg, 0.60 mmol), Na_2HPO_4 (42.6 mg, 0.30 mmol), DMA (2.0 mL) and nitromethane (0.5 mL). The tube was capped and stirred rigorously at 140 °C for 24h. Then the reaction mixture was cooled to room temperature, diluted with EtOAc (30 mL) and filtered. The filtrate was washed with water (3 × 10 mL), brine (10 mL), dried over anhydrous sodium sulfate, and concentrated under reduced pressure. The residue was purified by flash column chromatography on silica gel, eluting with EtOAc/Hexane (1:4 ~ 1:1, v/v), to afford corresponding product **4**.

Analytical Data of Products

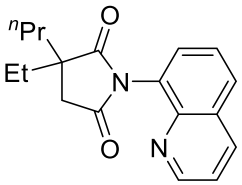

2a

Compound **2a**, colorless oil, yield: 68%. (This compound is known.[4]) ^{1}H NMR (500 MHz, $CDCl_3$, a mixture of atropisomers in ration 1.0:1.0, the minor one is marked with an *) δ 8.86 – 8.83 (m, 1H), 8.18 – 8.15 (m, 1H), 7.90 (dd, J = 8.0, 1.6 Hz, 1H), 7.63 – 7.56 (m, 2H), 7.43 – 7.39 (m, 1H), 2.95 (dd, J = 18.4, 1.1 Hz, 1H), 2.76 (dd, J = 18.4, 2.4 Hz, 1H), 2.05 – 1.35 (m, 6H), 1.16 (t, J = 7.5 Hz, 3H), 1.07 – 0.99 (m, 3H); 8.86 – 8.83 (m, 1H)*, 8.18 – 8.15 (m, 1H)*, 7.90 (dd, J = 8.0, 1.6 Hz, 1H)*, 7.63 – 7.56 (m, 2H)*, 7.43 – 7.39 (m, 1H)*, 2.95 (dd, J = 18.4, 1.1 Hz, 1H)*, 2.76 (dd, J = 18.4, 2.4 Hz, 1H)*, 2.05 – 1.35 (m, 6H)*, 1.07 – 0.99 (m, 6H)*; Ms (ESI): m/z = 297.1 $[M+H]^+$.

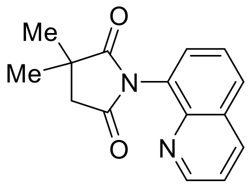

2b

Compound **2b**, colorless oil, yield: 73%. (This compound is known.[4]) ^{1}H NMR (500 MHz, $CDCl_3$) δ 8.86 (dd, J = 4.2, 1.7 Hz, 1H), 8.18 (dd, J = 8.3, 1.7 Hz, 1H), 7.95-7.89 (m, 1H), 7.65 – 7.60 (m, 2H), 7.42 (dd, J = 8.3, 4.2 Hz, 1H), 2.95 (d, J = 18.0 Hz, 1H), 2.82 (d, J = 18.0 Hz, 1H), 1.61 (s, 3H), 1.50 (s, 3H)); Ms (ESI): m/z = 255.1 $[M+H]^+$.

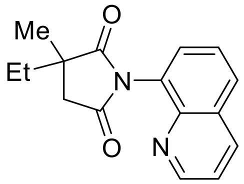

2c

Compound **2c**, colorless oil, yield: 72%. (This compound is known.[4]) ^{1}H NMR (500 MHz, $CDCl_3$, a mixture of atropisomers in ration 1.1:1.0, the minor one is marked with an *) δ 8.86 – 8.82 (m, 1H), 8.18 – 8.14 (m, 1H), 7.92 – 7.87 (m, 1H), 7.63 – 7.58 (m, 2H), 7.42 – 7.38 (m, 1H), 2.86 (dd, J = 31.0, 18.2 Hz, 2H), 2.06 – 1.88 (m, 1H), 1.86 – 1.71 (m, 1H), 1.58 (s, 3H), 1.06 (t, J = 7.5 Hz, 3H); 8.87 – 8.83 (m, 1H)*, 8.18 – 8.14 (m, 1H)*, 7.92 – 7.87 (m, 1H)*, 7.63 – 7.58 (m, 2H)*, 7.42 – 7.38 (m,

1H)*, 3.03 (d, J = 18.2 Hz, 1H)*, 2.68 (d, J = 18.2 Hz, 1H)*, 2.06 – 1.88 (m, 1H)*, 1.86 – 1.71 (m, 1H)*, 1.47 (s, 3H)*, 1.17 (t, J = 7.5 Hz, 3H)*; Ms (ESI): m/z = 269.1 $[M+H]^+$.

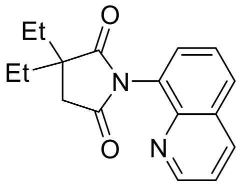

2d

Compound **2d**, colorless oil, yield: 69%. (This compound is known.[4]) ^{1}H NMR (500 MHz, $CDCl_3$) δ 8.84 (dd, J = 4.1, 1.7 Hz, 1H), 8.16 (dd, J = 8.3, 1.7 Hz, 1H), 7.90 (dd, J = 7.9, 1.7 Hz, 1H), 7.64 – 7.56 (m, 2H), 7.40 (dd, J = 8.3, 4.2 Hz, 1H), 2.94 (d, J = 18.4 Hz, 1H), 2.75 (d, J = 18.4 Hz, 1H), 2.05 – 1.87 (m, 2H), 1.86 – 1.73 (m, 2H), 1.17 (t, J = 7.5 Hz, 3H), 1.05 (t, J = 7.4 Hz, 3H); Ms (ESI): m/z = 283.1 $[M+H]^+$.

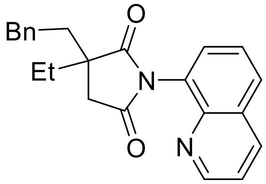

2e

Compound **2e**, white solid, yield: 61%. (This compound is known.[4]) ^{1}H NMR (500 MHz, $CDCl_3$, a mixture of atropisomers in ration 1.4:1.0, the minor one is marked with an *) δ 8.81 (dd, J = 4.2, 1.7 Hz, 1H), 8.16 (dd, J = 8.2, 1.4 Hz, 1H), 7.92 – 7.87 (m, 1H), 7.65 – 7.55 (m, 2H), 7.42 – 7.37 (m, 1H), 7.33 – 7.18 (m, 5H), 3.05 – 2.69 (m, 4H), 2.26 – 1.78 (m, 4H), 1.07 (t, J = 7.5 Hz, 3H); 8.84 (dd, J = 4.2, 1.7 Hz, 1H)*, 8.16 (dd, J = 8.2, 1.4 Hz, 1H)*, 7.92 – 7.87 (m, 1H)*, 7.65 – 7.55 (m, 2H)*, 7.42 – 7.37 (m, 1H)*, 7.33 – 7.18 (m, 5H)*, 3.05 – 2.69 (m, 4H)*, 2.26 – 1.78 (m, 4H)*, 1.19 (t, J = 7.5 Hz, 3H)*; Ms (ESI): m/z = 359.1 $[M+H]^+$.

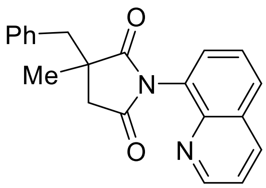

2f

Compound **2f**, yellow oil, yield: 32%. (This compound is known.[4]) ^{1}H NMR (500 MHz, $CDCl_3$, a mixture of atropisomers in ration 2.3:1.0, the minor one is marked with an *) δ 8.85 (dd, J = 4.2, 1.7 Hz, 1H), 8.15 – 8.12 (m, 1H), 7.88 (dd, J = 8.3, 1.3 Hz, 1H), 7.55 – 7.51 (m, 1H), 7.41 – 7.23 (m, 6H), 7.16 (dd, J = 7.3, 1.4 Hz, 1H), 3.34 (d, J = 13.3 Hz, 1H), 3.05 – 2.98 (m, 1H), 2.83 – 2.75 (m, 2H), 1.71 (s, 3H); 8.79 (dd, J = 4.2, 1.7 Hz, 1H)*, 8.17 – 8.14 (m, 1H)*, 7.92 – 7.88 (m, 1H)*, 7.62 – 7.59 (m, 2H)*, 7.41 – 7.23 (m, 6H)*, 3.41 (d, J = 13.7 Hz, 1H)*, 3.18 (d, J = 18.1 Hz, 1H)*, 3.05 – 3.00 (m, 1H)*, 2.57 (d, J = 18.1 Hz, 1H)*, 1.53 (s, 3H)*; Ms (ESI): m/z = 331.1 $[M+H]^+$.

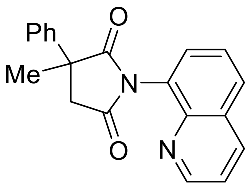

2g

Compound **2g**, white solid, yield: 50%. (This compound is known.[4]) ^{1}H NMR (500 MHz, $CDCl_3$, a mixture of atropisomers in ration 1.1:1.0, the minor one is marked with an *) δ 8.91 – 8.89 (m, 1H), 8.21 (dd, J = 8.3, 1.7 Hz, 1H), 7.97 – 7.92 (m, 1H), 7.80 – 7.75 (m, 1H), 7.71 – 7.62 (m, 2H), 7.58 – 7.53 (m, 1H), 7.48 – 7.40 (m, 3H), 7.36 – 7.31 (m, 1H), 3.39 (d, J = 18.2, 1H), 3.12 (d, J = 18.4 Hz, 1H), 2.01 (s, 3H); 8.91 – 8.89 (m, 1H)*, 8.21 (dd, J = 8.3, 1.7 Hz, 1H)*, 7.97 – 7.92 (m, 1H)*, 7.80 – 7.75 (m, 1H)*, 7.71 – 7.62 (m, 2H)*, 7.58 – 7.53 (m, 1H)*, 7.48 – 7.40 (m, 3H)*, 7.36 – 7.31 (m, 1H)*, 3.37 (d, J = 18.3 Hz, 1H)*, 3.26 (d, J = 18.1 Hz, 1H)*, 1.93 (s, 3H)*; Ms (ESI): m/z = 317.1 $[M+H]^+$.

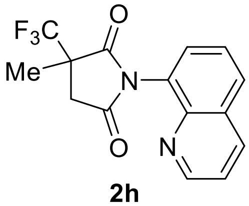

2h

Compound **2h**, colorless oil, yield: 72%. (This compound is known.[4]) ^{1}H NMR (500 MHz, $CDCl_3$, a mixture of atropisomers in ration 2.9:1.0, the minor one is marked with an *) δ 8.84 (dd, J = 4.2, 1.6 Hz, 1H), 8.22 – 8.18 (m, 1H), 7.97 – 7.91 (m, 1H), 7.66 – 7.59 (m, 2H), 7.46 – 7.41 (m, 1H), 3.32 (d, J = 18.4 Hz, 1H), 2.99 (d, J = 18.4 Hz, 1H), 1.86 (s, 3H); 8.87 (dd, J = 4.2, 1.6 Hz, 1H)*, 8.19 – 8.16 (m, 1H)*, 7.97 – 7.91 (m, 2H)*, 7.66 – 7.59 (m, 1H)*, 7.46 – 7.41 (m, 1H)*, 3.47 (d, J = 18.4 Hz,

1H)*, 2.84 (d, J = 18.4 Hz, 1H)*, 1.75 (s, 3H)*; ^{19}F NMR (471 MHz, $CDCl_3$) δ -74.3, -74.6; Ms (ESI): m/z = 309.1 $[M+H]^+$.

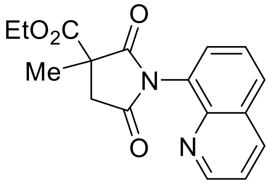

2i

Compound **2i**, yellow oil, yield: 53%. (This compound is known.[4]) ^{1}H NMR (500 MHz, $CDCl_3$, a mixture of atropisomers in ration 2.5:1.0, the minor one is marked with an *) δ 8.90 – 8.84 (m, 1H), 8.25 – 8.19 (m, 1H), 7.97 – 7.91 (m, 1H), 7.68 – 7.60 (m, 2H), 7.47 – 7.42 (m, 1H), 4.32 (q, J = 7.1 Hz, 2H), 3.42 (d, J = 18.1 Hz, 1H), 2.97 (d, J = 18.0 Hz, 1H), 1.84 (s, 3H), 1.35 (t, J = 7.1 Hz, 3H); 8.90 – 8.84 (m, 1H)*, 8.25 – 8.19 (m, 1H)*, 7.97 – 7.91 (m, 1H)*, 7.68 – 7.60 (m, 2H)*, 7.47 – 7.42 (m, 1H)*, 4.32 (q, J = 7.1 Hz, 2H)*, 3.66 (d, J = 18.0 Hz, 1H)*, 2.81 (d, J = 18.0 Hz, 1H)*, 1.76 (s, 3H)*, 1.35 (t, J = 7.1 Hz, 3H)*; Ms (ESI): m/z = 313.1 $[M+H]^+$.

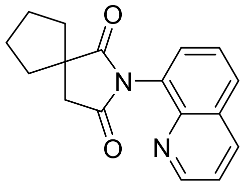

2j

Compound **2j**, colorless oil, yield: 61%. (This compound is known.[4]) ^{1}H NMR (500 MHz, $CDCl_3$) δ 8.86 (dd, J = 4.2, 1.7 Hz, 1H), 8.17 (dd, J = 8.3, 1.7 Hz, 1H), 7.93 – 7.88 (m, 1H), 7.65 – 7.59 (m, 2H), 7.41 (dd, J = 8.3, 4.2 Hz, 1H), 2.99 (d, J = 18.0 Hz, 1H), 2.85 (d, J = 18.0 Hz, 1H), 2.47 – 2.38 (m, 1H), 2.34 – 2.26 (m, 1H), 2.03 – 1.93 (m, 3H), 1.88 – 1.78 (m, 3H); Ms (ESI): m/z = 281.1 $[M+H]^+$.

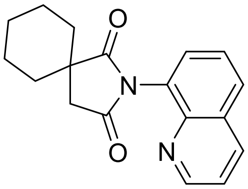

2k

Compound **2k**, colorless oil, yield: 63%. (This compound is known.[4]) ^{1}H NMR (500 MHz, $CDCl_3$) δ 8.84 (dd, J = 4.2, 1.7 Hz, 1H), 8.17 (dd, J = 8.3, 1.6 Hz, 1H), 7.93 – 7.88 (m, 1H), 7.64 – 7.59 (m, 2H), 7.41 (dd, J = 8.3, 4.2 Hz, 1H), 2.98 (d, J = 18.1 Hz, 1H), 2.80 (d, J = 18.1 Hz, 1H), 2.04 – 1.95 (m, 3H), 1.91 – 1.84 (m, 2H), 1.79 – 1.71 (m, 2H), 1.50 – 1.38 (m, 3H); Ms (ESI): m/z = 295.1 $[M+H]^+$.

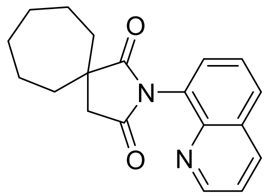

2l

Compound **2l**, colorless oil, yield: 54%. (This compound is known.[4]) ^{1}H NMR (500 MHz, $CDCl_3$) δ 8.85 (dd, J = 4.2, 1.7 Hz, 1H), 8.17 (dd, J = 8.3, 1.7 Hz, 1H), 7.93 – 7.88 (m, 1H), 7.64 – 7.58 (m, 2H), 7.42 (dd, J = 8.3, 4.2 Hz, 1H), 2.96 (d, J = 18.0 Hz, 1H), 2.81 (d, J = 18.0 Hz, 1H), 2.29 – 2.22 (m, 1H), 2.21 – 2.15 (m, 1H), 2.11 – 2.05 (m, 1H), 1.95 – 1.82 (m, 3H), 1.70 – 1.53 (m, 6H); Ms (ESI): m/z = 309.2 $[M+H]^+$.

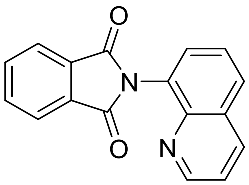

4a

Compound **4a**, white solid, yield: 86%. (This compound is known.[5]) ^{1}H NMR (500 MHz, $CDCl_3$) δ 8.85 (dd, J = 4.2, 1.7 Hz, 1H), 8.22 (dd, J = 8.3, 1.7 Hz, 1H), 8.02 – 7.93 (m, 3H), 7.83 – 7.78 (m, 2H), 7.75 (dd, J = 7.3, 1.4 Hz, 1H), 7.69 – 7.64 (m, 1H), 7.43 (dd, J = 8.3, 4.2 Hz, 1H); Ms (ESI): m/z = 275.1 $[M+H]^+$.

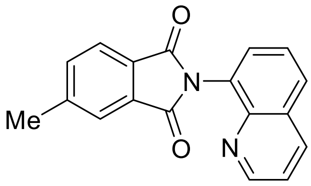

4b

Compound **4b**, white solid, yield: 87%. (This compound is known.[5]) ^{1}H NMR (500 MHz, $CDCl_3$) δ 8.84 (dd, J = 4.2, 1.7 Hz, 1H), 8.21 (dd, J = 8.3, 1.7 Hz, 1H), 7.94 (dd, J = 8.2, 1.4 Hz, 1H), 7.87 (d, J = 7.6 Hz, 1H), 7.80 (s, 1H), 7.74 (dd, J = 7.3, 1.4 Hz, 1H), 7.68 – 7.63 (m, 1H), 7.60 – 7.56 (m, 1H), 7.42 (dd, J = 8.3, 4.2 Hz, 1H), 2.55 (s, 3H); Ms (ESI): m/z = 289.1 $[M+H]^+$.

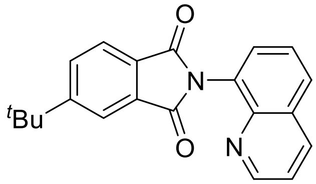

4c

Compound **4c**, white solid, yield: 90%. ^{1}H NMR (500 MHz, $CDCl_3$) δ 8.83 (dd, J = 4.2, 1.7 Hz, 1H), 8.19 (dd, J = 8.3, 1.7 Hz, 1H), 8.02 (d, J = 1.3 Hz, 1H), 7.95 – 7.87 (m, 2H), 7.81 (dd, J = 7.9, 1.7 Hz, 1H), 7.72 (dd, J = 7.3, 1.4 Hz, 1H), 7.67 – 7.62 (m, 1H), 7.40 (dd, J = 8.3, 4.2 Hz, 1H), 1.40 (s, 9H); ^{13}C NMR (125 MHz, $CDCl_3$) δ 168.4, 168.1, 158.9, 151.0, 144.4, 136.3, 132.7, 131.3, 130.4, 130.1, 129.9, 129.6, 129.4, 126.2, 123.7, 121.9, 121.1, 35.9, 31.3; IR (neat) ν 2964, 2870, 1779, 1720, 1643, 1501, 1396, 1237, 1103, 885, 825, 792, 747, 691; Ms (ESI): m/z = 331.1 $[M+H]^+$.

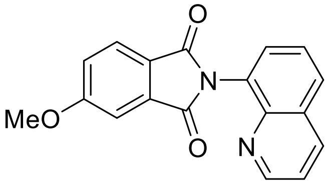

4d

Compound **4d**, white solid, yield: 91%. (This compound is known.[5]) ^{1}H NMR (500 MHz, $CDCl_3$) δ 8.85 (d, J = 2.8 Hz, 1H), 8.21 (dd, J = 8.3, 1.6 Hz, 1H), 7.94 (dd, J = 8.2, 1.0 Hz, 1H), 7.88 (d, J = 8.3 Hz, 1H), 7.74 (dd, J = 7.3, 1.4 Hz, 1H), 7.66 (dd, J = 8.1, 7.4 Hz, 1H), 7.46 (d, J = 2.3 Hz, 1H), 7.42 (dd, J = 8.3, 4.2 Hz, 1H), 7.25 (dd, J = 8.3, 2.3 Hz, 1H), 3.95 (s, 3H); Ms (ESI): m/z = 305.1 $[M+H]^+$.

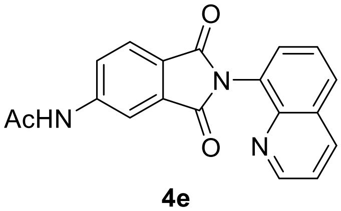

4e

Compound **4e**, white solid, yield: 90%. ^{1}H NMR (500 MHz, $CDCl_3$) δ 9.34 (s, 1H), 8.85 (dd, J = 4.2, 1.6 Hz, 1H), 8.24 (dd, J = 8.3, 1.6 Hz, 1H), 8.01 – 7.88 (m, 3H), 7.75 (dd, J = 10.9, 4.9 Hz, 2H), 7.66 (dd, J = 8.1, 7.4 Hz, 1H), 7.45 (dd, J = 8.3, 4.2 Hz, 1H), 1.92 (s, 3H); ^{13}C NMR (125 MHz, $CDCl_3$) δ 169.5, 168.0, 167.8, 150.9, 144.6, 144.2, 136.9, 133.6, 130.8, 129.9, 129.7, 129.5, 126.6, 126.3, 125.0, 124.1, 122.2, 114.3, 24.3; IR (neat) ν 3283, 3151, 3085, 2928, 2856, 2251, 1772, 1700, 1645, 1521, 1395, 1279, 1106, 912, 885, 818, 789, 745, 626; Ms (ESI): m/z = 332.1 $[M+H]^+$.

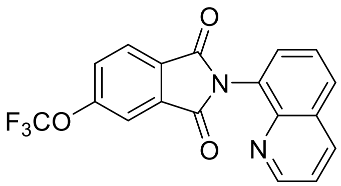

4f

Compound **4f**, white solid, yield: 74%. ^{1}H NMR (500 MHz, $CDCl_3$) δ 8.85 (dd, J = 4.2, 1.7 Hz, 1H), 8.23 (dd, J = 8.3, 1.7 Hz, 1H), 8.06 – 8.02 (m, 1H), 7.97 (dd, J = 8.2, 1.4 Hz, 1H), 7.86 – 7.81 (m, 1H), 7.75 (dd, J = 7.3, 1.4 Hz, 1H), 7.68 (dd, J = 8.2, 7.3 Hz, 1H), 7.63 – 7.60 (m, 1H), 7.45 (dd, J = 8.3, 4.2 Hz, 1H); ^{13}C NMR (125 MHz, $CDCl_3$) δ 166.7, 166.5, 153.8 (q, J_{COCF} = 1.8 Hz), 151.1, 144.2, 136.4, 134.9, 130.4, 130.3, 123.0, 129.6, 129.5, 126.4, 126.3, 125.9, 122.2, 120.4 (q, J_{CF} = 260.0 Hz), 116.2; ^{19}F NMR (471 MHz, $CDCl_3$) δ -57.7; IR (neat) ν 3071, 2925, 2854, 1785, 1726, 1616, 1501, 1398, 1213, 1175, 912, 884, 831, 744; Ms (ESI): m/z = 359.1 $[M+H]^+$.

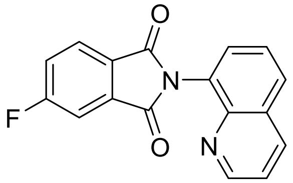

4g

Compound **4g**, white solid, yield: 77%. (This compound is known.[4]) ^{1}H NMR (500 MHz, $CDCl_3$) δ 8.85 (dd, J = 4.2, 1.7 Hz, 1H), 8.23 (dd, J = 8.3, 1.7 Hz, 1H), 8.02 – 7.96 (m, 2H), 7.75 (dd, J = 7.3, 1.4 Hz, 1H), 7.71 – 7.65 (m, 2H), 7.51 – 7.42 (m, 2H); ^{19}F NMR (471 MHz, $CDCl_3$) δ -101.7; Ms (ESI): m/z = 293.1 $[M+H]^+$.

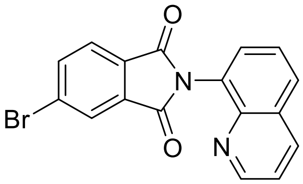

4h

Compound **4h**, pale yellow solid, yield: 70%. (This compound is known.[5]) ^{1}H NMR (500 MHz, $CDCl_3$) δ 8.85 (dd, J = 4.2, 1.7 Hz, 1H), 8.23 (dd, J = 8.3, 1.7 Hz, 1H), 8.14 (d, J = 1.4 Hz, 1H), 7.99 – 7.93 (m, 2H), 7.86 (dd, J = 7.9, 0.4 Hz, 1H), 7.74 (dd, J = 7.3, 1.5 Hz, 1H), 7.68 (dd, J = 8.1, 7.4 Hz, 1H), 7.45 (dd, J = 8.3, 4.2 Hz, 1H); Ms (ESI): m/z = 353.0 $[M+H]^+$.

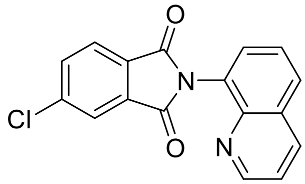

4i

Compound **4i**, white solid, yield: 60%. (This compound is known.[4]) ^{1}H NMR (500 MHz, $CDCl_3$) δ 8.84 (dd, J = 4.2, 1.7 Hz, 1H), 8.23 (dd, J = 8.3, 1.7 Hz, 1H), 7.99 – 7.96 (m, 2H), 7.94 (dd, J = 8.0, 0.5 Hz, 1H), 7.79 – 7.73 (m, 1H), 7.68 (dd, J = 8.1, 7.4 Hz, 1H), 7.45 (dd, J = 8.3, 4.2 Hz, 1H); Ms (ESI): m/z = 309.0 $[M+H]^+$.

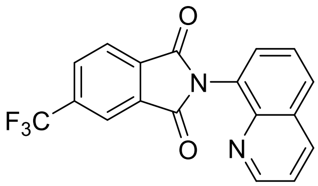

4j

Compound **4j**, white solid, yield: 51%. (This compound is known.[5]) ^{1}H NMR (500 MHz, $CDCl_3$) δ 8.84 (dd, J = 4.2, 1.7 Hz, 1H), 8.28 (s, 1H), 8.25 (dd, J = 8.3, 1.7 Hz, 1H), 8.14 (d, J = 7.8 Hz, 1H), 8.10 (d, J = 7.8 Hz, 1H), 8.00 (dd, J = 8.2, 1.4 Hz, 1H), 7.77 (dd, J = 7.3, 1.4 Hz, 1H), 7.72 – 7.68 (m, 1H), 7.47 (dd, J = 8.3, 4.2 Hz, 1H); ^{19}F NMR (471 MHz, $CDCl_3$) δ -62.9; Ms (ESI): m/z = 343.1 $[M+H]^+$.

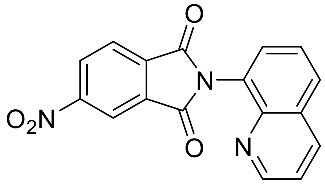

4k

Compound **4k**, yellow solid, yield: 46%. (This compound is known.[5]) ^{1}H NMR (500 MHz, $CDCl_3$) δ 8.86 – 8.80 (m, 2H), 8.70 (dd, J = 8.1, 2.0 Hz, 1H), 8.26 (dd, J =

8.3, 1.7 Hz, 1H), 8.20 (d, J = 8.2 Hz, 1H), 8.01 (dd, J = 8.2, 1.4 Hz, 1H), 7.78 (dd, J = 7.3, 1.4 Hz, 1H), 7.73 – 7.69 (m, 1H), 7.48 (dd, J = 8.3, 4.2 Hz, 1H); Ms (ESI): m/z = 320.1 $[M+H]^+$.

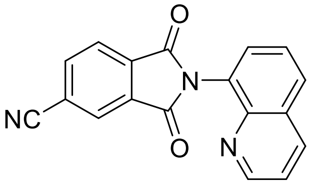

4l

Compound **4l**, pale yellow solid, yield: 37%. (This compound is known.[5]) ^{1}H NMR (500 MHz, $CDCl_3$) δ 8.83 (dd, J = 4.2, 1.7 Hz, 1H), 8.30 – 8.23 (m, 2H), 8.15 – 8.09 (m, 2H), 8.01 (dd, J = 8.2, 1.4 Hz, 1H), 7.76 (dd, J = 7.3, 1.4 Hz, 1H), 7.72 – 7.68 (m, 1H), 7.47 (dd, J = 8.3, 4.2 Hz, 1H); Ms (ESI): m/z = 320.1 $[M+H]^+$.

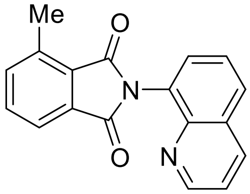

4m

Compound **4m**, white solid, yield: 77%. (This compound is known.[5]) ^{1}H NMR (500 MHz, $CDCl_3$) δ 8.87 (dd, J = 4.2, 1.7 Hz, 1H), 8.23 (dd, J = 8.3, 1.7 Hz, 1H), 7.96 (dd, J = 8.2, 1.4 Hz, 1H), 7.83 (d, J = 7.3 Hz, 1H), 7.74 (dd, J = 7.3, 1.4 Hz, 1H), 7.68 – 7.62 (m, 2H), 7.55 (d, J = 7.7 Hz, 1H), 7.44 (dd, J = 8.3, 4.2 Hz, 1H), 2.76 (s, 3H); Ms (ESI): m/z = 289.1 $[M+H]^+$.

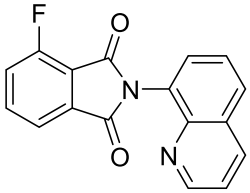

4n

Compound **4n**, white solid, yield: 57%. ^{1}H NMR (500 MHz, $CDCl_3$) δ 8.86 (dd, J = 4.2, 1.7 Hz, 1H), 8.24 (dd, J = 8.3, 1.7 Hz, 1H), 7.98 (dd, J = 8.2, 1.4 Hz, 1H), 7.87 – 7.77 (m, 2H), 7.75 (dd, J = 7.3, 1.4 Hz, 1H), 7.71 – 7.65 (m, 1H), 7.49 – 7.43 (m,

2H); ^{13}C NMR (125 MHz, $CDCl_3$) δ 166.9 (d, J_{CCCF} = 2.9 Hz), 164.6 (d, J_{CCCCF} = 1.4 Hz), 158.1 (d, J_{CF} = 266.2 Hz), 151.2, 144.44, 136.8 (d, J_{CCCF} = 7.6 Hz), 136.4, 134.8, 130.4, 123.0, 129.6, 129.5, 126.3, 122.7 (d, J_{CCF} = 19.7 Hz), 122.1, 120.2 (d, J_{CCCF} = 3.7 Hz), 118.5 (d, J_{CCF} = 12.4 Hz); ^{19}F NMR (471 MHz, $CDCl_3$) δ -112.2; IR (neat) *v* 2925, 2854, 1781, 1721, 1611, 1501, 1481, 1397, 1258, 1112, 961, 911, 880, 826, 790, 744; Ms (ESI): *m*/*z* = 293.1 $[M+H]^+$.

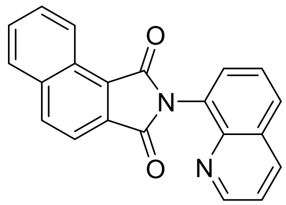

4o

Compound **4o**, white solid, yield: 93%. (This compound is known.[4]) ^{1}H NMR (500 MHz, $CDCl_3$) δ 9.04 – 9.00 (m, 1H), 8.86 (dd, *J* = 4.2, 1.7 Hz, 1H), 8.27 – 8.22 (m, 2H), 8.02 – 7.96 (m, 3H), 7.81 (dd, *J* = 7.3, 1.4 Hz, 1H), 7.75 – 7.67 (m, 3H), 7.44 (dd, *J* = 8.3, 4.2 Hz, 1H); Ms (ESI): *m*/*z* = 325.1 $[M+H]^+$.

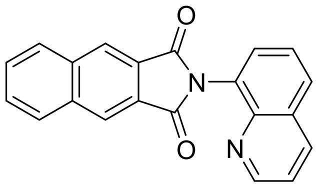

4p

Compound **4p**, yellow solid, yield: 83%. (This compound is known.[5]) ^{1}H NMR (500 MHz, $CDCl_3$) δ 8.86 (dd, *J* = 4.2, 1.7 Hz, 1H), 8.50 (s, 2H), 8.23 (dd, *J* = 8.3, 1.7 Hz, 1H), 8.14 – 8.08 (m, 2H), 7.98 (dd, *J* = 8.3, 1.3 Hz, 1H), 7.81 (dd, *J* = 7.3, 1.4 Hz, 1H), 7.75 – 7.67 (m, 3H), 7.44 (dd, *J* = 8.3, 4.2 Hz, 1H); Ms (ESI): *m*/*z* = 325.1 $[M+H]^+$.

Deuterium Labeling Experiments

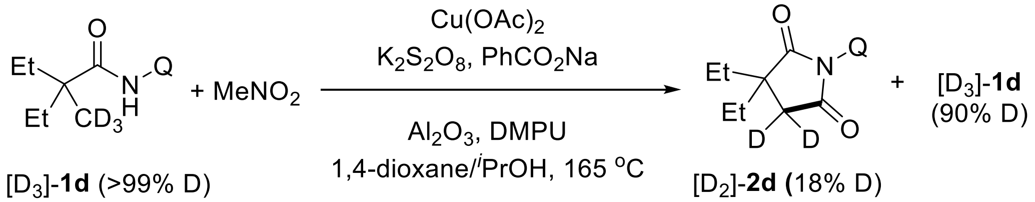

A 50 mL Schlenk tube was charged with [D_3]-**1d** (77.7 mg, 0.30 mmol), $Cu(OAc)_2$ (54.5 mg, 0.30 mmol), $K_2S_2O_8$ (162 mg, 0.60 mmol), $PhCO_2Na$ (21.6 mg, 0.15 mmol), neutral alumina (60 mg), DMPU (72 μL, 77 mg, 0.60 mmol), 1,4-dioxane (0.9 mL), isopropanol (1.1 mL) and nitromethane (1.0 mL). The tube was capped and stirred rigorously at 165 °C for 4h. Then the reaction mixture was cooled to room temperature, diluted with EtOAc (20 mL) and filtered. The filtrate was washed with water (10 mL), brine (10 mL), dried over anhydrous sodium sulfate, and concentrated under reduced pressure. The residue was purified by flash column chromatography on silica gel, eluting with EtOAc/Hexane (1:4 ~ 1:1, v/v), to afford product [D_2]-**2d** (16.2 mg, 19%) and recovered [D_3]-**1d** (57.5 mg, 74%). The ratio of deuterium was determined by ^{1}H NMR.

[D_2]-**2d**, ^{1}H NMR (500 MHz, $CDCl_3$) δ 8.85 (dd, J = 4.2, 1.7 Hz, 1H), 8.18 (dd, J = 8.3, 1.7 Hz, 1H), 7.92 (dd, J = 8.1, 1.5 Hz, 1H), 7.64 – 7.59 (m, 1H), 7.57 (dd, J = 7.3, 1.5 Hz, 1H), 7.42 (dd, J = 8.3, 4.2 Hz, 1H), 2.94 (d, J = 18.4 Hz, **0.82H**), 2.75 (d, J = 18.4 Hz, **0.81H**), 2.04 – 1.95 (m, 1H), 1.94 – 1.87 (m, 2H), 1.86 – 1.72 (m, 2H), 1.17 (t, J = 7.5 Hz, 3H), 1.06 (t, J = 7.5 Hz, 3H).

Recovered [D_3]-**1d**, ^{1}H NMR (500 MHz, $CDCl_3$) δ 10.22 (s, 1H), 8.87 – 8.77 (m, 2H), 8.13 (dd, J = 8.3, 1.6 Hz, 1H), 7.56 – 7.39 (m, 3H), 1.93 – 1.83 (m, 2H), 1.67 – 1.58 (m, 2H), 1.37 – 1.29 (m, **0.31H**), 0.94 (t, J = 7.5 Hz, 6H).

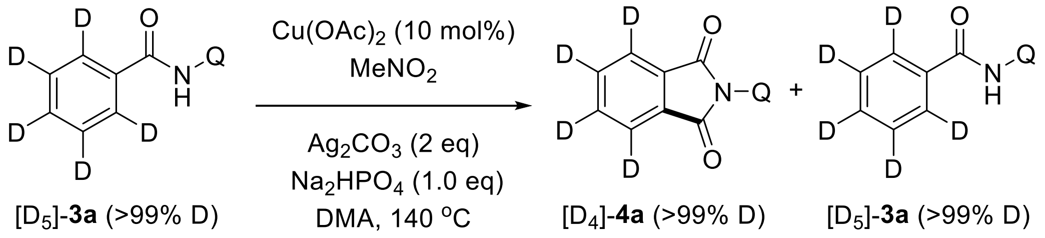

A 50 mL Schlenk tube was charged with [D_5]-**3a** (75.9 mg, 0.30 mmol), $Cu(OAc)_2$ (5.4 mg, 0.030 mmol), Ag_2CO_3 (165 mg, 0.60 mmol), Na_2HPO_4 (42.6 mg, 0.30 mmol), DMA (2.0 mL) and nitromethane (0.5 mL). The tube was capped and stirred rigorously at 140 °C for 4h. Then the reaction mixture was cooled to room temperature, diluted with EtOAc (30 mL) and filtered. The filtrate was washed with water (3 × 10 mL), brine (10 mL), dried over anhydrous sodium sulfate, and concentrated under reduced pressure. The residue was purified by flash column chromatography on silica gel, eluting with EtOAc/Hexane (1:4 ~ 1:1, v/v), to afford product [D_4]-**4a** (20.0 mg, 24%) and recovered [D_5]-**3a** (54.6 mg, 72%). The ratio of deuterium was determined by ^{1}H NMR.

$[D_4]$-**4a**, ^{1}H NMR (500 MHz, $CDCl_3$) δ 8.88 (dd, J = 4.2, 1.7 Hz, 1H), 8.23 (dd, J = 8.3, 1.7 Hz, 1H), 7.97 (dd, J = 8.2, 1.4 Hz, 1H), 7.76 (dd, J = 7.3, 1.4 Hz, 1H), 7.68 (dd, J = 8.2, 7.3 Hz, 1H), 7.45 (dd, J = 8.3, 4.2 Hz, 1H).

Recovered $[D_5]$-**3a**, ^{1}H NMR (500 MHz, $CDCl_3$) δ 10.75 (s, 1H), 8.95 (dd, J = 7.6, 1.3 Hz, 1H), 8.84 (dd, J = 4.2, 1.7 Hz, 1H), 8.17 (dd, J = 8.3, 1.6 Hz, 1H), 7.59 (t, J = 7.9 Hz, 1H), 7.54 (dd, J = 8.3, 1.3 Hz, 1H), 7.47 (dd, J = 8.2, 4.2 Hz, 1H).

Parallel KIE Experiments

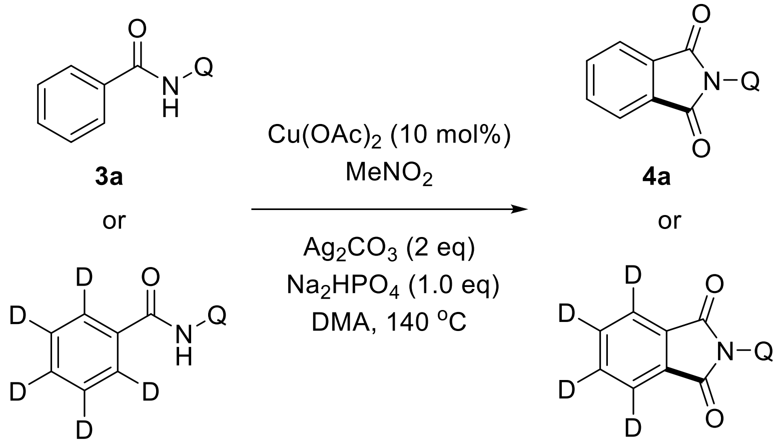

$[D_5]$-**3a** (>99% D) $[D_4]$-**4a** (>99% D)

A 50 mL Schlenk tube was charged with **3a** (74.4 mg, 0.30 mmol) or $[D_5]$-**3a** (75.9 mg, 0.30 mmol), $Cu(OAc)_2$ (5.4 mg, 0.030 mmol), Ag_2CO_3 (165 mg, 0.60 mmol), Na_2HPO_4 (42.6 mg, 0.30 mmol), DMA (2.0 mL) and nitromethane (0.5 mL). The tube was capped and stirred rigorously at 140 °C. The reaction was stopped by rapid cooling in the indicated reaction period, and analyzed by GC using benzophenone as the internal standard. The average GC yield was calculated after calibrating the response of GC based on three runs of each reaction.

Time (min)	5	10	15	20	25	30	35	40	45	50	55	60	70	80	90
Yield of **4a** (%)	0.4	1.1	2.0	2.1	3.9	4.2	3.6	5.0	5.5	7.5	7.4	8.2	9.2	12.4	14.1
Yield of $[D_4]$-**4a** (%)	0.2	0.7	1.1	2.0	2.3	5.0	4.1	4.6	4.9	5.7	5.9	7.6	8.0	8.3	11.2

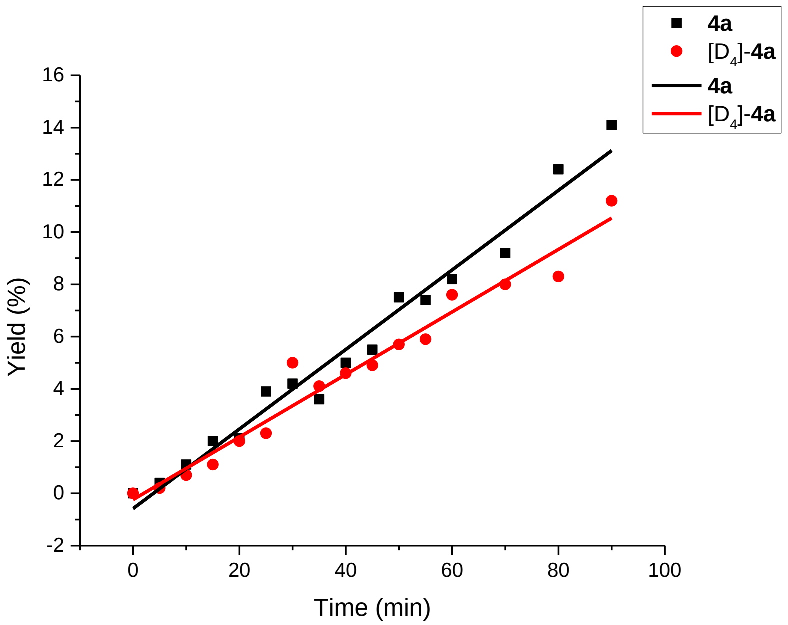

Equation for **4a**: y = 0.152x – 0.583 R^2 = 0.974

Equation for [D_4]-**4a**: y = 0.120x + 0.286 R^2 = 0.963

k_H/k_D = 0.152/0.120 ≈ 1.3

KIE value determined from parallel reactions is 1.3.

O^{18} Labeling Experiments

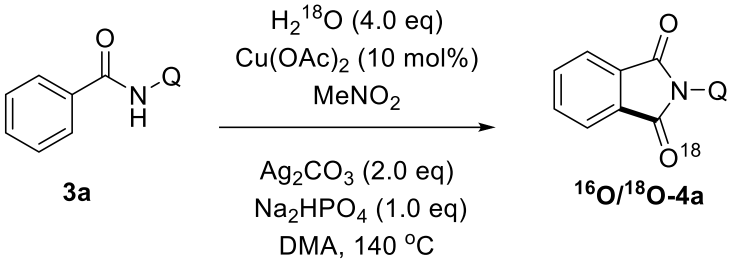

A 50 mL Schlenk tube was charged with amide (**3a**, 74.5 mg, 0.30 mmol), $Cu(OAc)_2$ (5.4 mg, 0.030 mmol), Ag_2CO_3 (165 mg, 0.60 mmol), Na_2HPO_4 (42.6 mg, 0.30 mmol), DMA (2.0 mL) and nitromethane (0.5 mL). $H_2{}^{18}O$ (97 atom % ^{18}O, 22 µL, 24 mg, 1.2 mmol) was added into the reaction mixture. The tube was capped and stirred rigorously at 140 °C for 24h. Then the reaction mixture was cooled to room temperature, diluted with EtOAc (30 mL) and filtered. The filtrate was washed with water (3 × 10 mL), brine (10 mL), dried over anhydrous sodium sulfate, and concentrated under reduced pressure. The residue was purified by flash column

chromatography on silica gel, eluting with EtOAc/Hexane (1:4 ~ 1:1, v/v), to afford the ^{18}O incorporated product $^{16}O/^{18}O$-**4a**. The product was analyzed by HRMS.

ESI-MS of $[C_{17}H_{17}N_2O_2]^+$; theoretical m/z of $[M+H]^+$ = 275.0815, measured m/z of $[M+H]^+$ = 275.0823.

ESI-MS of $[C_{17}H_{17}N_2O^{18}O]^+$; theoretical m/z of $[M+H]^+$ = 277.0857, measured m/z of $[M+H]^+$ = 277.0863. 60% isotopic enhancement.

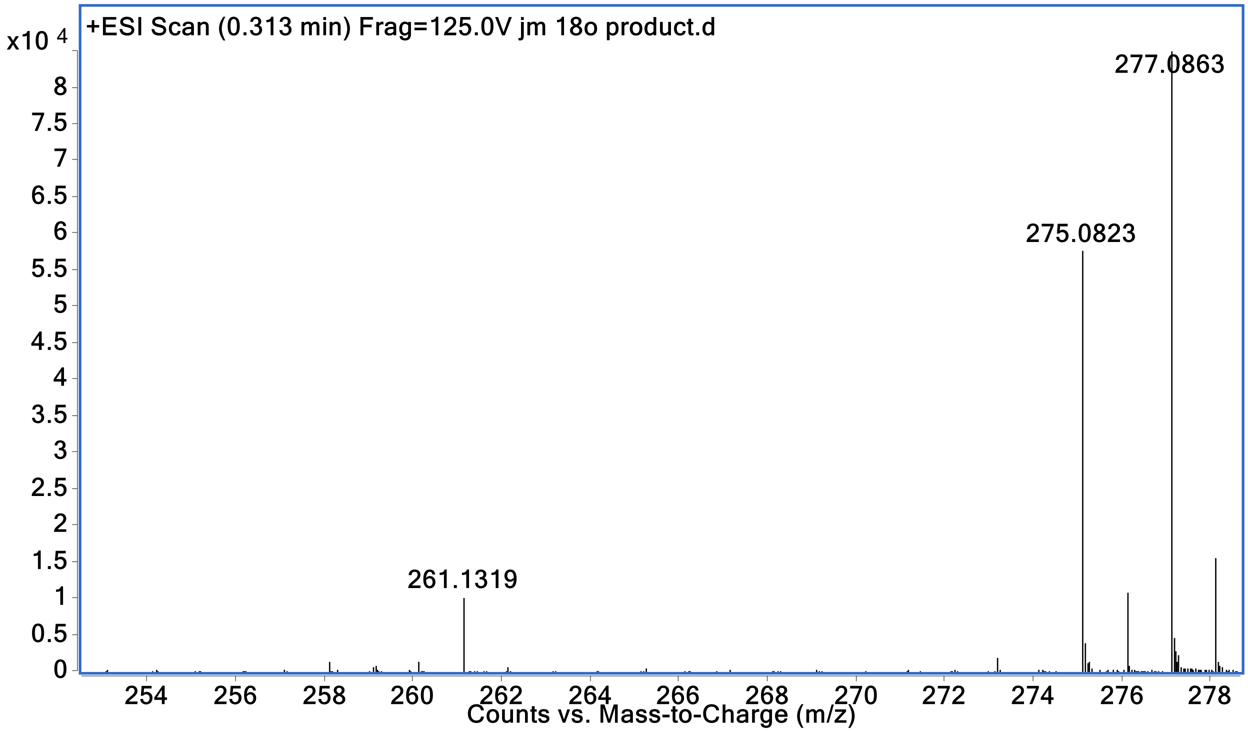

References

1. (a) Wu, X.; Zhao, Y.; Ge, H. *J. Am. Chem. Soc.* **2014**, *136*, 1789. (b) Wu, X.; Zhao, Y.; Zhang, G.; Ge, H. *Angew. Chem., Int. Ed.* **2014**, *53*, 3706. (c) Wu, X.; Zhao, Y.; Ge, H. *Chem. Eur. J.* **2014**, *20*, 9530.
2. (a) Nichino, M.; Hirano, K.; Satoh, T.; Miura, M. *Angew. Chem., Int. Ed.* **2013**, *52*, 4457. (b) Grigorjeva, L.; Daugulis, O. *Angew. Chem., Int. Ed.* **2014**, *53*, 10209. (c) Grigorjeva, L.; Daugulis, O. *Org. Lett.* **2014**, *16*, 4684.
3. Walvoord, R. R.; Berritt, S.; Kozlowski, M. C. *Org. Lett.* **2012**, *14*, 4086.
4. Wu, X.; Zhao, Y.; Ge, H. *J. Am. Chem. Soc.* **2015**, *137*, 4924.
5. Grigorjeva, L.; Daugulis, O. *Org. Lett.* **2014**, *16*, 4688.

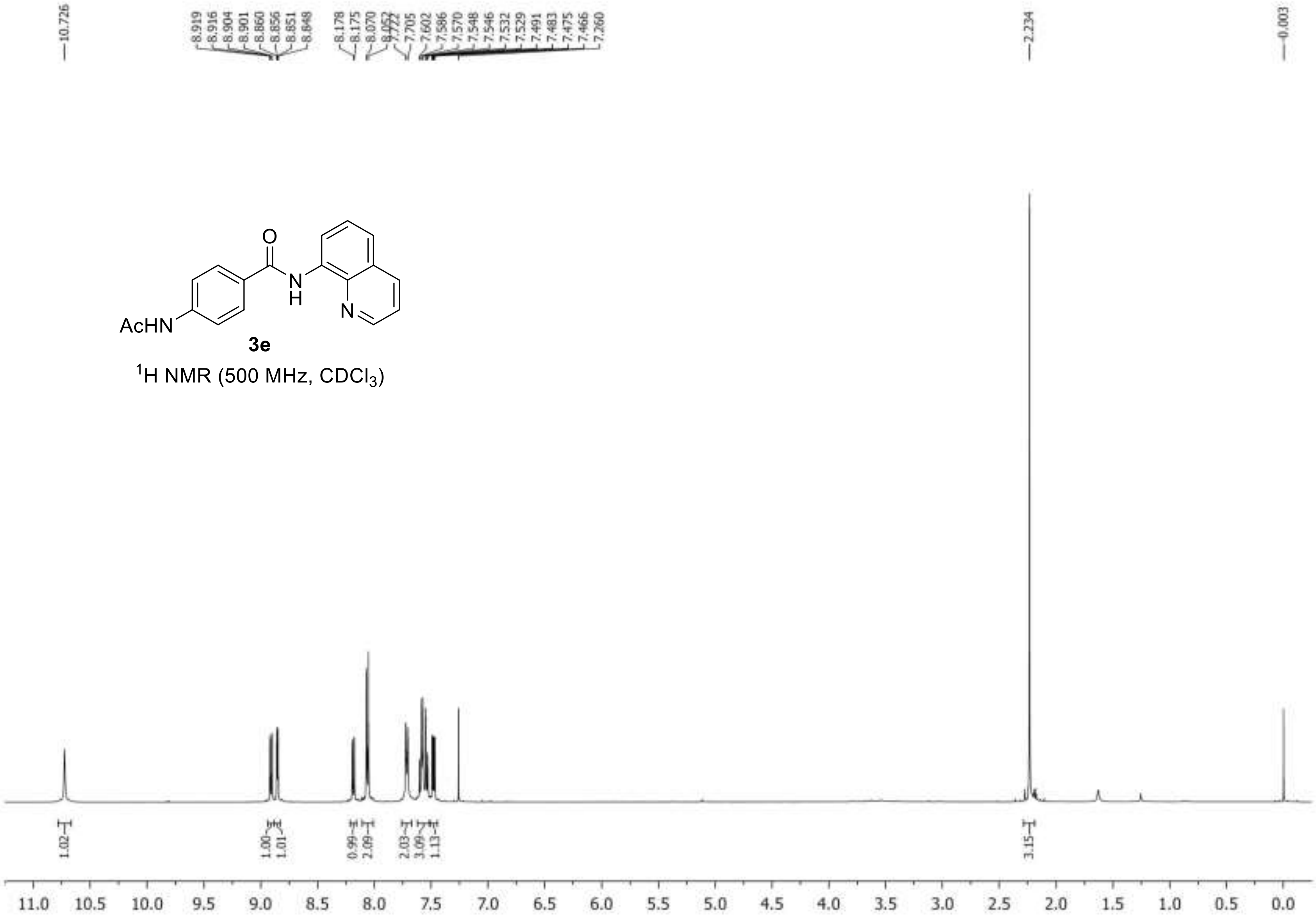

3e

^{1}H NMR (500 MHz, $CDCl_3$)

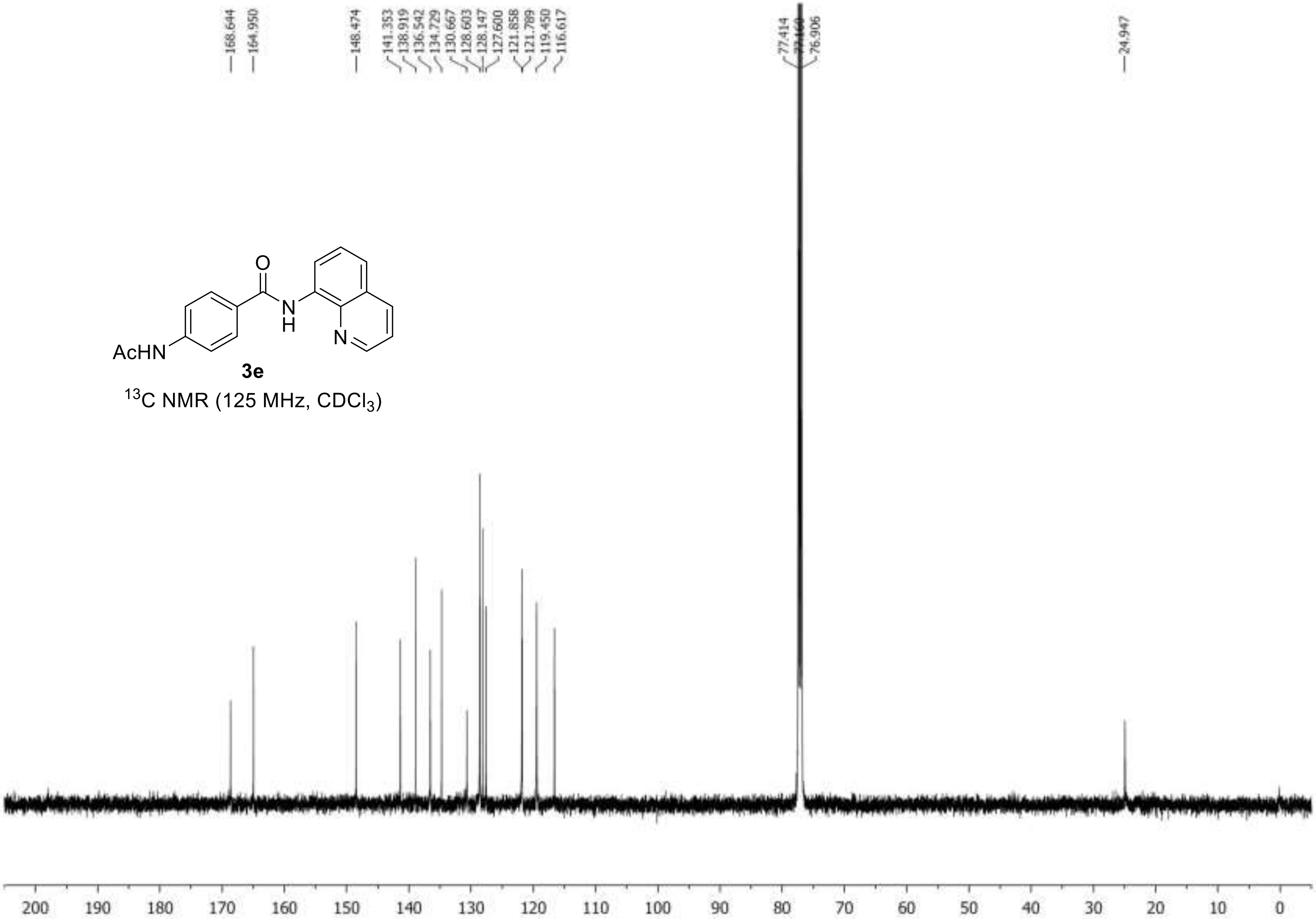

3e

^{13}C NMR (125 MHz, $CDCl_3$)

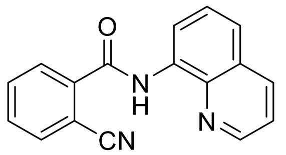

5

^1H NMR (500 MHz, $CDCl_3$)

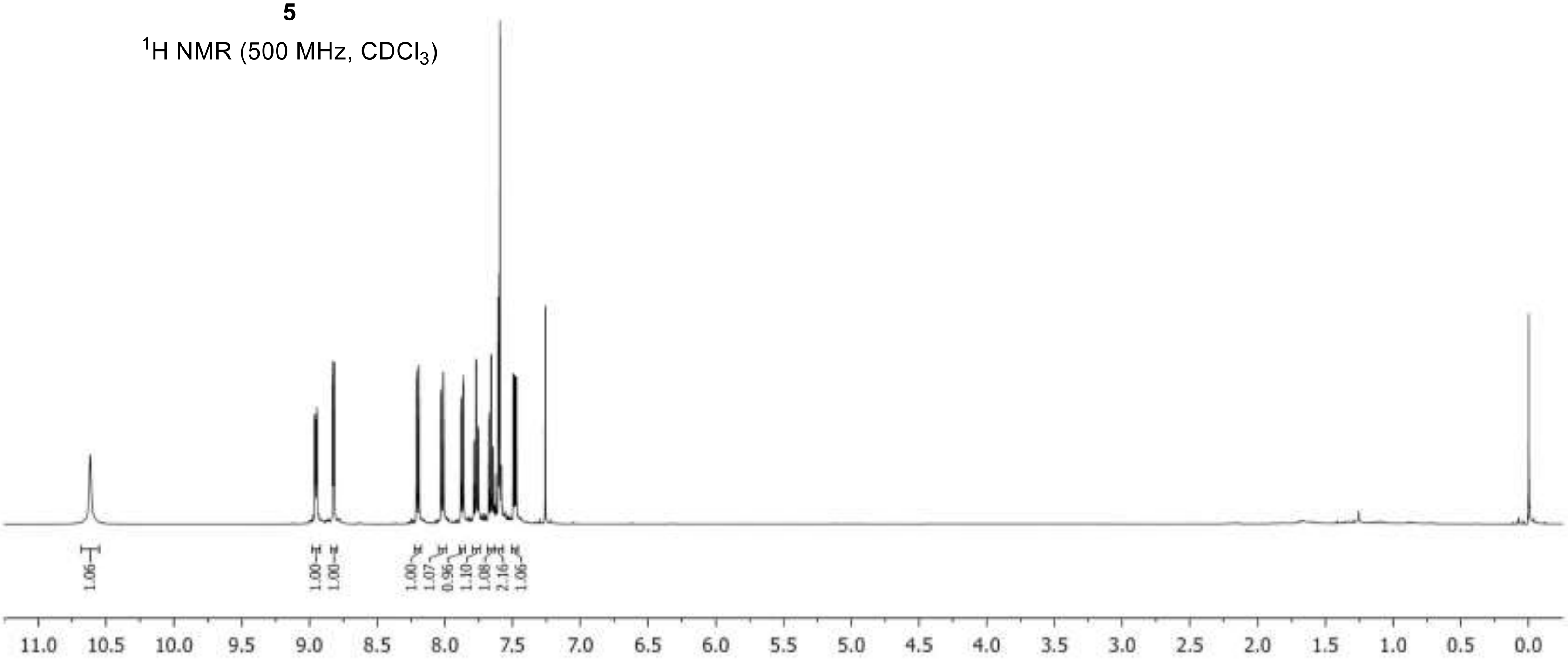

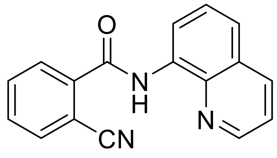

5

^{13}C NMR (125 MHz, $CDCl_3$)

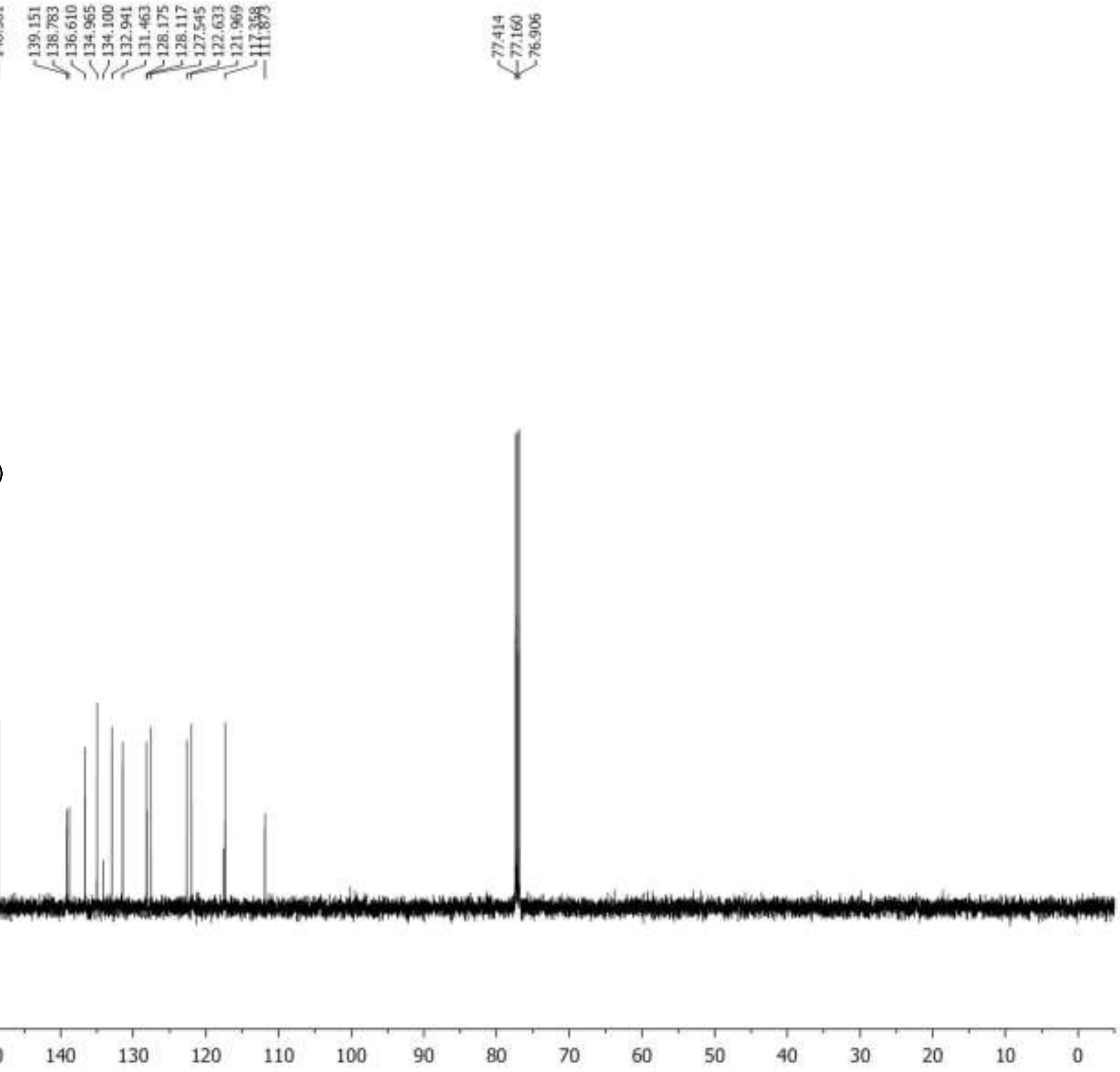

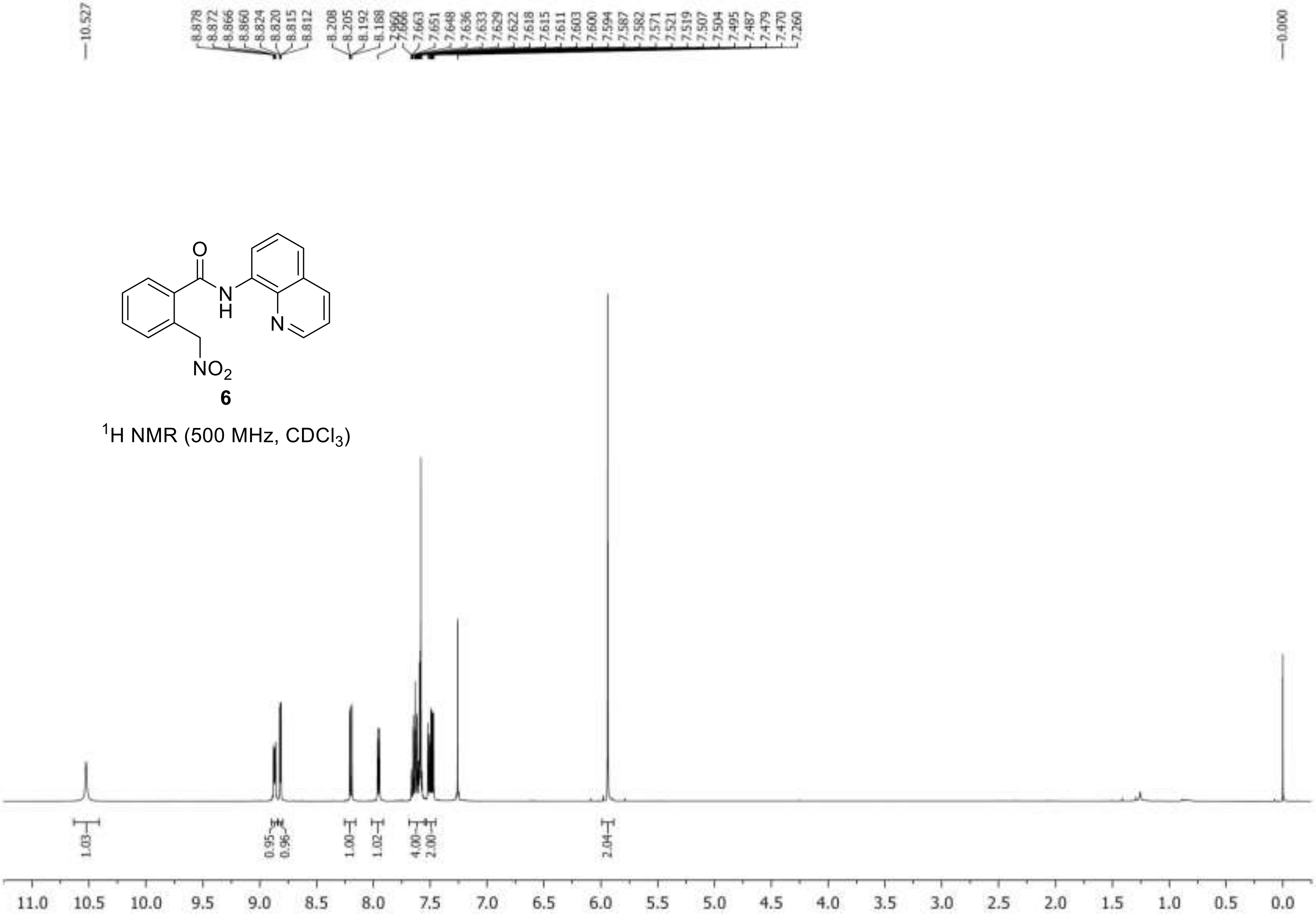

6

^{1}H NMR (500 MHz, $CDCl_3$)

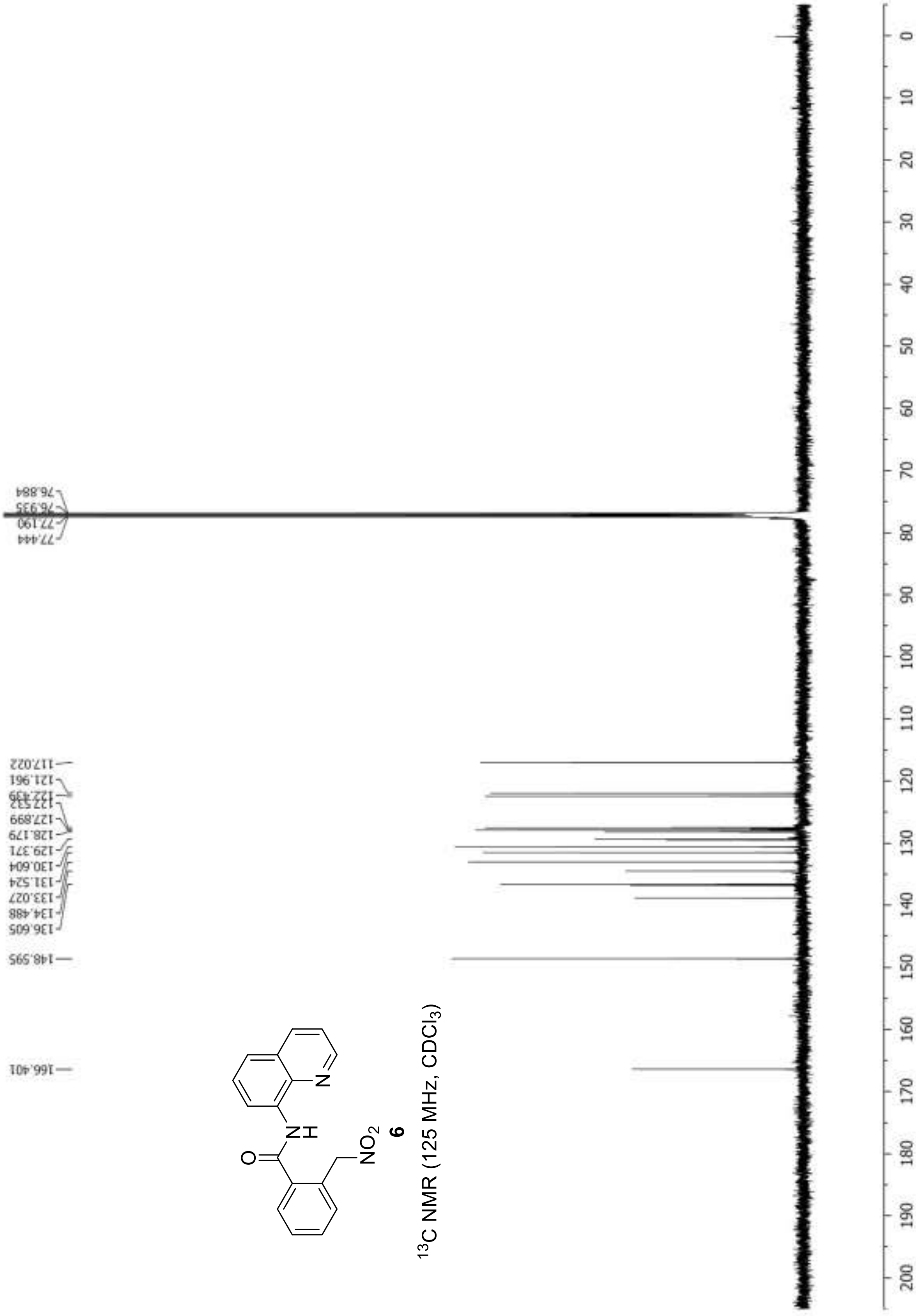

6

^{13}C NMR (125 MHz, $CDCl_3$)

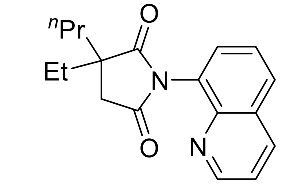

2a

^{1}H NMR (500 MHz, $CDCl_3$)

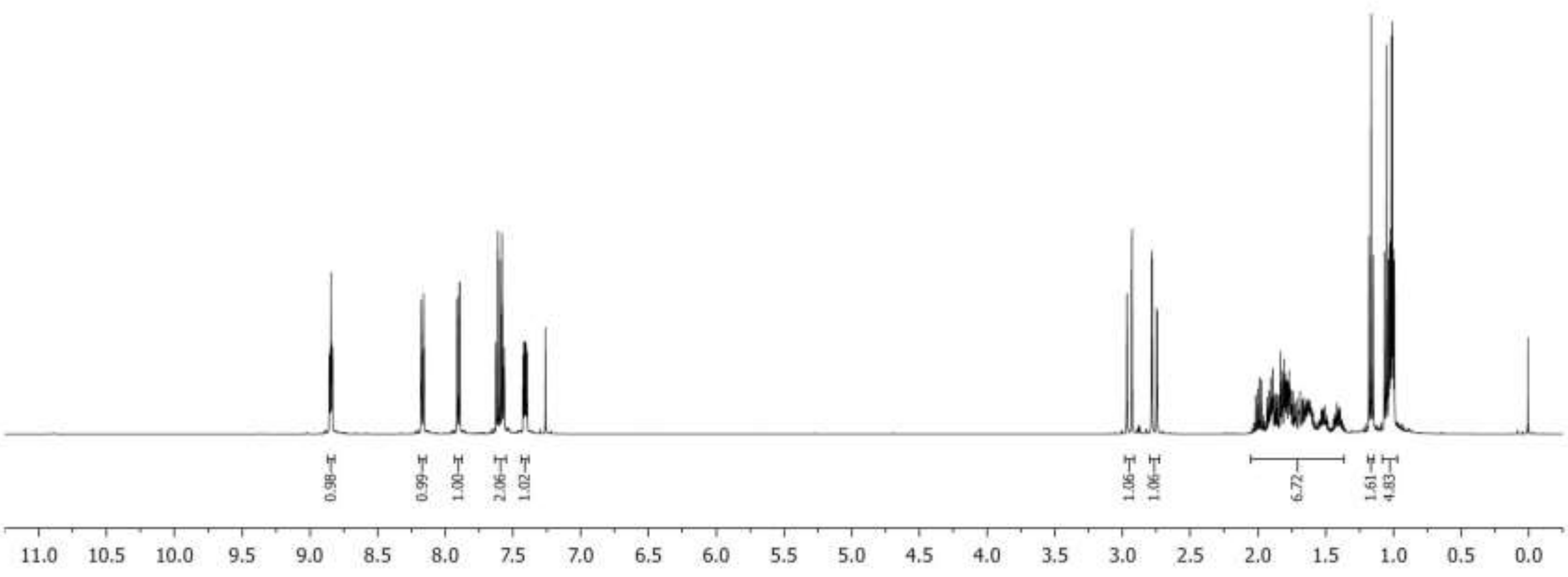

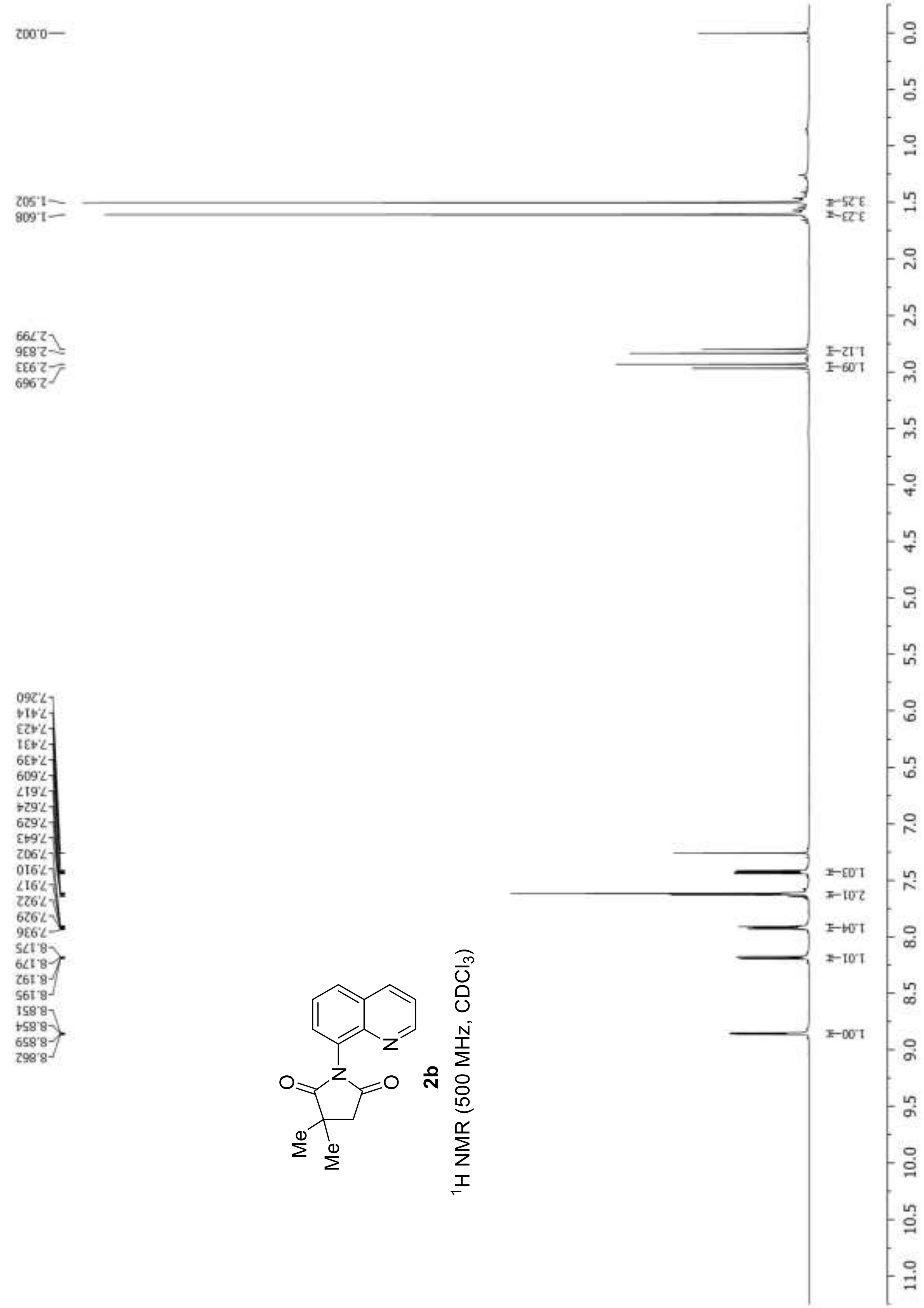

2b

^{1}H NMR (500 MHz, $CDCl_3$)

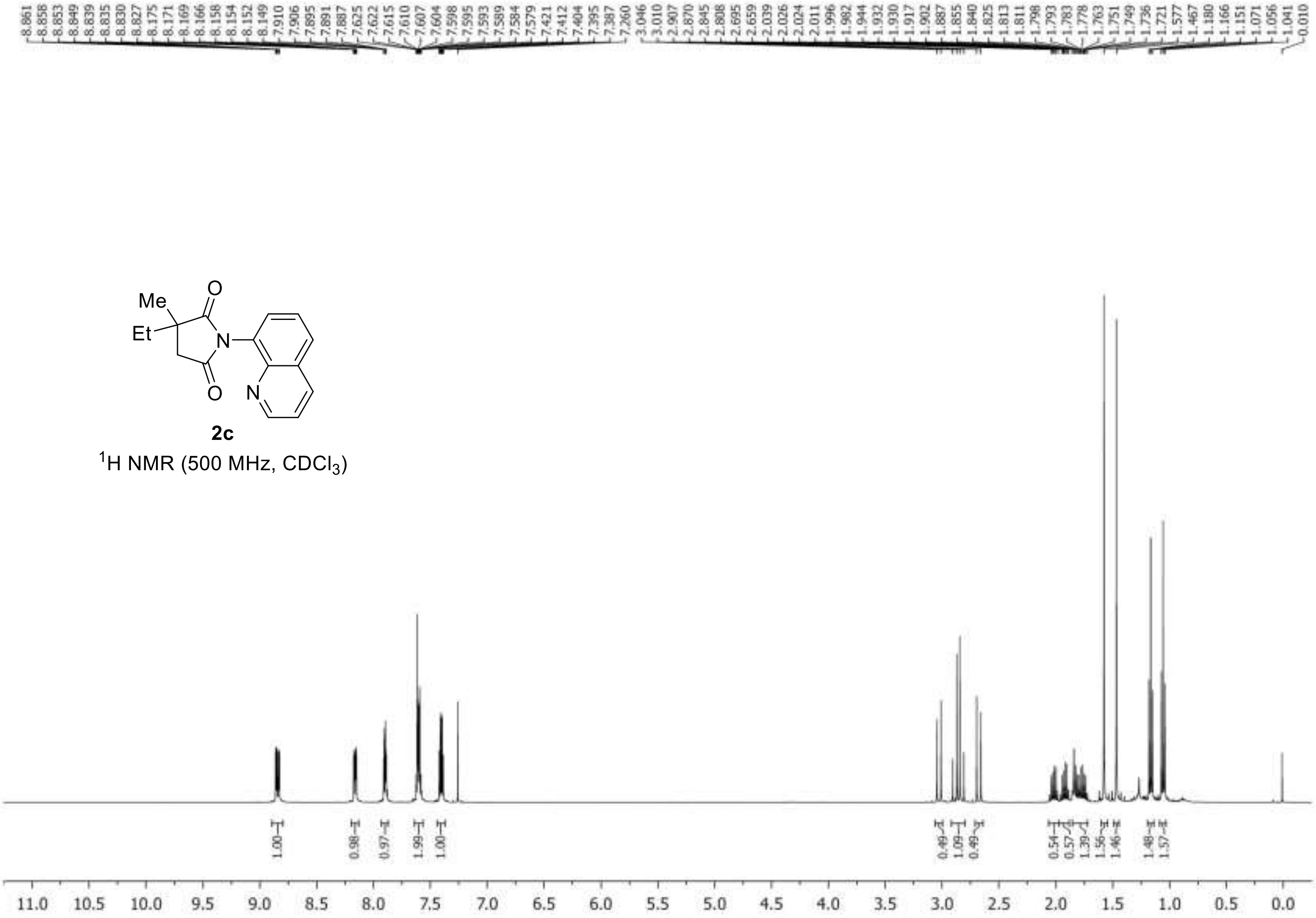

2c

^1H NMR (500 MHz, $CDCl_3$)

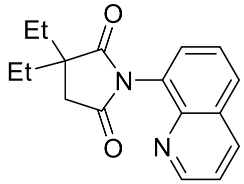

2d

^{1}H NMR (500 MHz, $CDCl_3$)

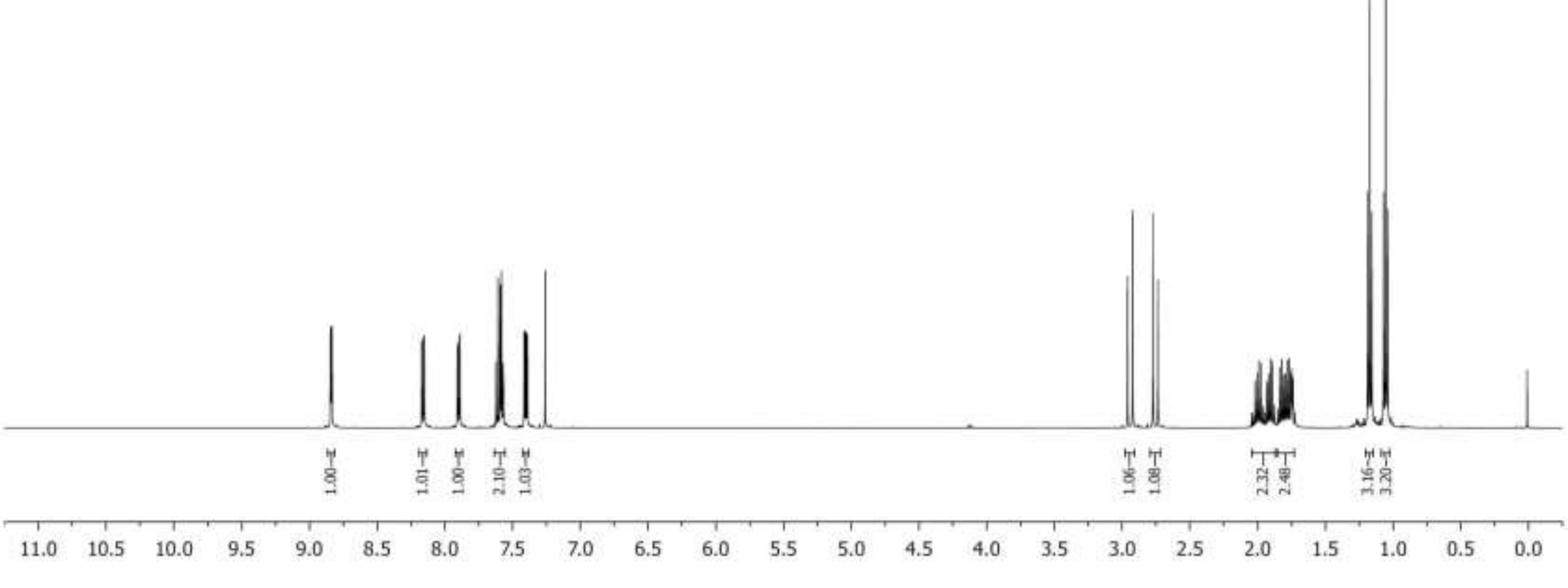

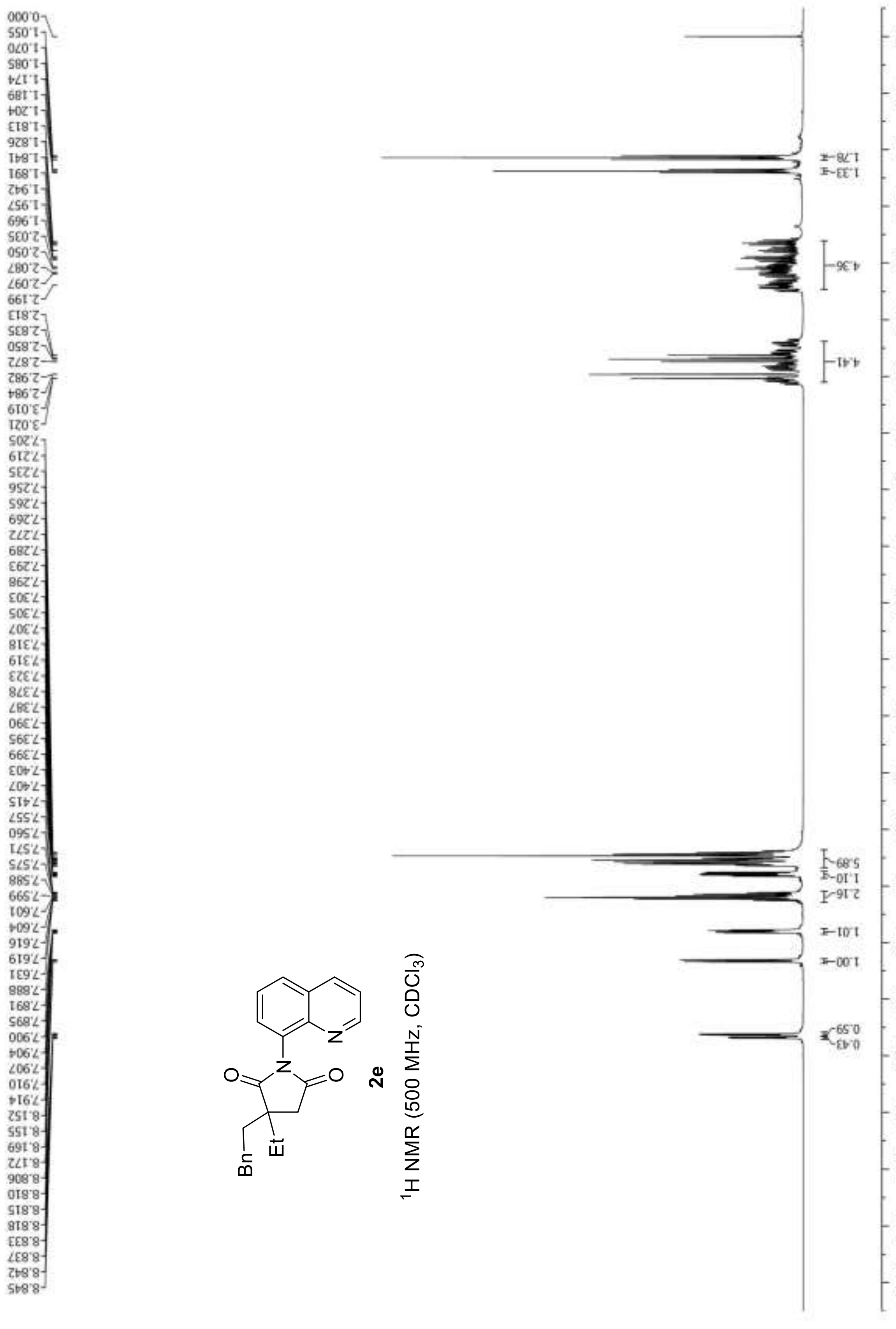

2e

^{1}H NMR (500 MHz, $CDCl_3$)

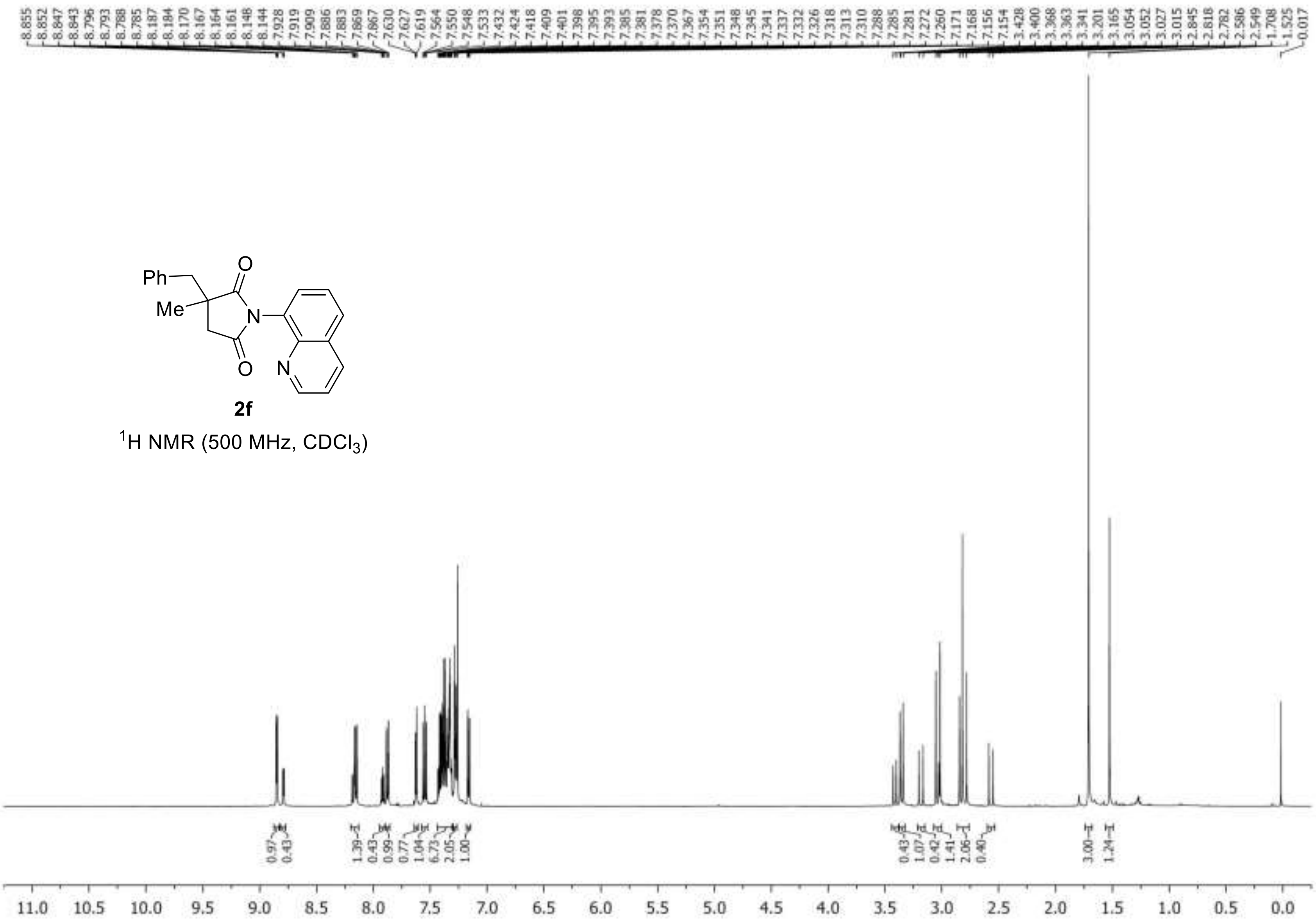

2f

^{1}H NMR (500 MHz, $CDCl_3$)

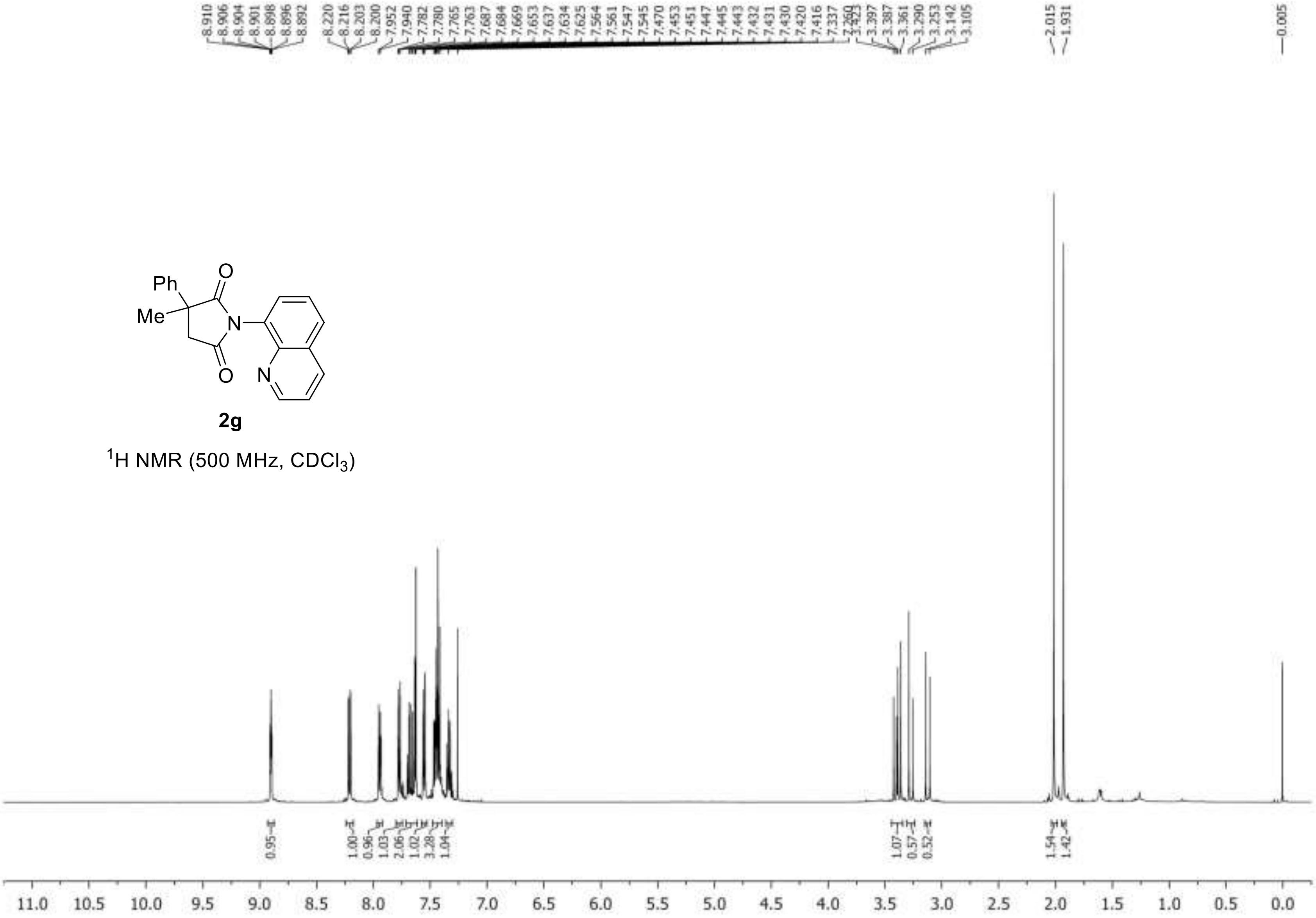

2g

^{1}H NMR (500 MHz, $CDCl_3$)

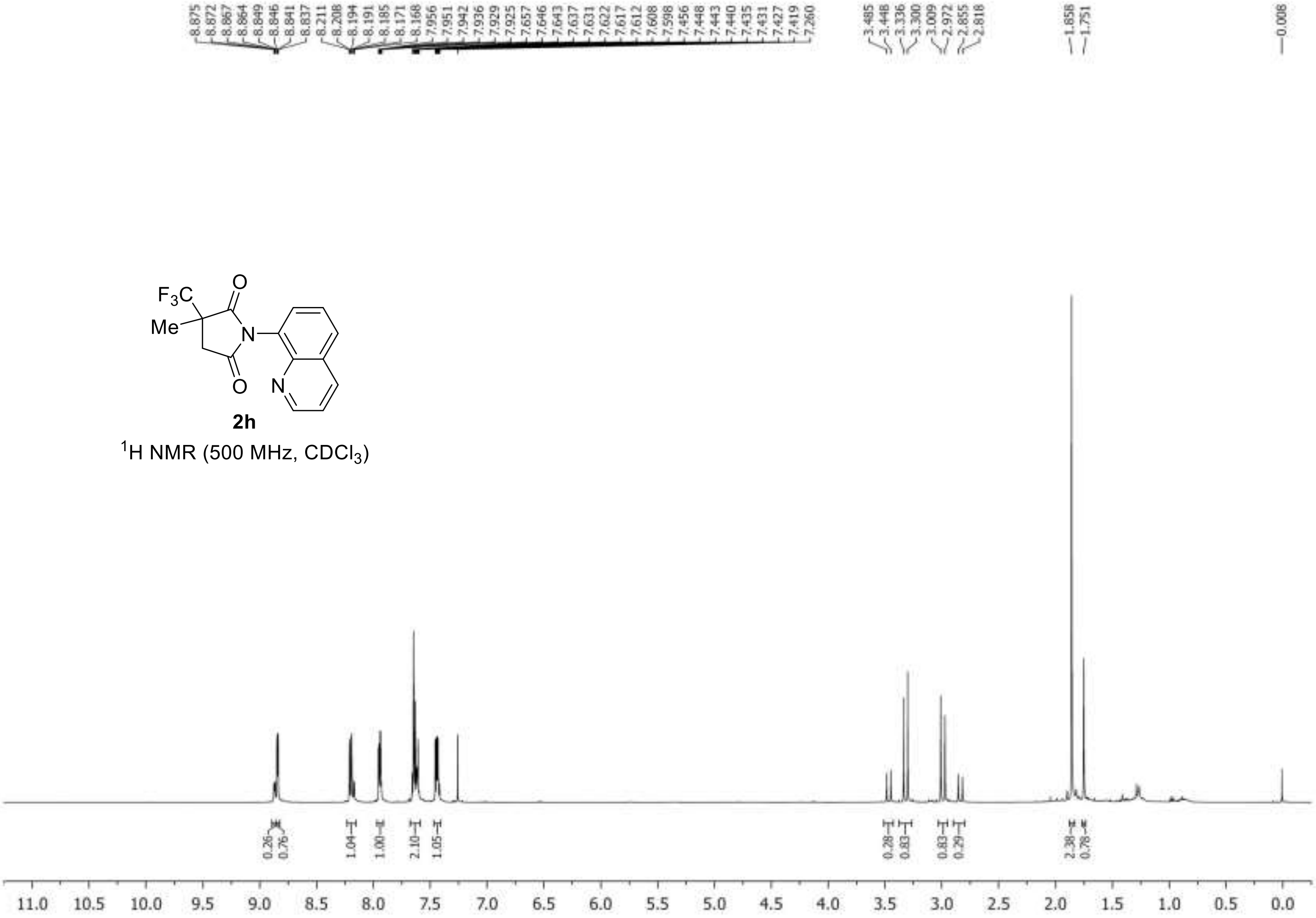

2h

^{1}H NMR (500 MHz, $CDCl_3$)

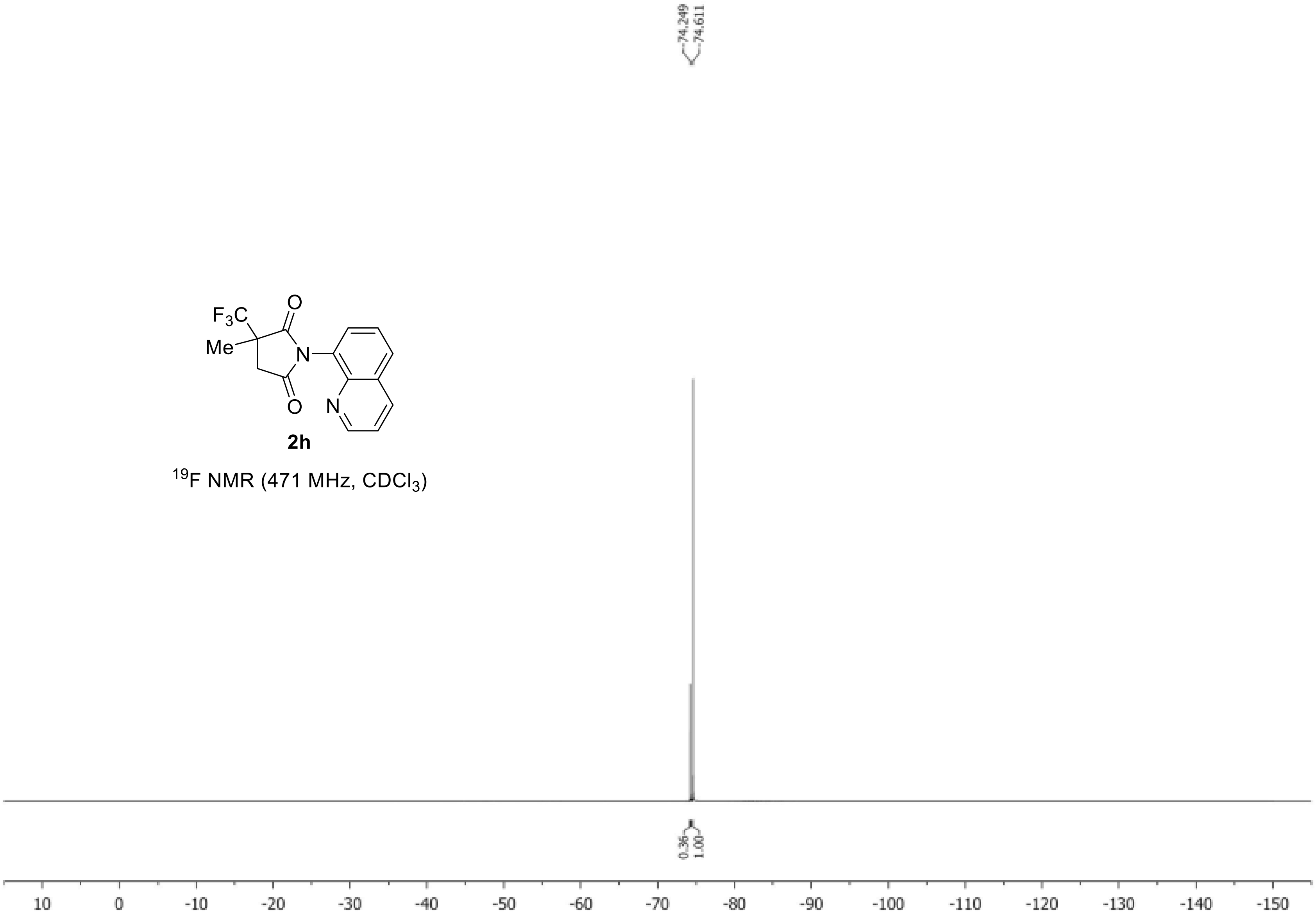

2h

^{19}F NMR (471 MHz, $CDCl_3$)

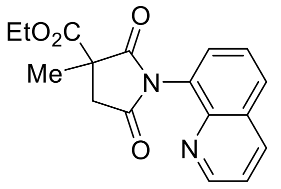

2i

^{1}H NMR (500 MHz, $CDCl_3$)

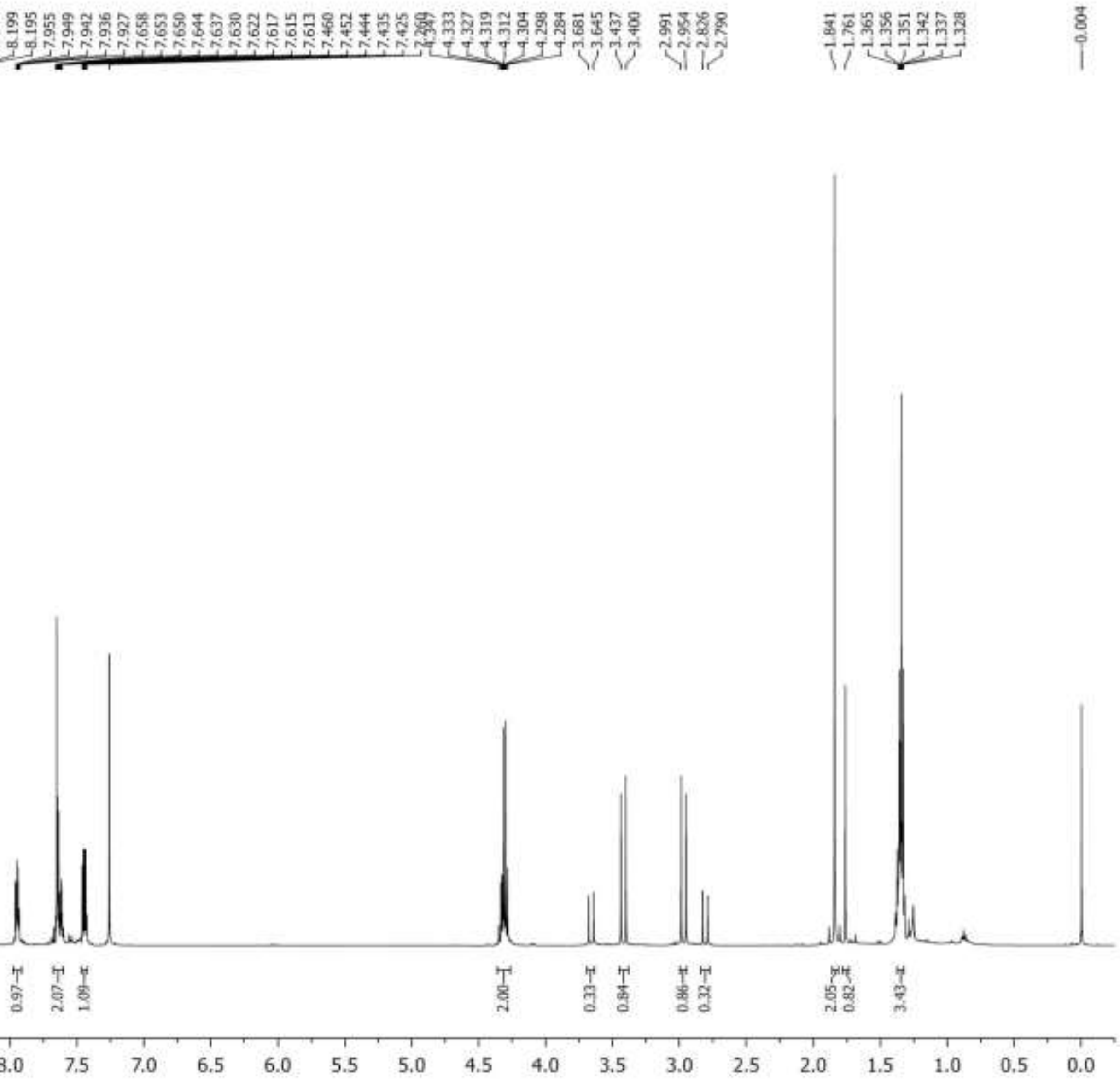

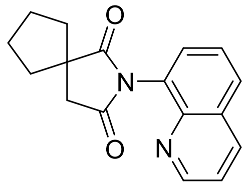

2j

^{1}H NMR (500 MHz, $CDCl_3$)

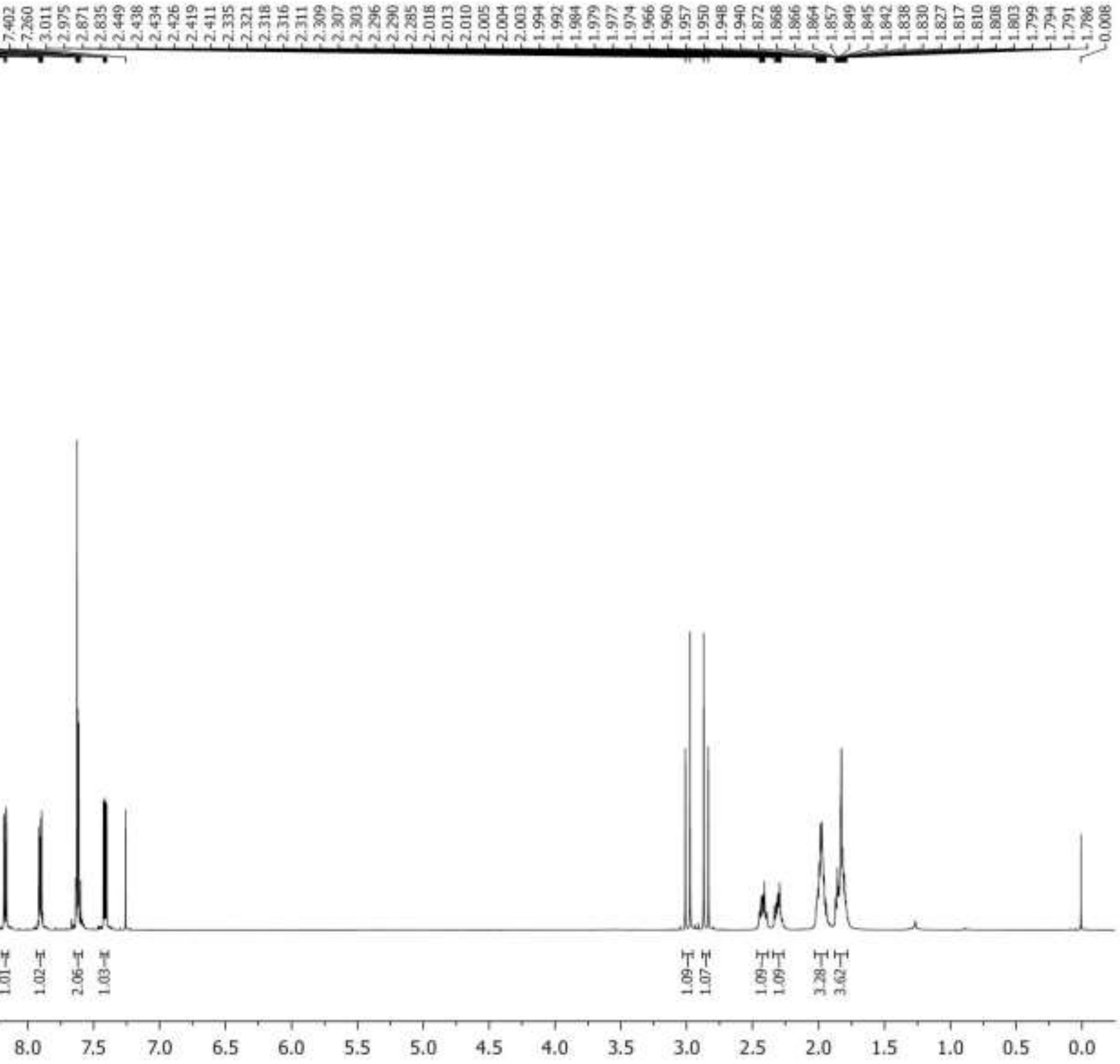

2k

^{1}H NMR (500 MHz, $CDCl_3$)

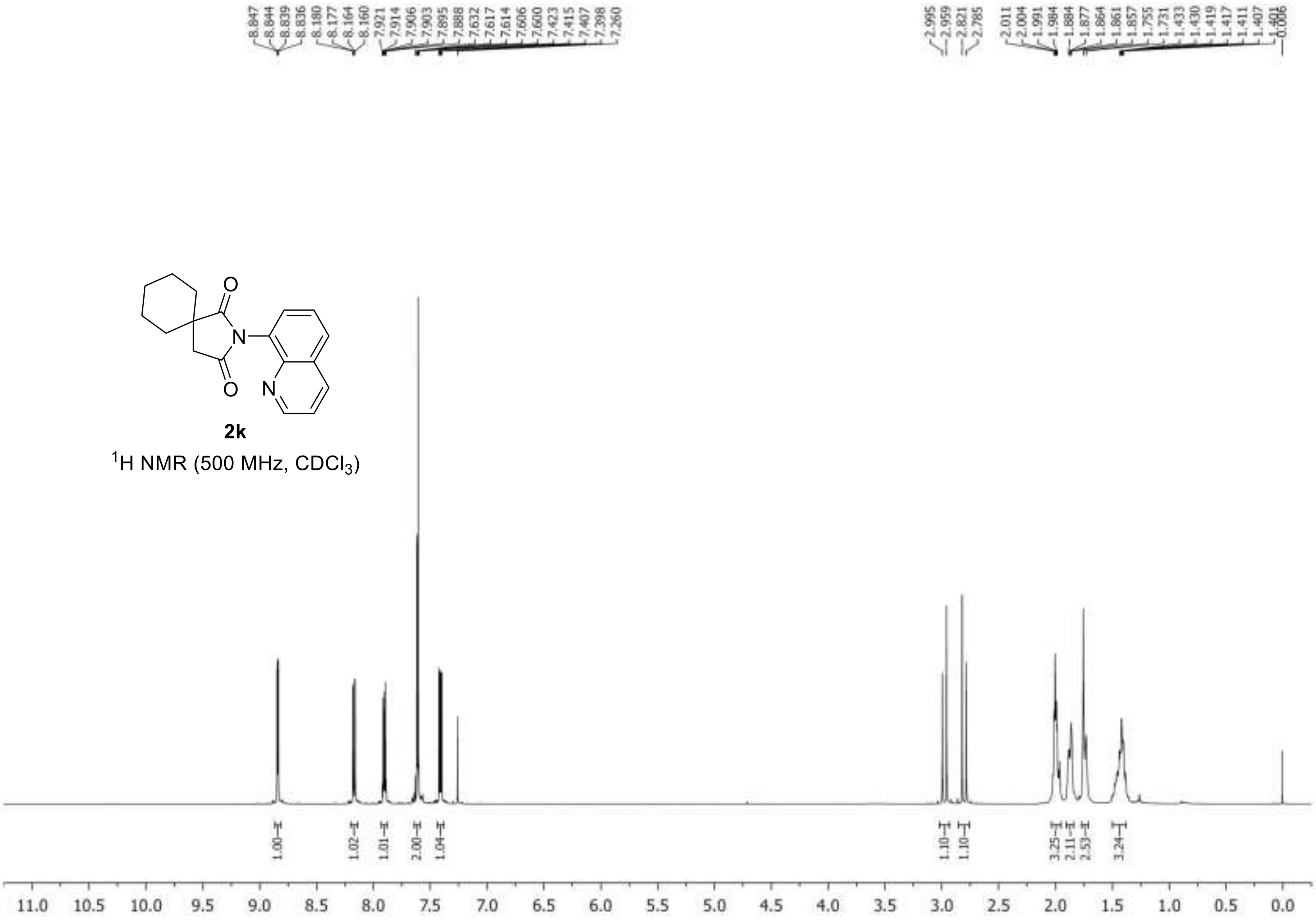

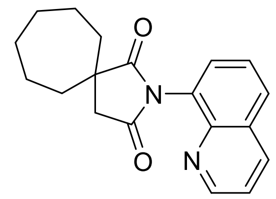

2l

^{1}H NMR (500 MHz, $CDCl_3$)

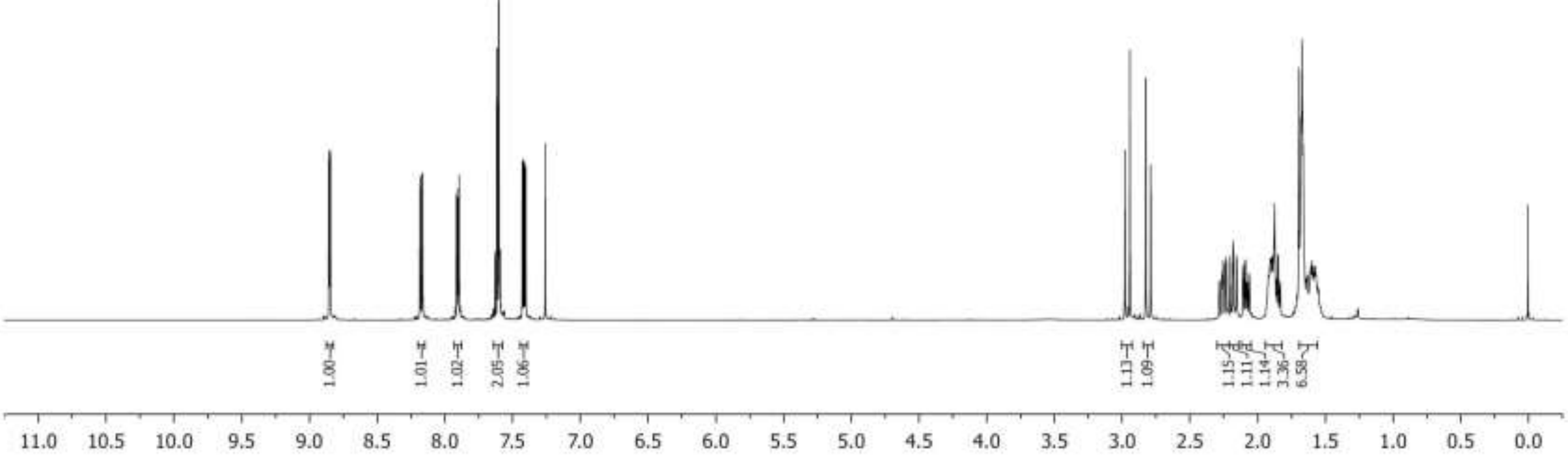

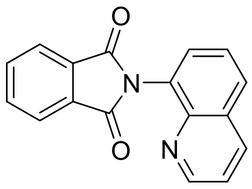

4a

^{1}H NMR (500 MHz, $CDCl_3$)

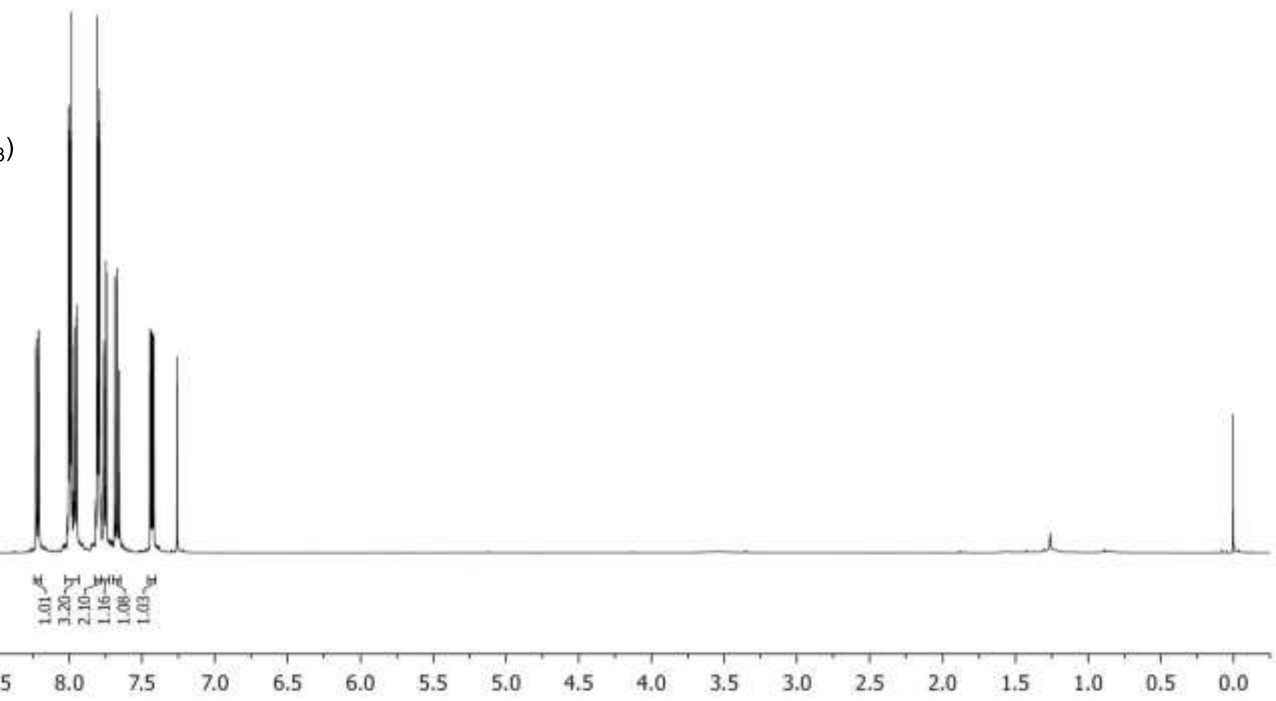

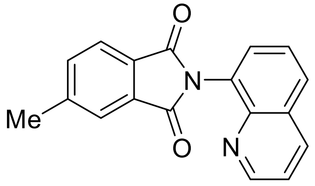

4b

^{1}H NMR (500 MHz, $CDCl_3$)

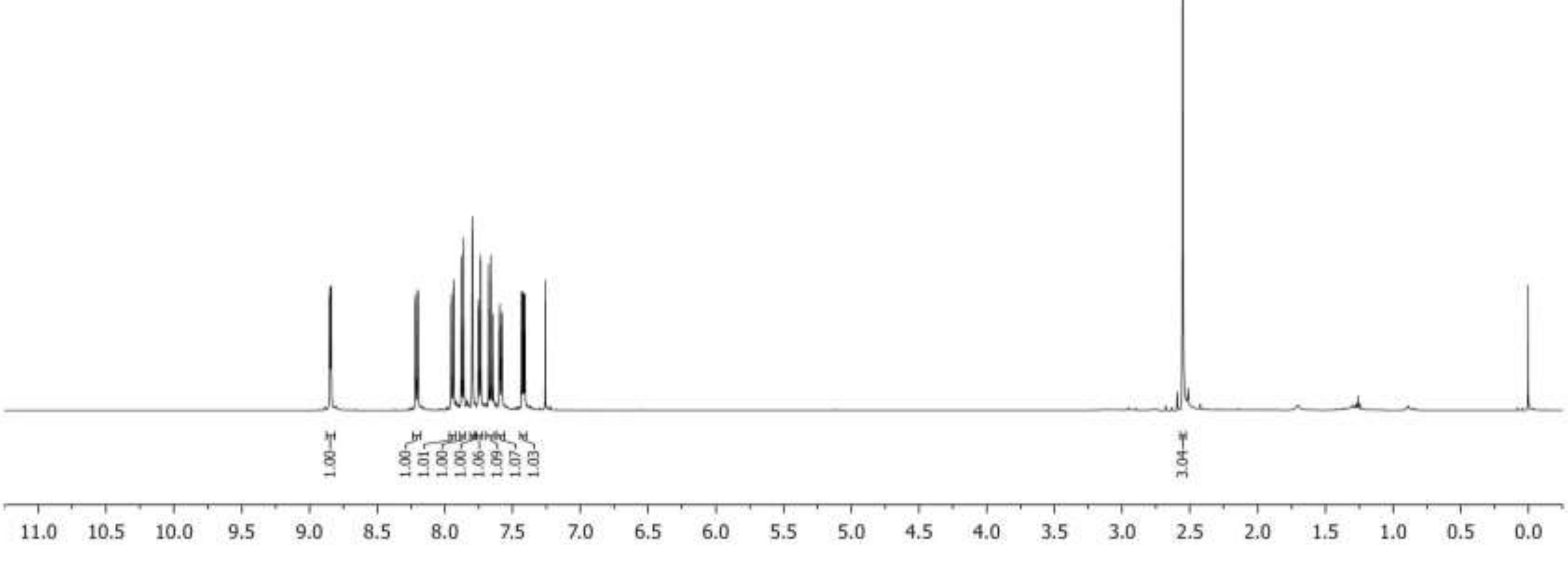

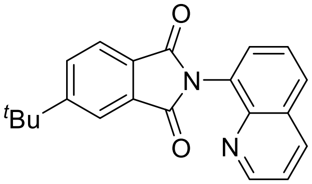

4c

^{1}H NMR (500 MHz, $CDCl_3$)

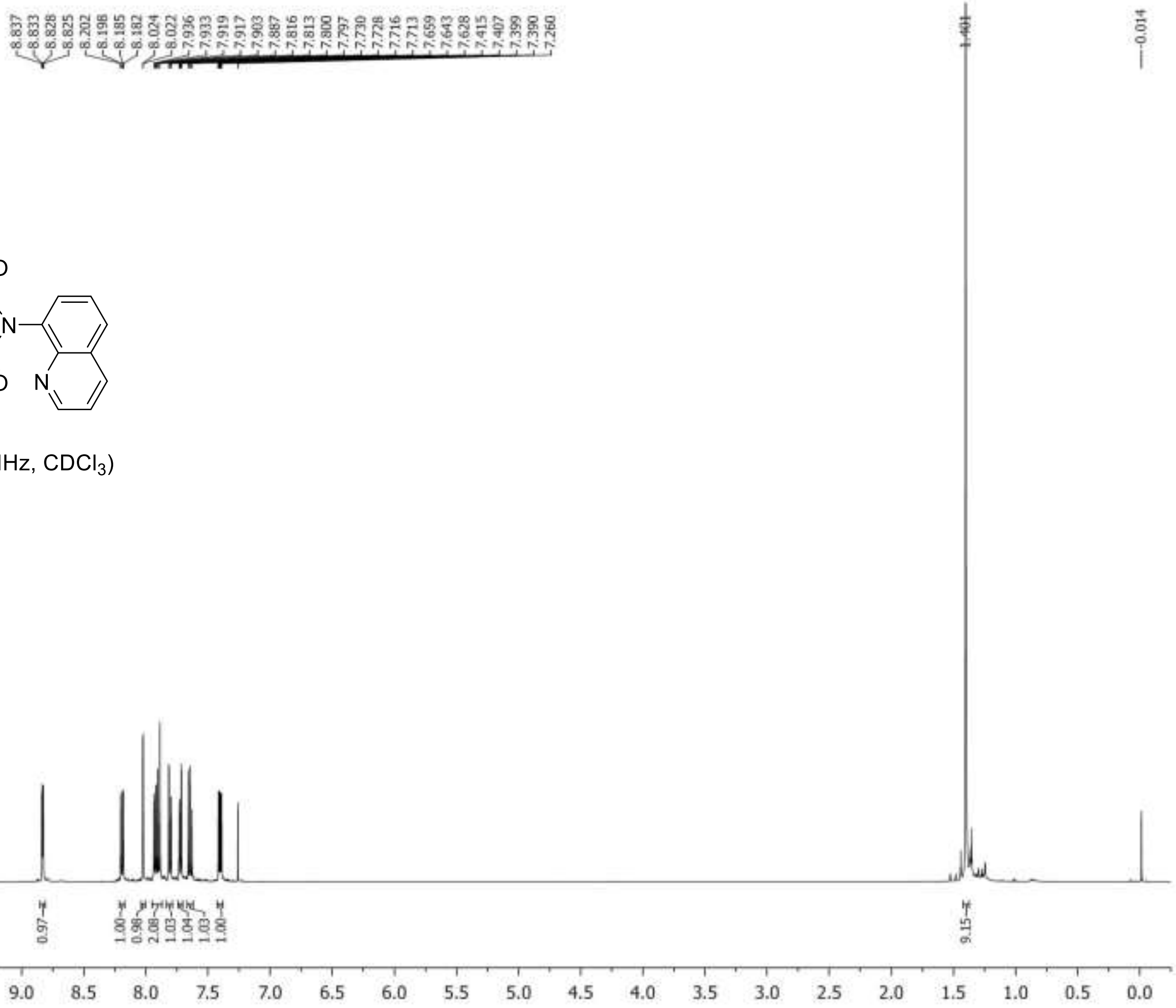

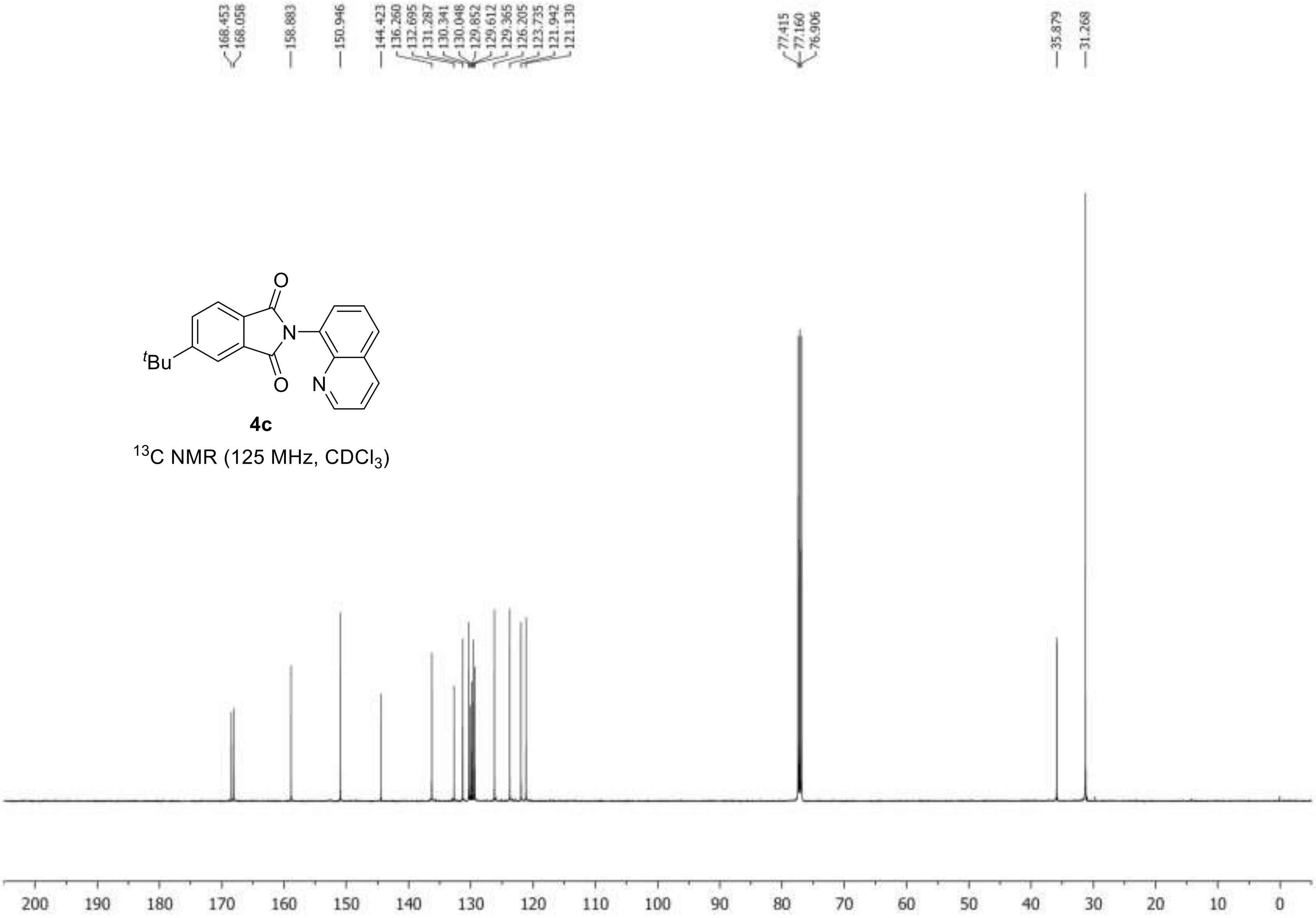

4c

^{13}C NMR (125 MHz, $CDCl_3$)

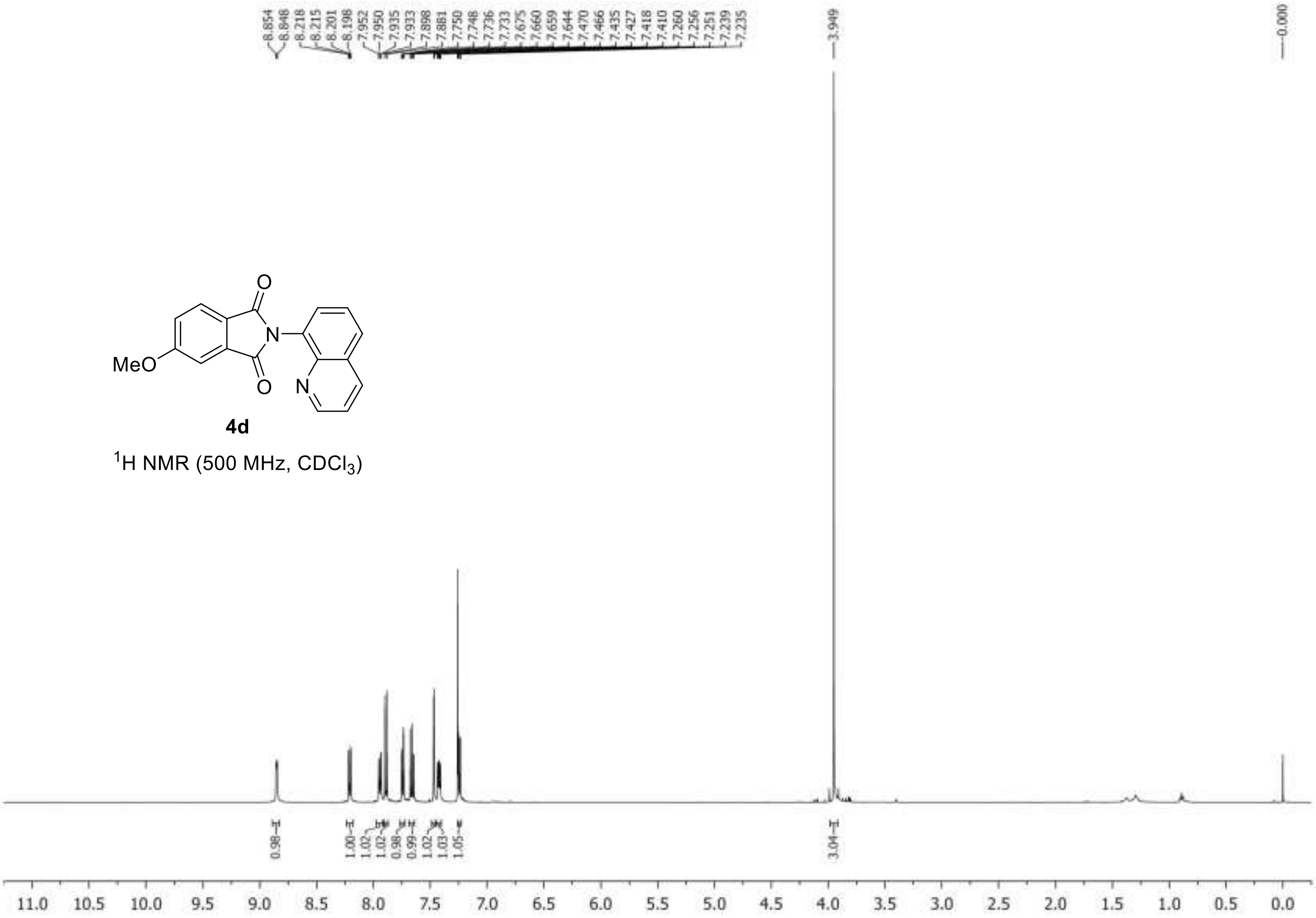

4d

^{1}H NMR (500 MHz, $CDCl_3$)

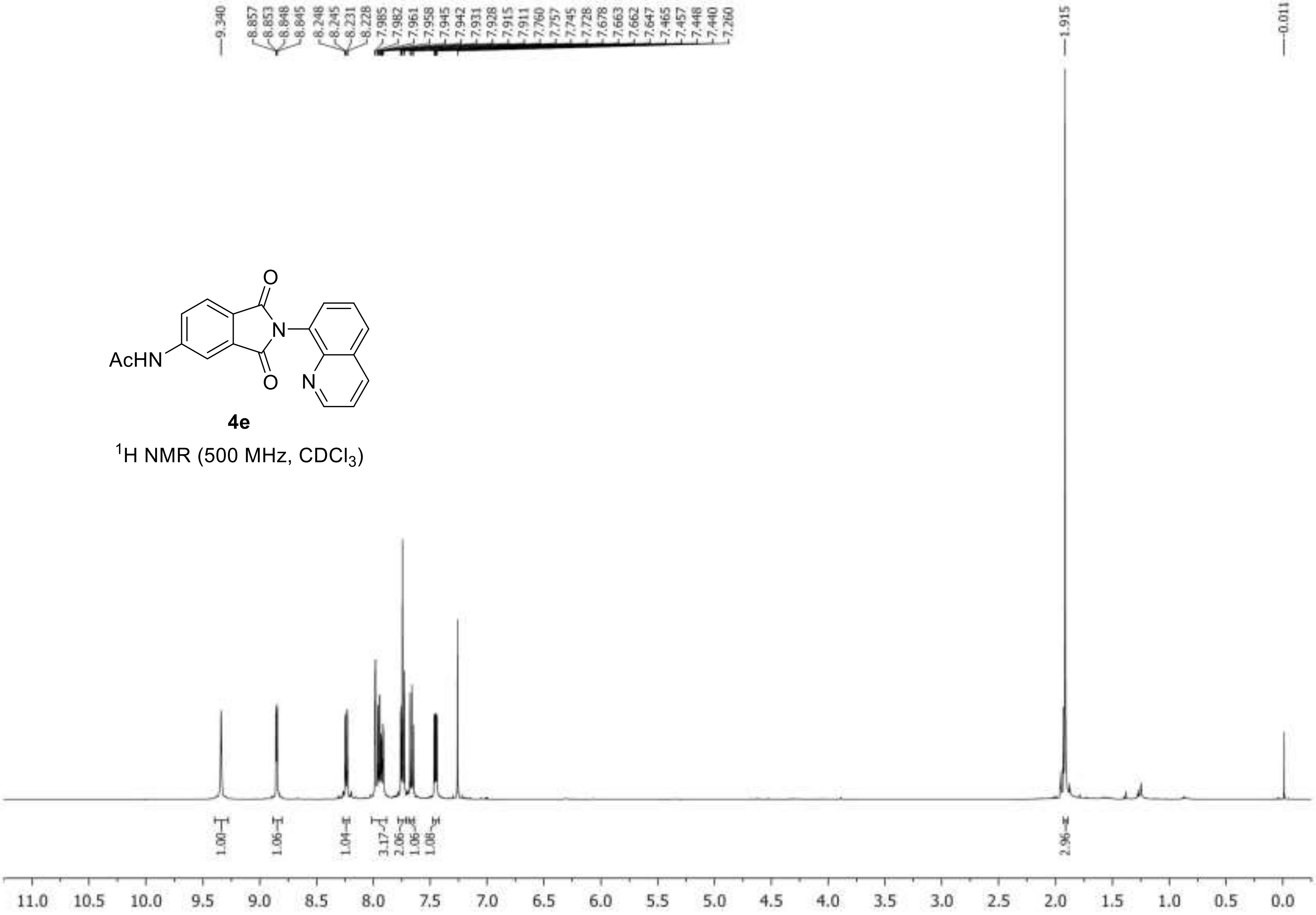

O
N
AcHN
O
N
4e
1H NMR (500 MHz, CDCl3)
9.340
8.857
8.853
8.848
8.845
8.248
8.245
8.231
8.228
7.985
7.982
7.961
7.958
7.945
7.942
7.931
7.928
7.915
7.911
7.760
7.757
7.745
7.728
7.678
7.663
7.662
7.647
7.465
7.457
7.448
7.440
7.260
1.915
-0.011
1.00
1.06
1.04
3.17
2.06
1.06
1.08
2.96
11.0 10.5 10.0 9.5 9.0 8.5 8.0 7.5 7.0 6.5 6.0 5.5 5.0 4.5 4.0 3.5 3.0 2.5 2.0 1.5 1.0 0.5 0.0

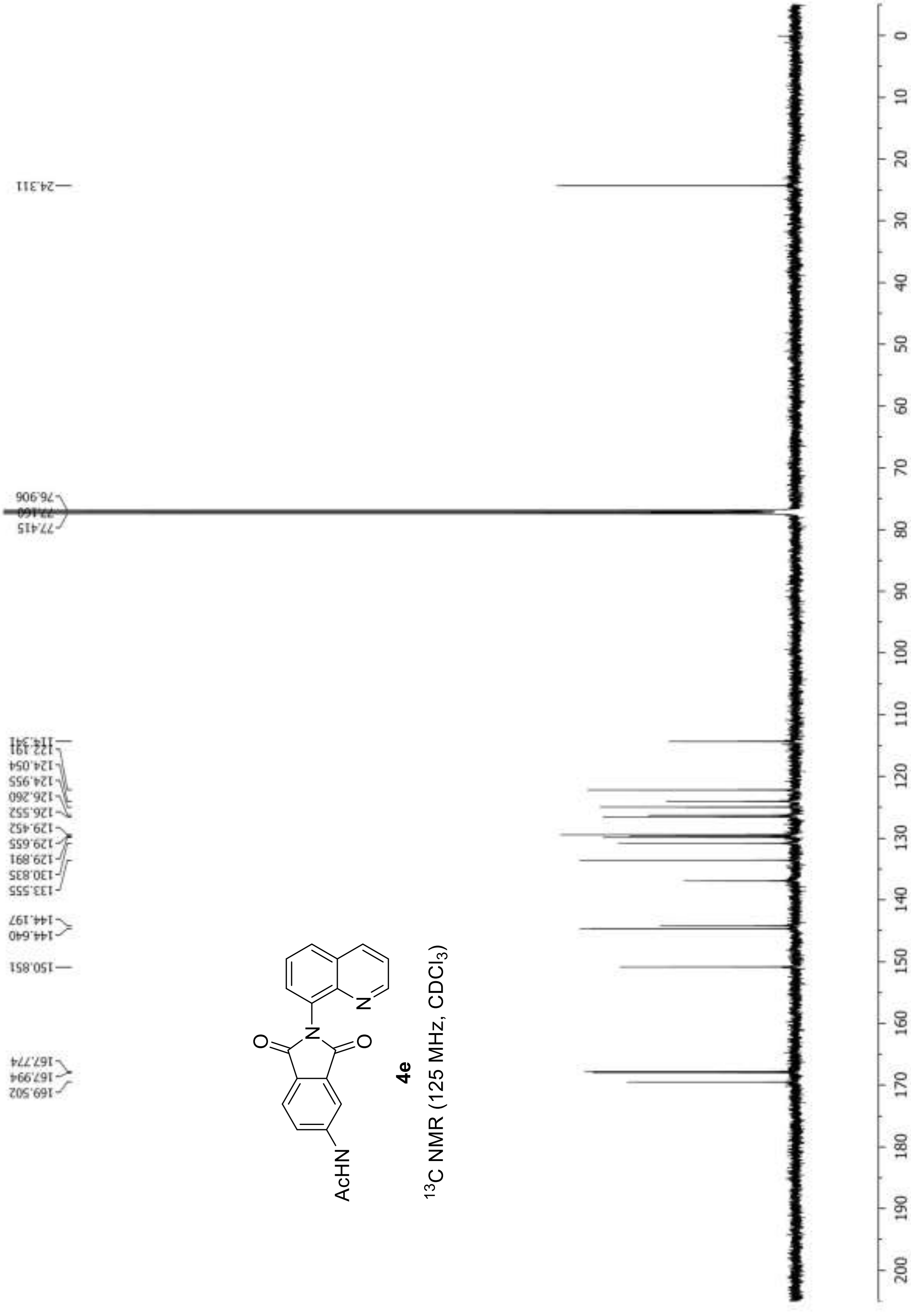

4e

^{13}C NMR (125 MHz, $CDCl_3$)

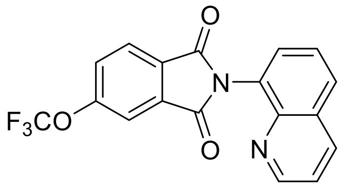

4f

^{1}H NMR (500 MHz, $CDCl_3$)

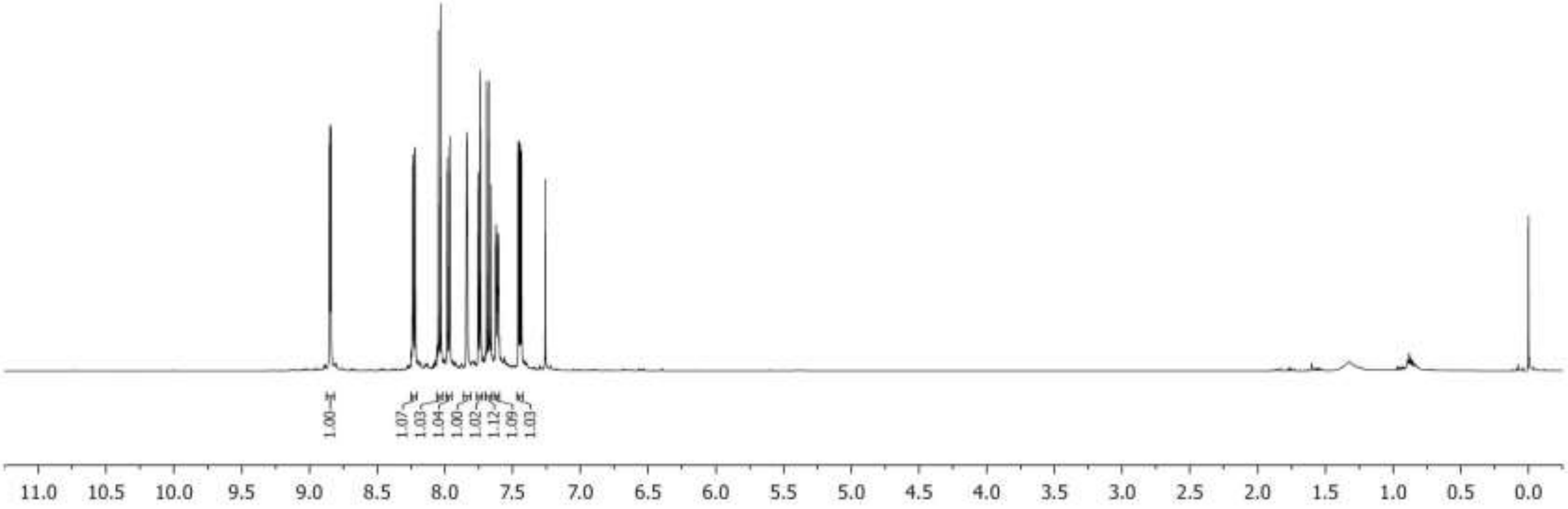

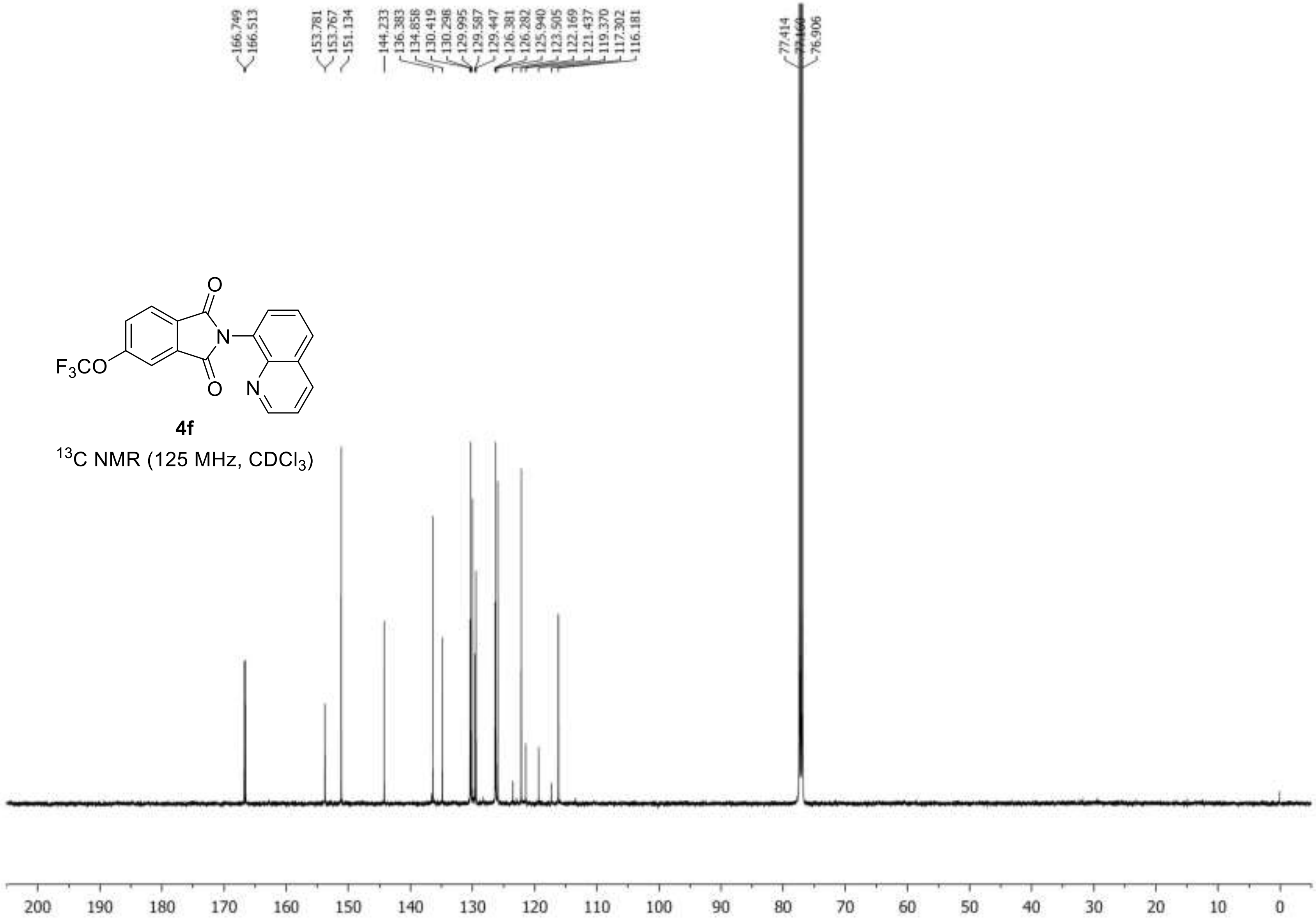

4f

^{13}C NMR (125 MHz, $CDCl_3$)

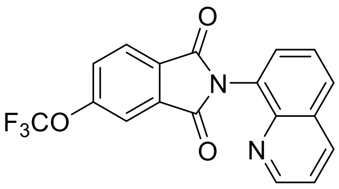

4f

^{19}F NMR (471 MHz, $CDCl_3$)

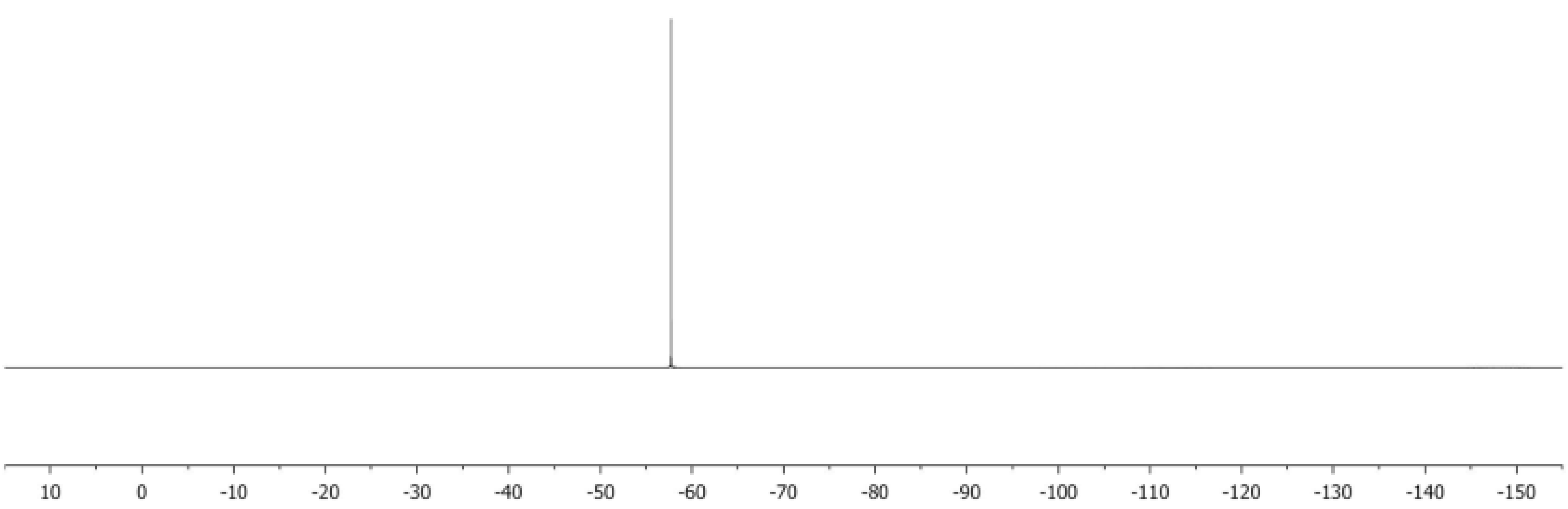

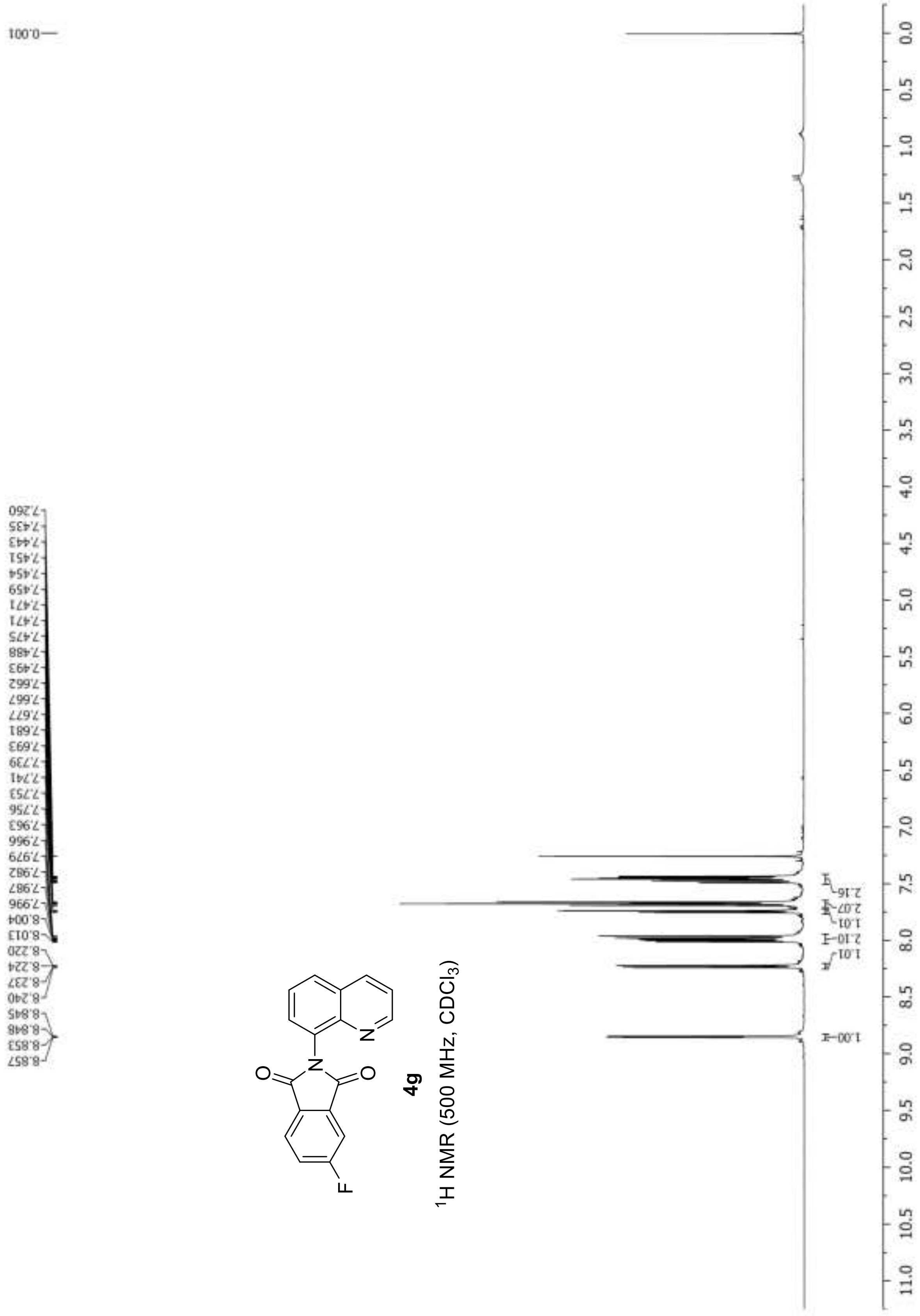

4g

^{1}H NMR (500 MHz, $CDCl_3$)

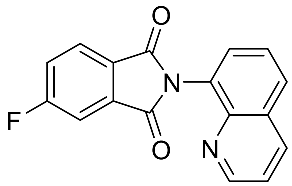

4g

^{19}F NMR (471 MHz, $CDCl_3$)

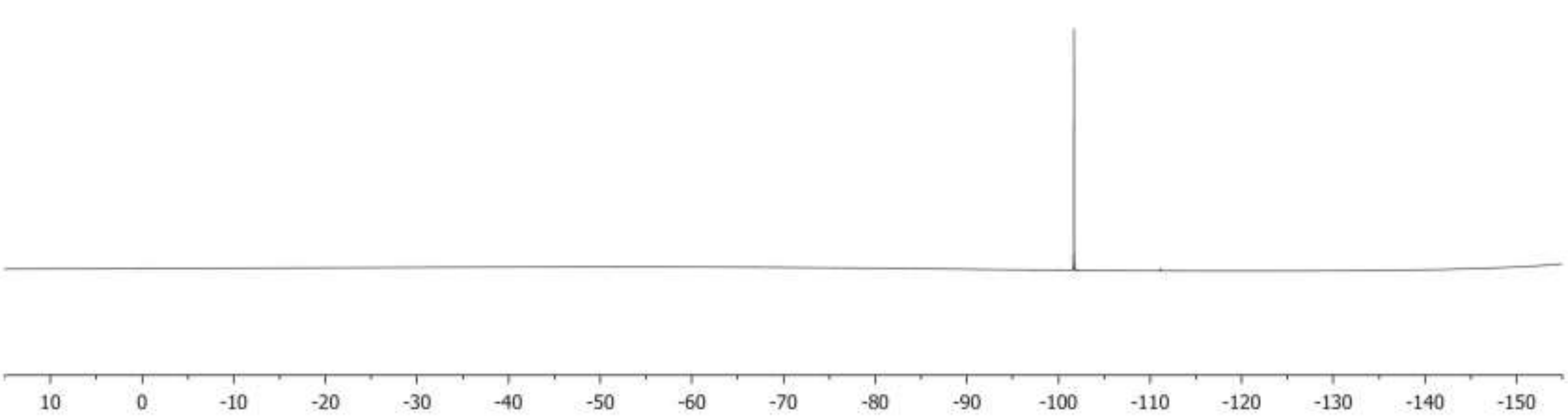

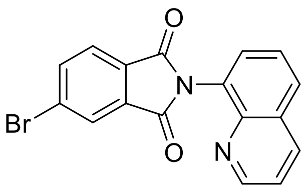

4h

^{1}H NMR (500 MHz, $CDCl_3$)

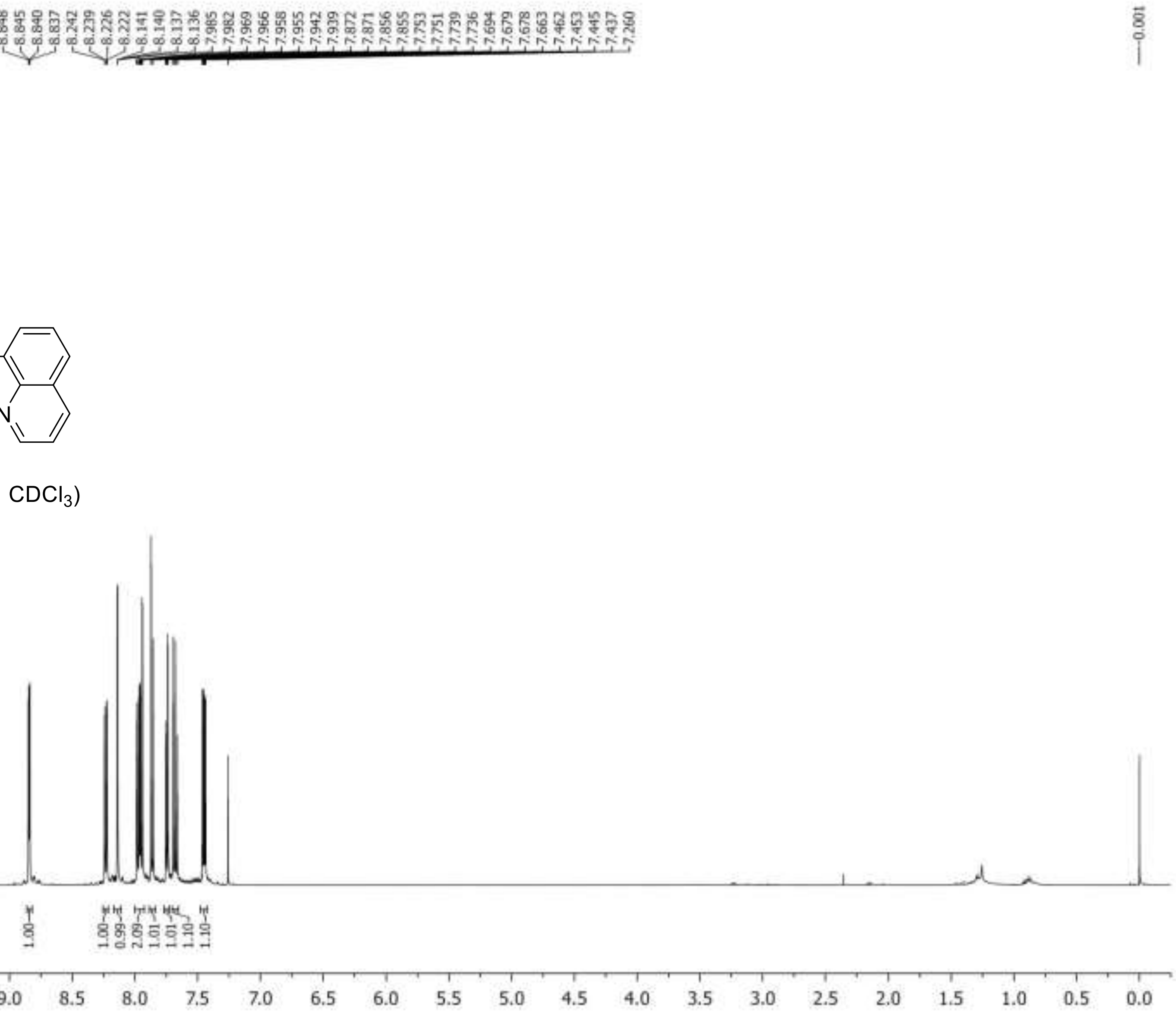

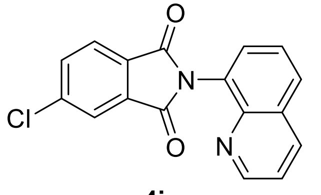

4i

^{1}H NMR (500 MHz, $CDCl_3$)

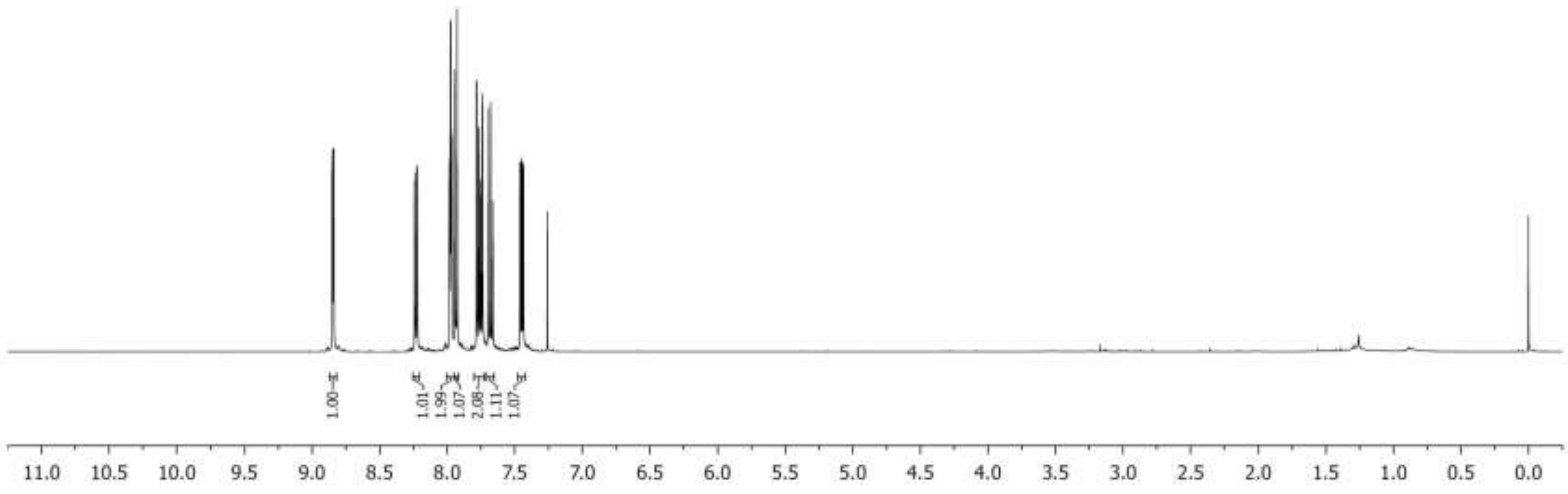

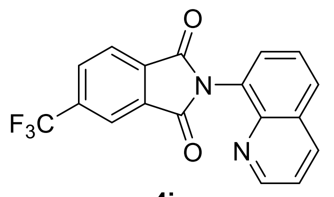

4j

^1H NMR (500 MHz, $CDCl_3$)

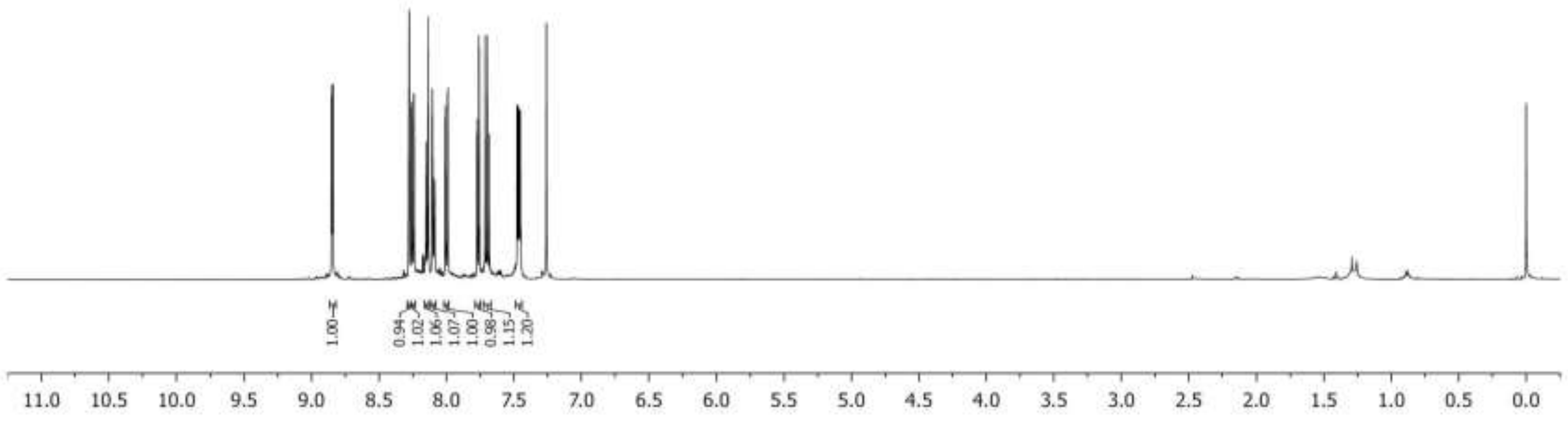

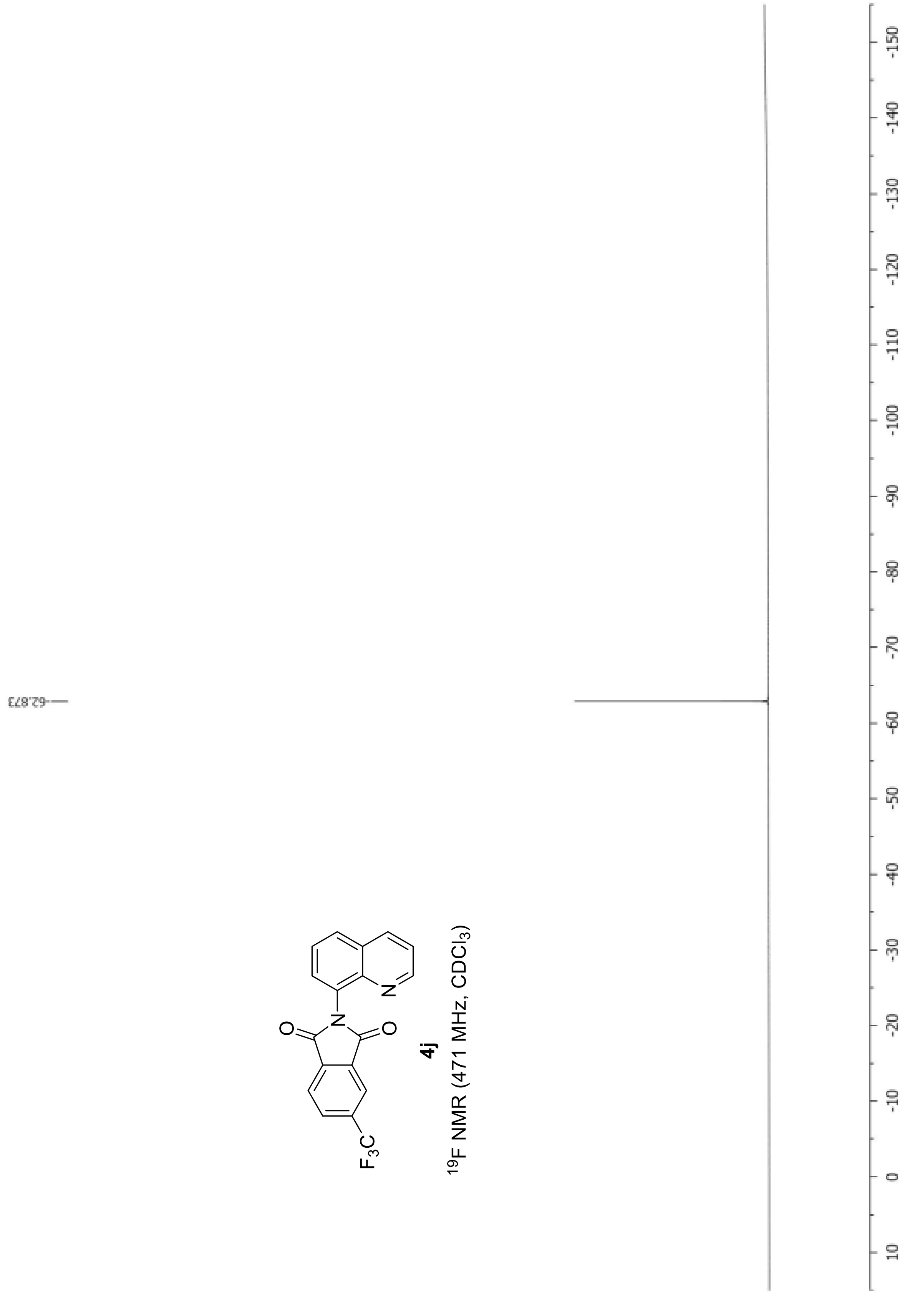

4j

^{19}F NMR (471 MHz, $CDCl_3$)

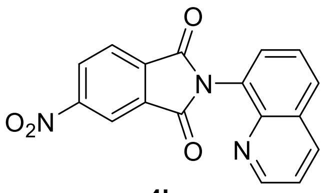

4k

^{1}H NMR (500 MHz, $CDCl_3$)

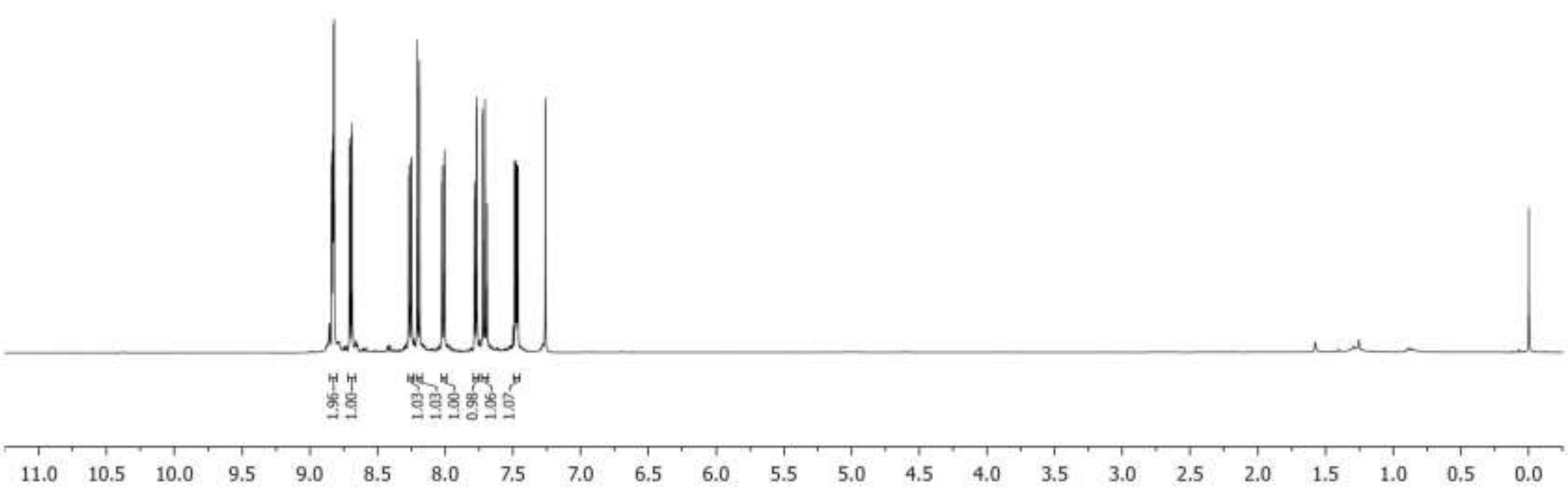

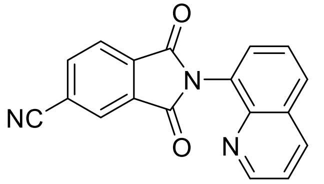

4l

^{1}H NMR (500 MHz, $CDCl_3$)

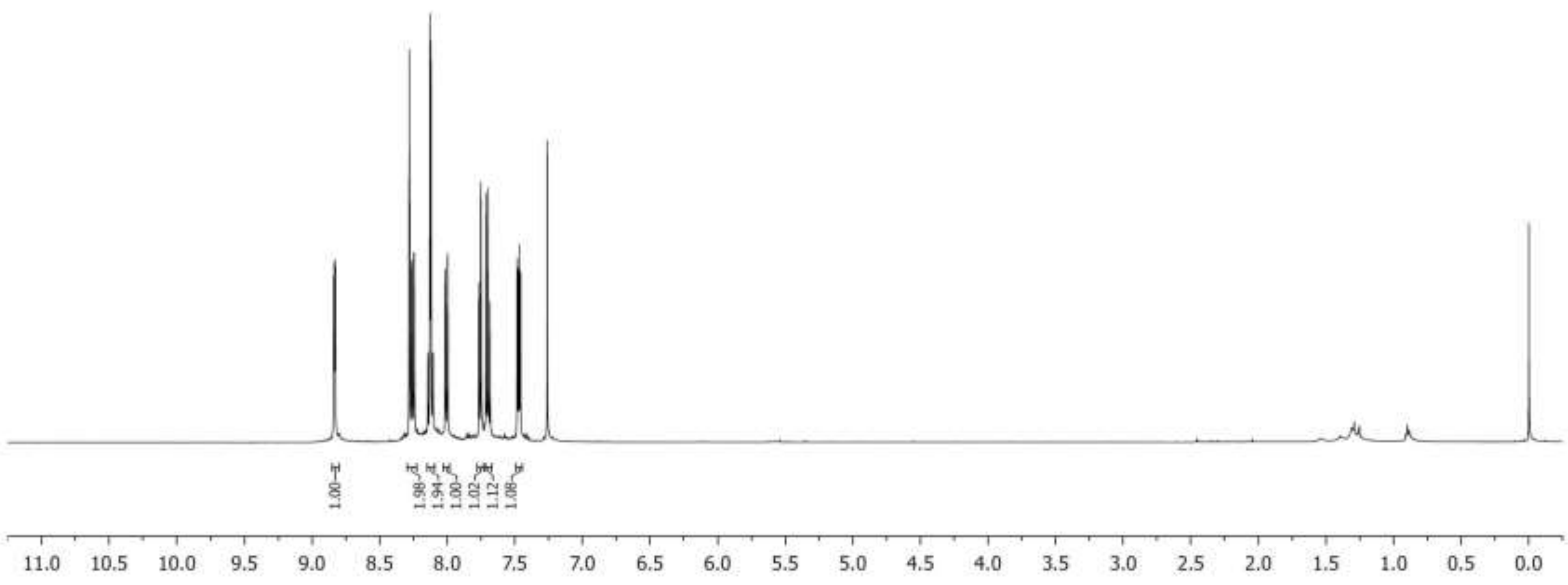

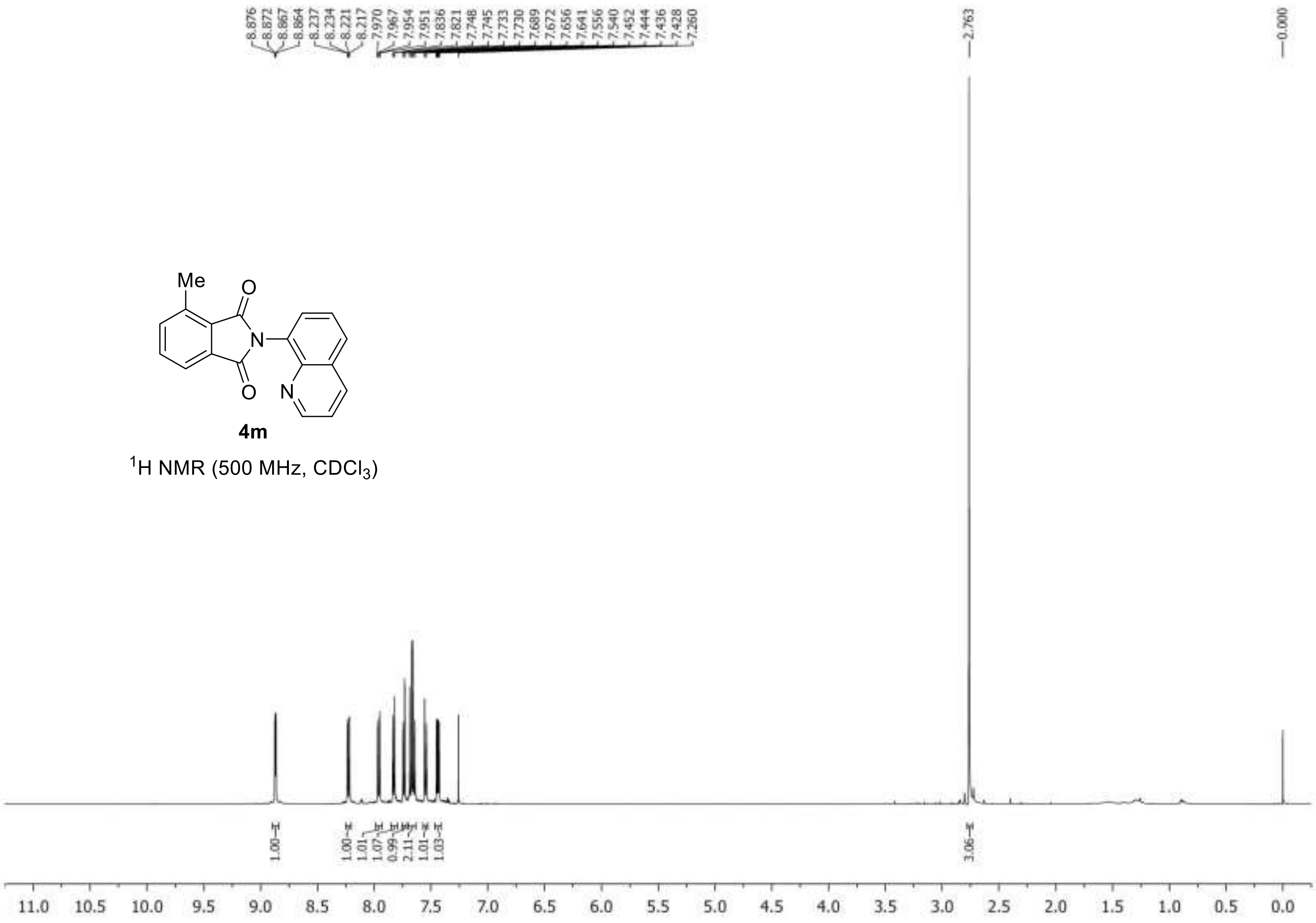

4m

^1H NMR (500 MHz, $CDCl_3$)

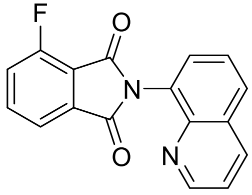

4n

^{1}H NMR (500 MHz, $CDCl_3$)

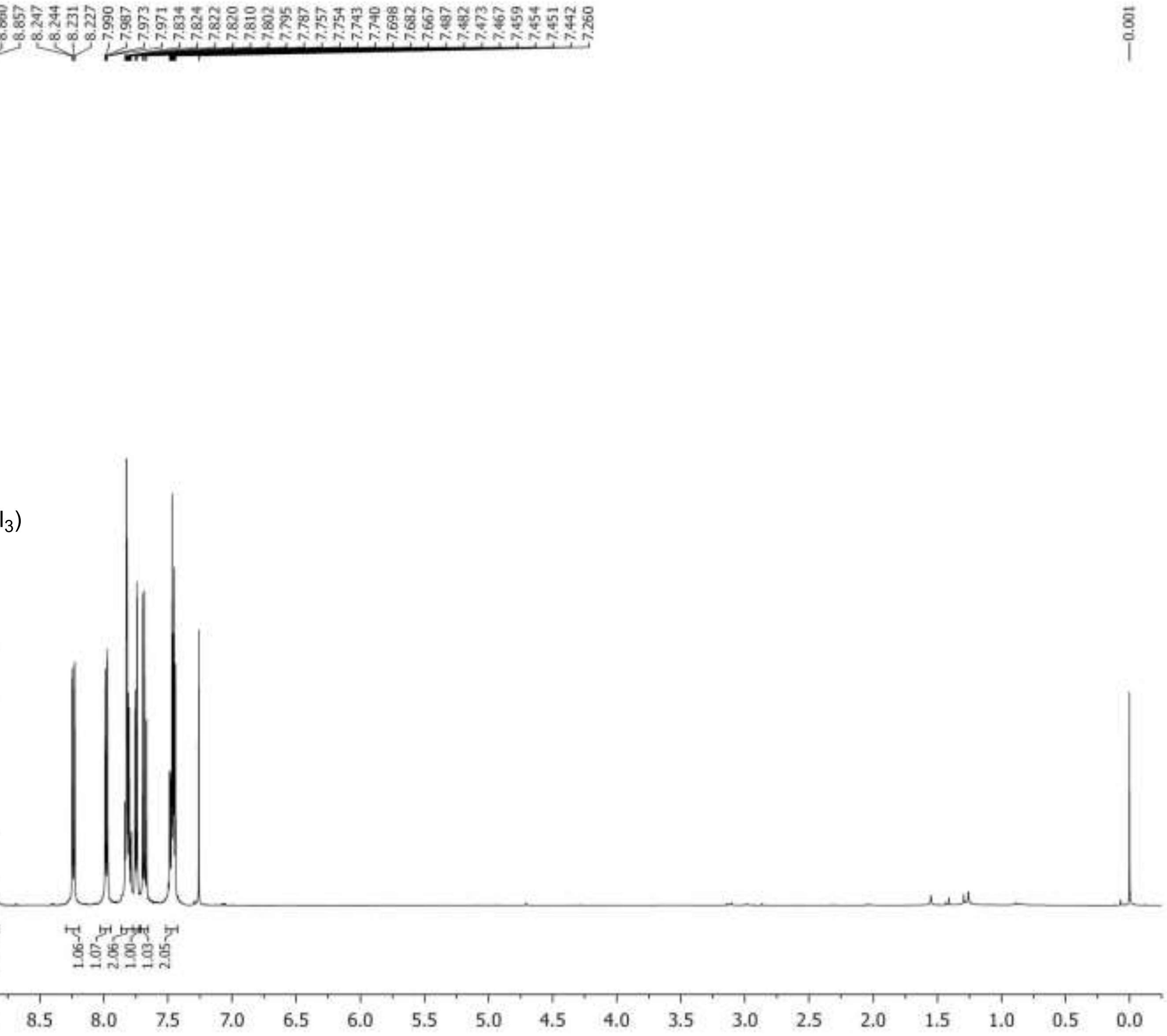

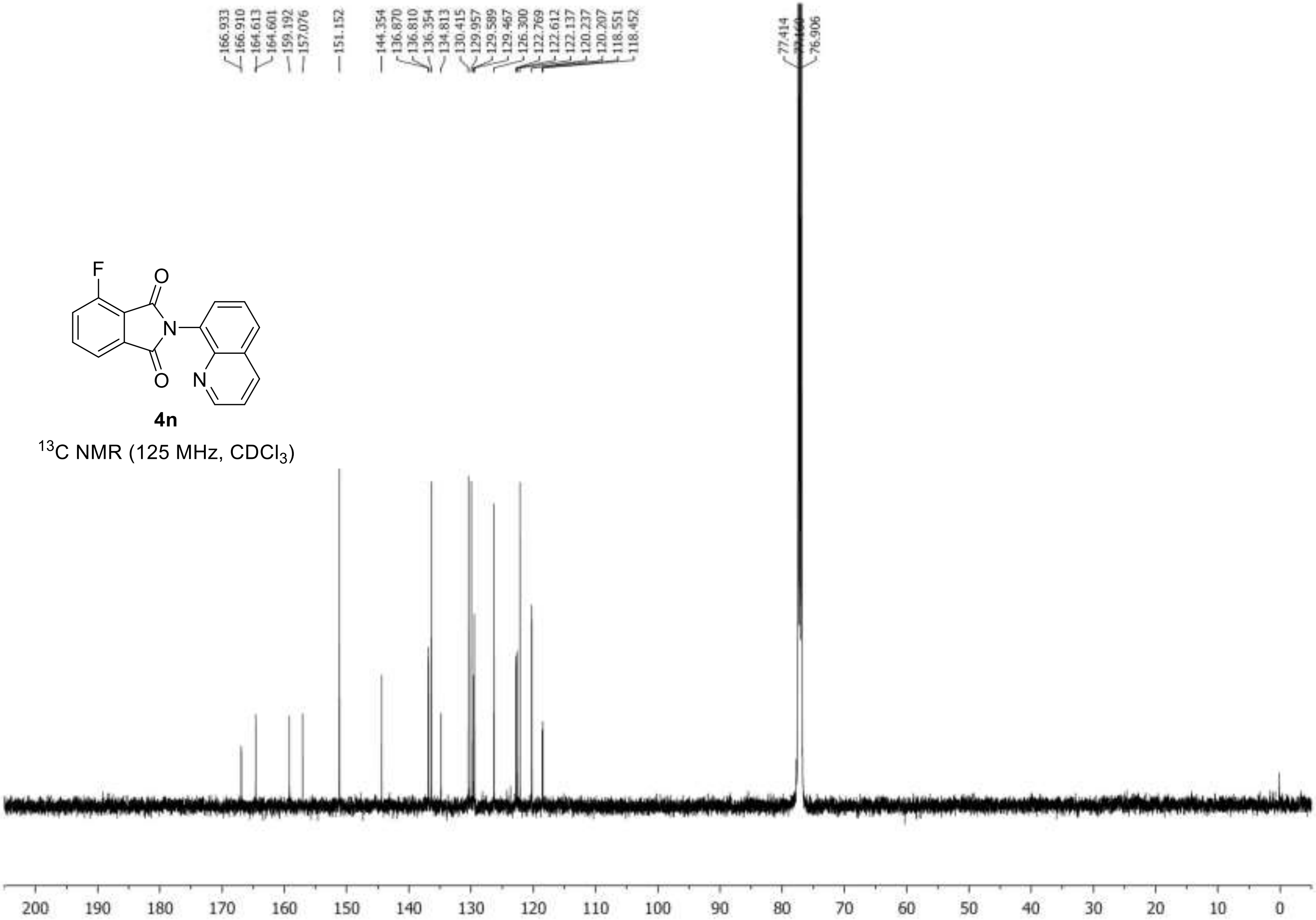

4n

^{13}C NMR (125 MHz, $CDCl_3$)

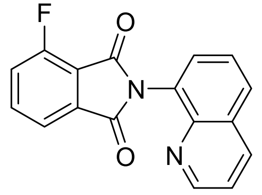

4n

^{19}F NMR (471 MHz, $CDCl_3$)

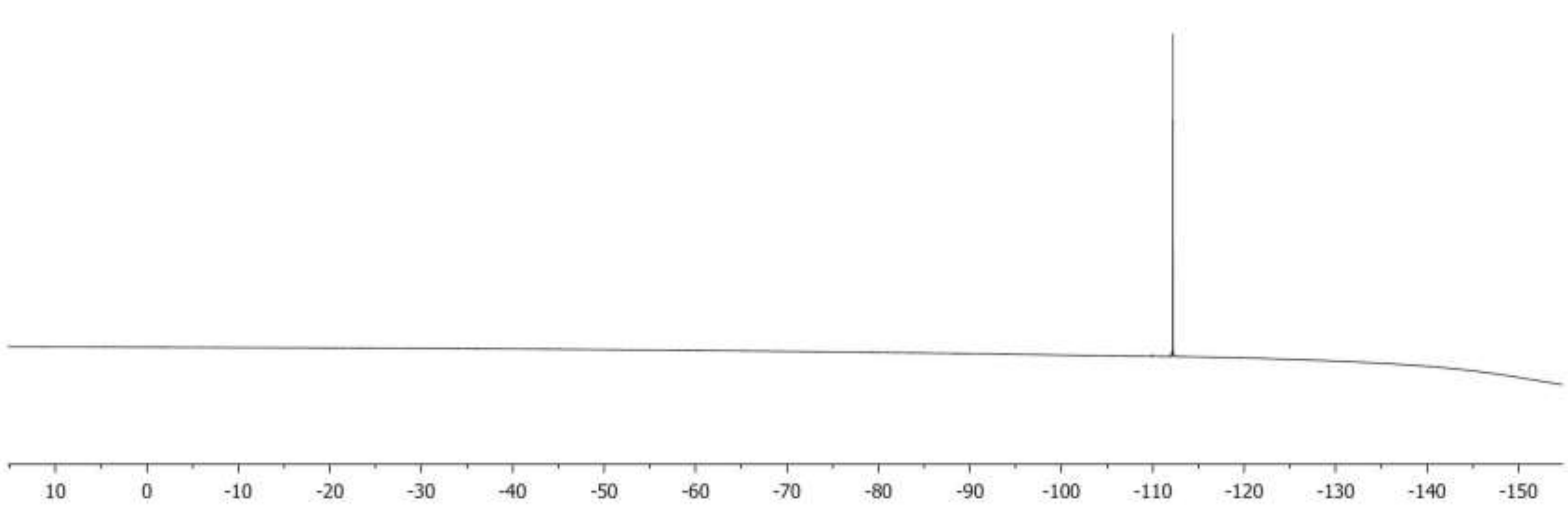

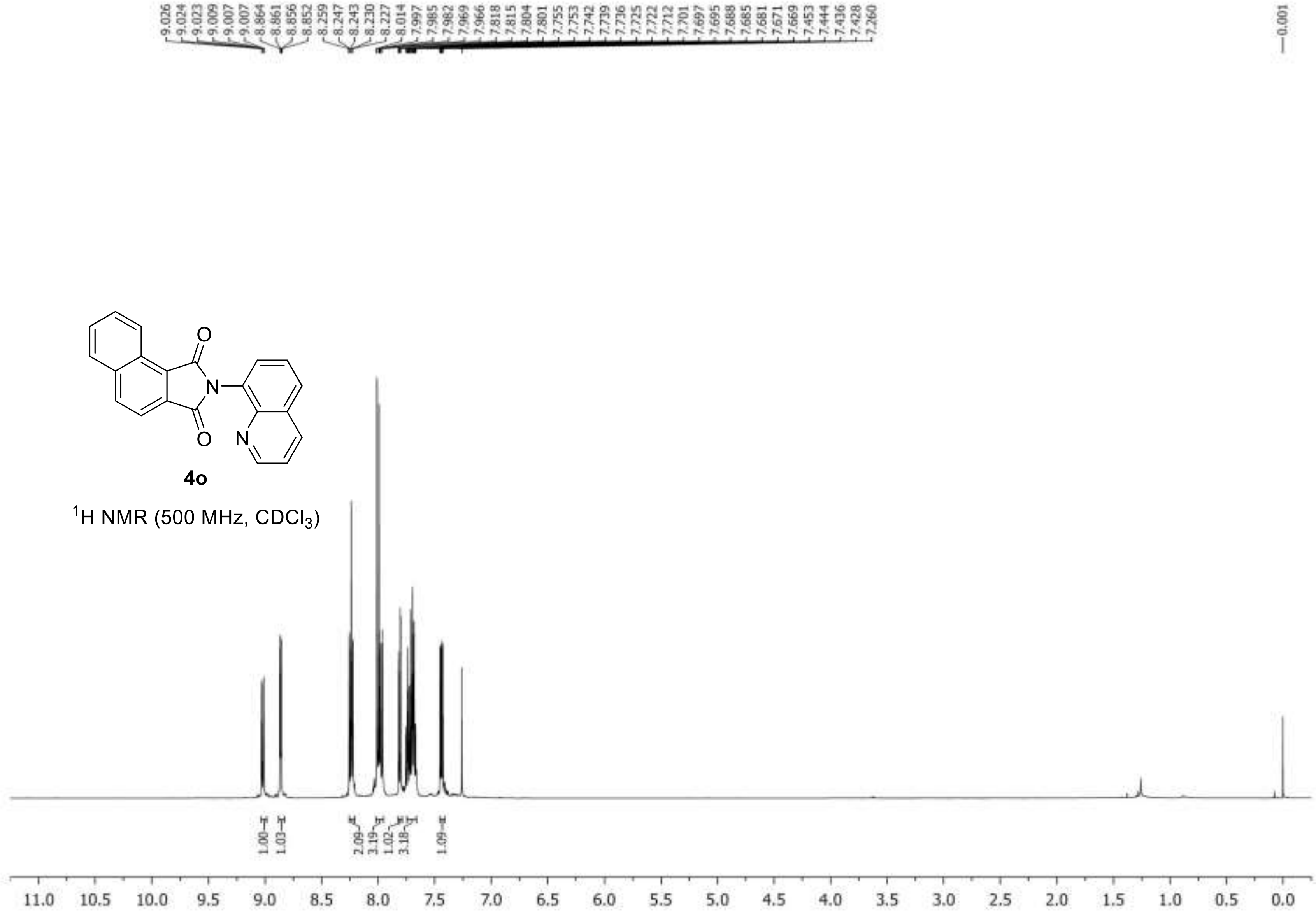

4o

^{1}H NMR (500 MHz, $CDCl_3$)

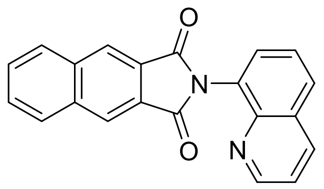

4p

^{1}H NMR (500 MHz, $CDCl_3$)

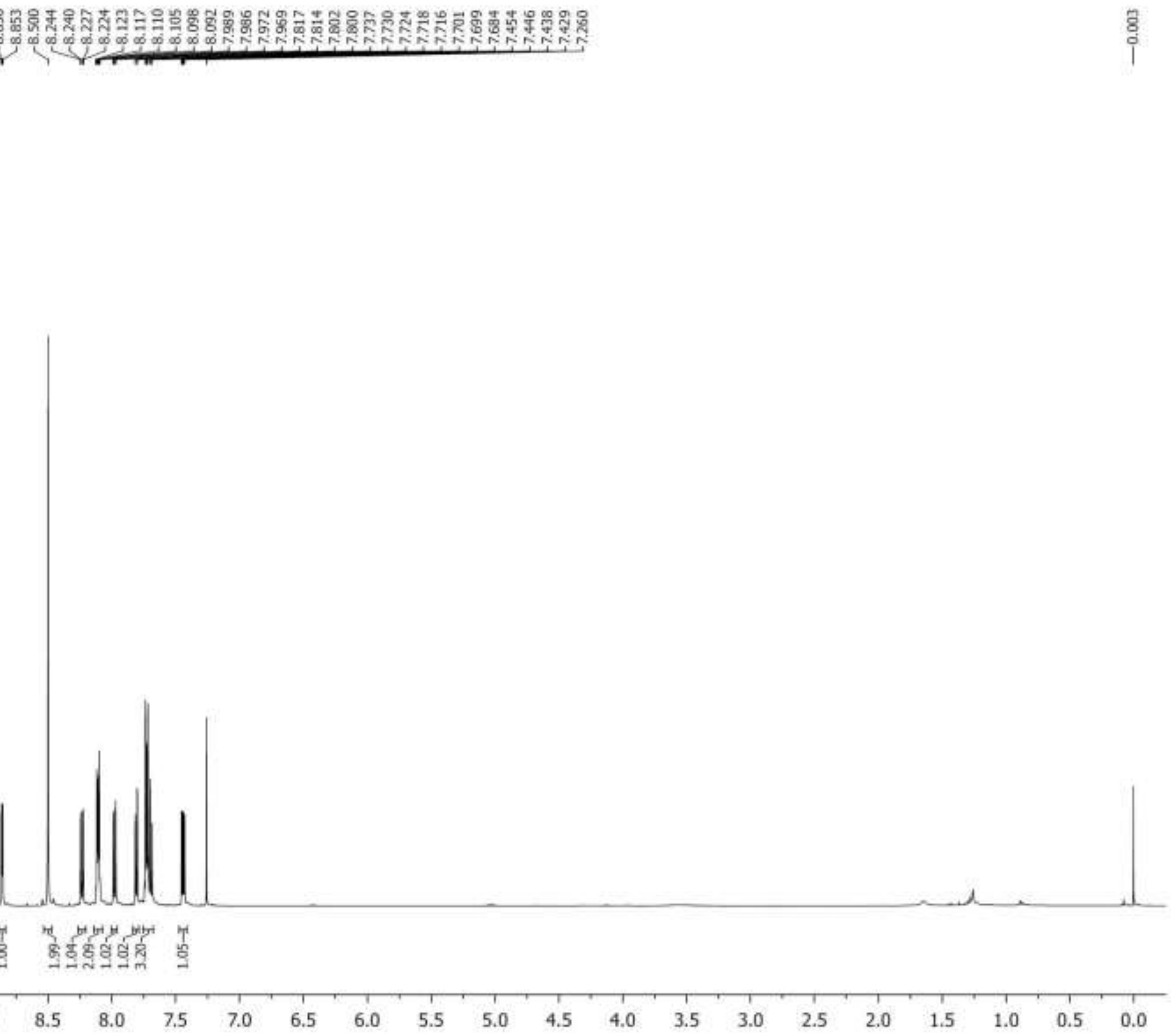

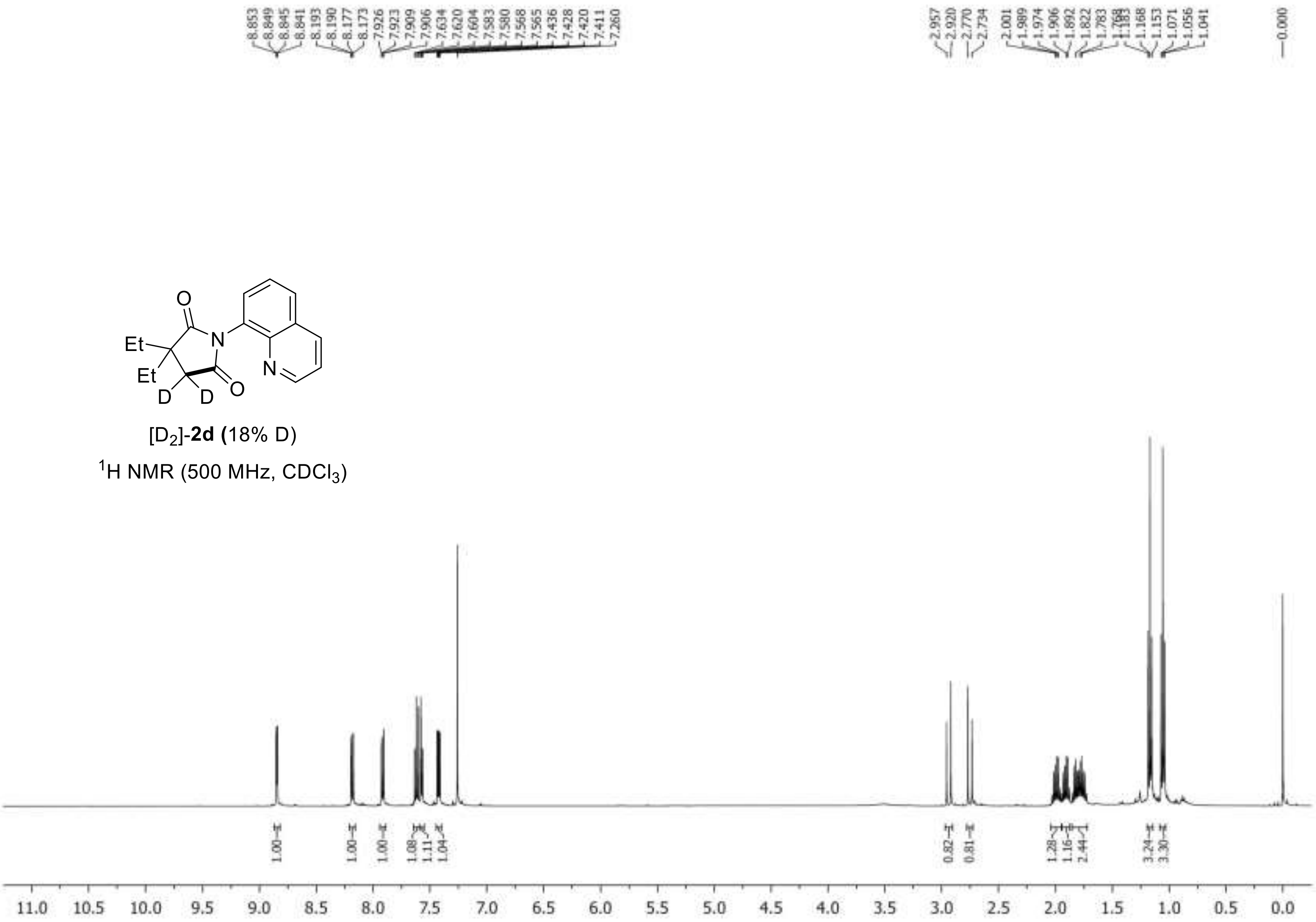

[D$_2$]-**2d** (18% D)
^{1}H NMR (500 MHz, $CDCl_3$)

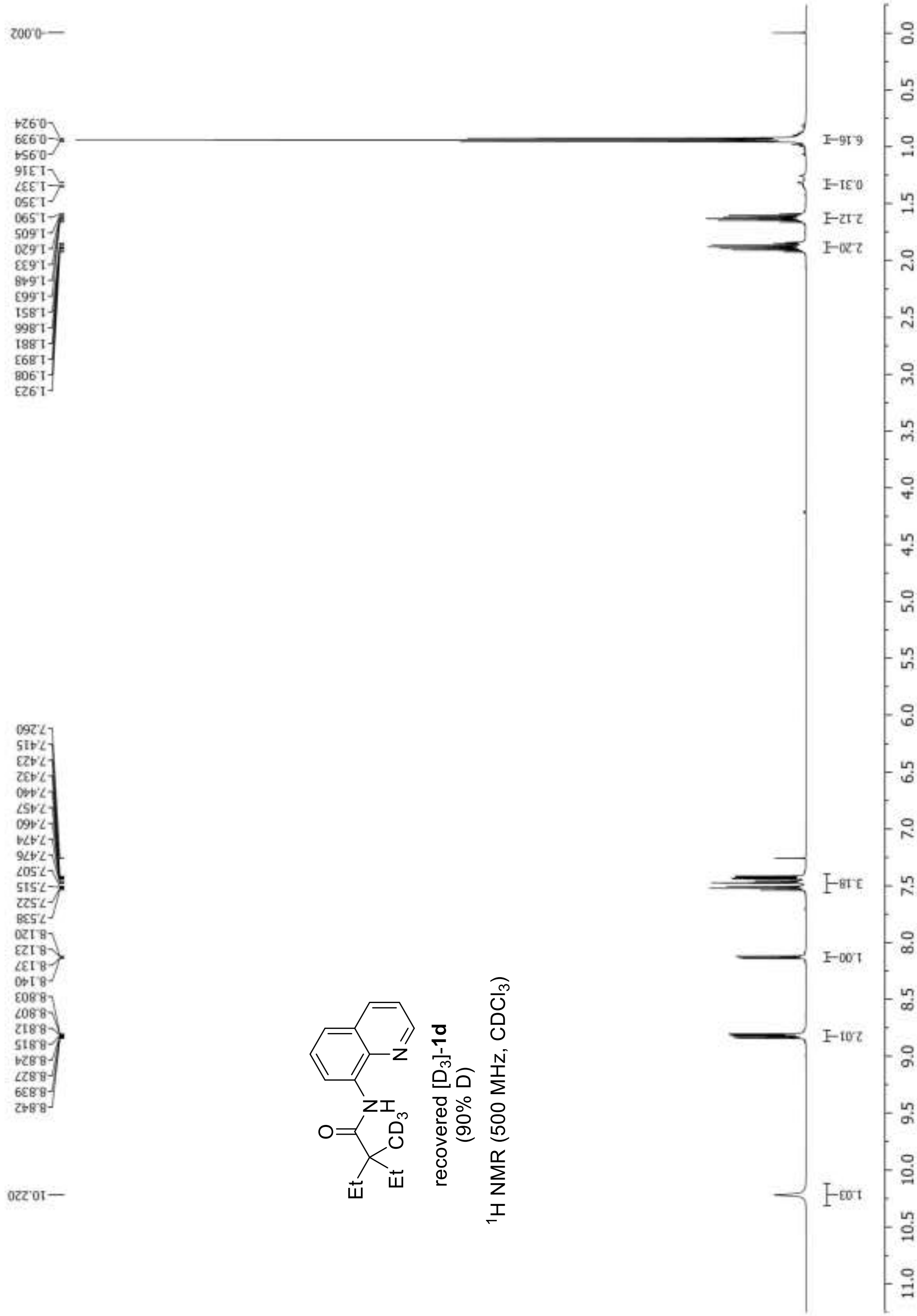

recovered [D$_3$]-**1d**
(90% D)
^{1}H NMR (500 MHz, $CDCl_3$)

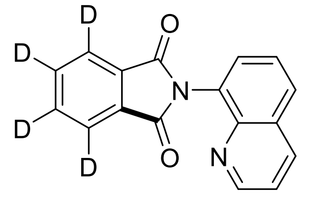

[D_4]-**4a** (>99% D)

^{1}H NMR (500 MHz, $CDCl_3$)

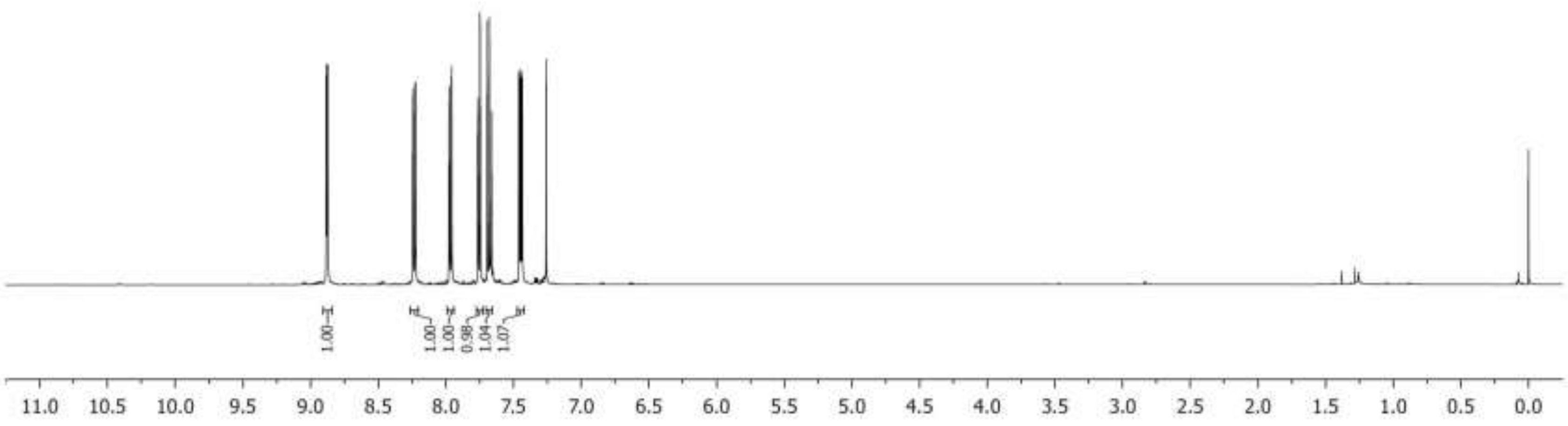

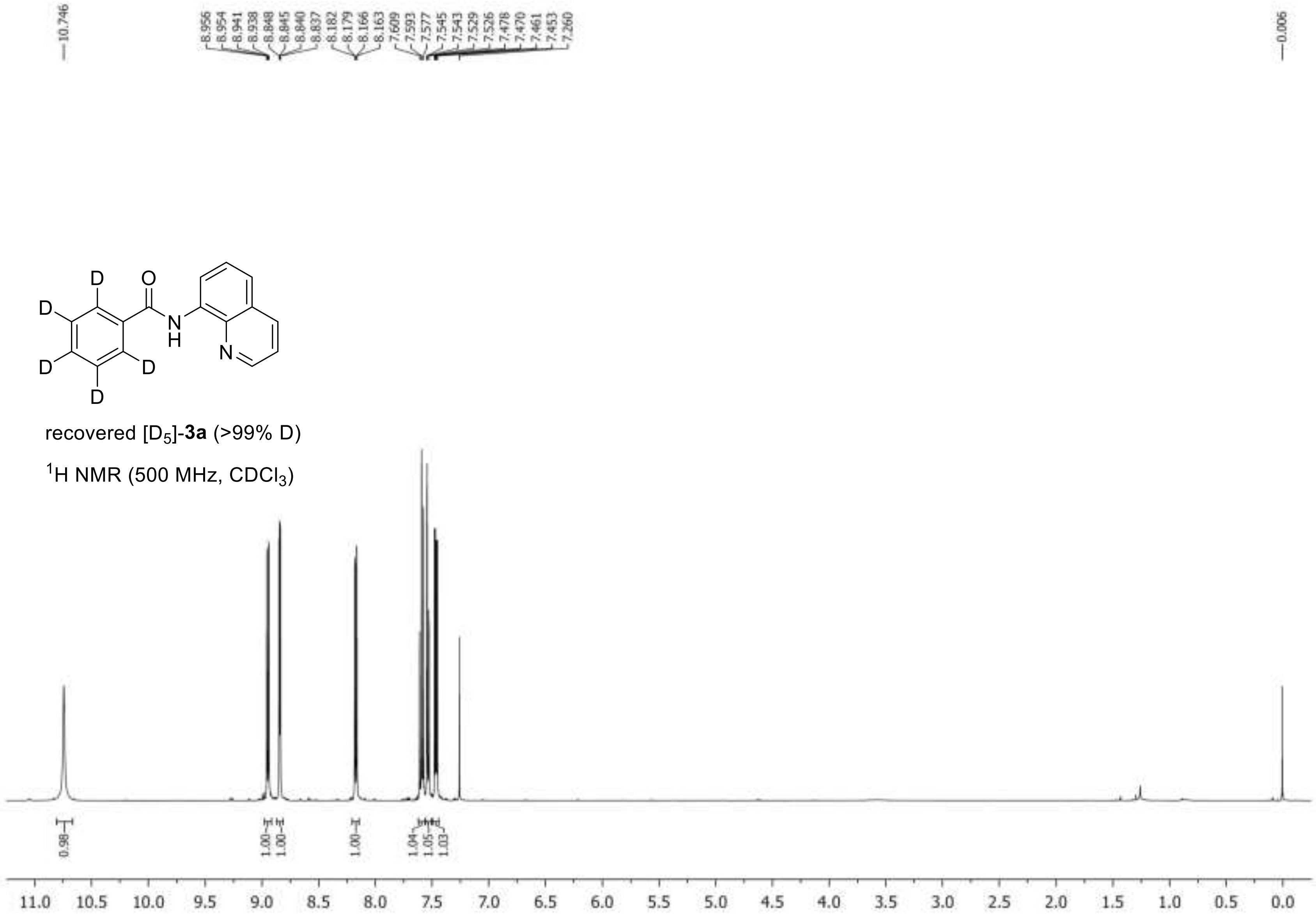

recovered [D_5]-**3a** (>99% D)

^{1}H NMR (500 MHz, $CDCl_3$)
